# Co-cultures of cerebellar slices from mice with different *reelin* genetic backgrounds as a model to study cortical lamination

**DOI:** 10.12688/f1000research.126787.1

**Published:** 2022-10-17

**Authors:** Adalberto Merighi, Laura Lossi

**Affiliations:** 1 Department of Veterinary Sciences, University of Turin, Grugliasco, 10095, Italy

**Keywords:** Reelin, Neuronal migration, Cerebellum, Purkinje neurons, Secreted proteins, Ex vivo methods, Cellular sociology, Voronoi tessellation

## Abstract

**Background:** Reelin has fundamental functions in the developing and mature brain. Its absence gives rise to the Reeler phenotype in mice, the first described cerebellar mutation. In homozygous mutants missing the Reelin gene ( *reln* ^-/-^), neurons are incapable of correctly positioning themselves in layered brain areas such as the cerebral and cerebellar cortices. We here demonstrate that by employing *ex vivo* cultured cerebellar slices one can reduce the number of animals and use a non-recovery procedure to analyze the effects of Reelin on the migration of Purkinje neurons (PNs).

**Methods:** We generated mouse hybrids (L7-GFP *reln*F1/) with green fluorescent protein (GFP)-tagged PNs, directly visible under fluorescence microscopy. We then cultured the slices obtained from mice with different *reln* genotypes and demonstrated that when the slices from *reln ^-/-^* mutants were co-cultured with those from reln ^+/-^ mice, the Reelin produced by the latter induced migration of the PNs to partially rescue the normal layered cortical histology. We have confirmed this observation with Voronoi tessellation to analyze PN dispersion.

**Results:** In images of the co-cultured slices from *reln ^-/-^* mice, Voronoi polygons were larger than in single-cultured slices of the same genetic background but smaller than those generated from slices of *reln ^+/-^* animals. The mean roundness factor, area disorder, and roundness factor homogeneity were different when slices from *reln ^-/-^* mice were cultivated singularly or co-cultivated, supporting mathematically the transition from the clustered organization of the PNs in the absence of Reelin to a layered structure when the protein is supplied *ex vivo.*

**Conclusions:** Neurobiologists are the primary target users of this 3Rs approach. They should adopt it for the possibility to study and manipulate *ex vivo* the activity of a brain-secreted or genetically engineered protein (scientific perspective), the potential reduction (up to 20%) of the animals used, and the total avoidance of severe surgery (3Rs perspective).

Research highlights **Scientific benefit(s):**

• Co-culturing slices from animals with different *reln* genetic backgrounds allows studying *ex vivo* the effects of Reelin in cortical lamination
• Co-cultures can be pharmacologically manipulated and transfected with different types of fluorescent reporter proteins (FRP)
• They are amenable to electrophysiological recordings and immunocytochemical labeling

**3Rs benefit(s):**

• As several viable slices can be obtained from every single animal, these cultures substantially reduce the necessary number of mice in different experiments
• When a secreted molecule is to be studied (such as in the case of Reelin), this approach can be used to replace *in vivo* experiments where the substance has to be administered through more or less invasive routes, involving heavy surgery for molecules that are unable to pass the blood-brain barrier

**Practical benefit(s):**

• As experiments *in vivo* are more expensive than those in *ex vivo*/ *in vitro* conditions, slice co-cultures are highly valuable in terms of cost *vs.* effectiveness
• They allow mid-throughput screening of different culture conditions, *e.g.*, days *in vitro*, the chemical composition of the medium, *etc.*, offering the possibility to save time and plan fewer *in vivo* confirmatory experiments, if necessary
• They are less technically demanding than *in vivo* experiments

**Current applications:**

• Study of the effect of *Reln* dosage on the differentiation of the laminated structures of the brain, primarily the cerebral and cerebellar cortex

**Potential applications:**

• Co-cultures can be used for the study of cerebellar neuronal wiring *ex vivo*, *e.g.*, to reconstruct in a dish the olivo-cerebellar tract (climbing fibers) by cultivating together slices from cerebellum and medulla oblongata
• Co-cultures can be used to study the development and/or neurodegeneration in other areas of the brain and spinal cord

## Introduction

Like any other animal tissue, the nervous tissue is made up of cells and the surrounding extracellular matrix. Proteins that are released from the neural cells consist of those in the extracellular matrix itself, as well as extracellular signaling and adhesion molecules. ^1^ Compared to neurons and neural precursor cells, glial cells release a comparatively modest amount of proteins with a narrower range of functions, and according to a two-dimensional (2D) gels and liquid chromatography/mass spectrometry study, about 22% of the proteins secreted by neural cells intervene in cell-to-cell interactions. ^1^

Reeler was the first discovered mouse cerebellar mutation, ^2^ it was distinguished by typical gait changes ("reeling"), and thus thereafter named. In recessive homozygous mutants ( *reln* ^-/-^), Reelin, a large secreted extracellular matrix glycoprotein, was completely absent and proved to be required for the normal development of layered brain structures ( *i.e.*, the cerebral and cerebellar cortices) being directly involved in neuronal migration. ^3^ Reelin absence causes severe cerebellar hypoplasia in *reln* ^-/-^ mice. During the development of the cerebellar cortex, the granule cells synthesize and release the molecule into the neuropil, and Reelin acts as an attractant for correct migration and placement of the Purkinje neurons (PNs). ^3^ Remarkably, only 5% of these neurons align into their typical location between the mature molecular and granular layers, 10% remain in the internal granular layer, and those left behind are distributed throughout the white matter of the medullary body in a rather compact central mass. ^4^^–^^6^ Differently from *reln* ^-/-^ mice, heterozygous *reln* ^+/-^, and homozygous *reln* ^+/+^ animals, do not display obvious disturbances in cortical histology, although the size and number of the PNs, as well as their topology, may be somewhat altered also in the former. ^7^

More recently, it was demonstrated that not only Reelin is implicated in neuronal migration but, after development, it intervenes in synaptogenesis, neuronal plasticity, ^8^^–^^10^ and several neuropsychiatric disorders. ^11^^–^^13^ In addition, as Reelin is somehow the prototype of the brain extracellular matrix proteins because of its widely demonstrated intervention in the process of neural migration, there is a wide interest in gaining more information about its role in the normal and pathological brain. Finally, it seems reasonable to hold that the development of a reliable method to study the effects of Reelin on neuronal migration on live cells would be of benefit to the study of many other secreted brain proteins that regulate cell-to-cell interactions.

Several approaches are available for the study of these proteins. Among those *in vitro*, one can, for example, mention the above proteomic study, which was carried out on cortical neurons and astrocytes, as well as cell lines that were derived from dividing neural precursor cells of E16 rats. ^1^ Other approaches have used *in vivo* microdialysis combined with proteomics to discover new bioactive neuropeptides in the striatum ^14^ or biopanning, an affinity selection technique that selects for peptides binding to a given target to identify proteins of the extracellular matrix. ^15^ These and other more sophisticated secretome studies, for example, ^16^ are very important in the initial identification of individual proteins in specific neural cell populations but do not offer any cues about their function and are not suitable to be used in longitudinal studies aiming to understand the effects of a given protein over time.

Longitudinal studies *in vivo* that are necessary to follow Reelin (and other brain-secreted proteins) intervention at different time points of development or in adulthood require a high number of animals at different ages to lead to conclusive and biologically relevant results. We here report on an *ex vivo* procedure to study the effect of Reelin on neuronal migration. Our procedure is based on the use of organotypic co-cultures of the mouse postnatal cerebellum ^17^ but can be broadly employed in the study of the biological role of this (and other) secreted molecule in the brain. Alternatively, one could use three-dimensional (3D) cultures, but a reliable reconstruction of neural circuits is still very difficult to achieve and one should very well know these circuits *in vivo* such is *e.g.*, the case of the retina. ^18^ Moreover, the approach is usually expensive, time-consuming, and cellular and biomolecular analysis difficult to perform. ^19^

The 3Rs relevance of our approach is primarily related to 1. The reduction of the number of experimental animals; 2. The refinement of the procedures eluding the administration *in vivo* of molecules with (potential) toxic effects and the use of heavy brain surgery ( *e.g.*, the intraventricular administration of substances that are unable to cross the blood-brain barrier and/or the need to implant osmotic pumps for sustained administration over time).

Potential end-users are neurobiologists chiefly interested in brain development and neurodegeneration from a structural, functional, and pharmacological point of view. Neuromodulation, *i.e.*, the continuous change of synaptic network parameters, is required for adaptive neural circuit performance. This process is primarily based on the binding of a variety of secreted “modulatory” ligands to G protein-coupled receptors, which govern the operation of the ion channels affecting synaptic weights and membrane excitability. ^20^ The possibility to also apply our approach to studies on neuromodulation substantially widens the number of potentially interested researchers and opens yet unexplored avenues to implement the 3Rs principles.

The need for 3Rs research in these fields is supported by quantitative data. It is difficult to give an accurate estimate of the number of animals used for the purpose locally and worldwide. Yet one can reasonably hold that at least a 20% reduction (a figure based on the number of slices that can usually be generated per mouse) in their total number could be achieved by the adoption of this (and other) procedures *ex vivo*, as discussed in Ref. 17.

With specific regard to Reelin activity in normal and pathological conditions, a PubMed search (June 2022) with the string “reelin brain” gives back 1,499 results with a peak of 98 papers published in 2010 and a mean of 57 papers/year starting from 1993 (year of the first publication). A similar number of papers is retrieved from the Web of Science™ (1,349) and groups that have published at least two papers belong to 46 different countries. One has to consider that these figures increase substantially if the search is widened to secreted proteins more generally ( *i.e.*, 3,154 papers in PubMed for the string: secreted proteins AND “cell migration” AND brain). Although not often easy to glean from the Material and Methods section, in a typical publication *in vivo*, animal number ranges from 50 to 80 depending on the types of experiments, the number of experimental groups, and the approaches used.

The severity classification of our procedure as defined under the Directive 2010/63/EU is non-recovery.

## Methods

### Materials and methods

*Mouse model*

Ethical statement

All experimental procedures described here have been approved by the Italian Ministry of Health (n. 65/2016-PR del 21/01/2016 and n.1361.EXT.1 del 27/12/2016) and the Bioethics Committees of the University of Turin and the Department of Veterinary Sciences (DSV). The number of animals (8 *reln* ^-/-^ and 8 *reln* ^+/-^) was kept to a minimum and all efforts were made to minimize their suffering.

Mouse housing and husbandry

Animals were housed in the facility of DSV under the following conditions: temperature 19–21 °C, humidity 55% ± 10%, light-dark cycle 12-12 h. Food (normal maintenance diet – meat-free rat and mouse diet SF00-100, Specialty Feeds, Glen Forrest Western Australia) and water (normal tap water) were given *ad libitum.* The bedding was non-sterile wood-chip. Environmental enrichment consisted of mini tubes, sizzle nests, and burrowing treats (Volkman Seed Small Animal Rodent Gourmet). Animals were bred in couples in a standard 484 cm ^2^ mouse cage. The mice themselves were not health-screened, the animal enclosure was free of the major rodent pathogens but some sentinels were positive for adventitious agents, *i.e.*, mouse hepatitis virus after indirect fluorescent antibody (IFA) test and Multiplexed Fluorometric ImmunoAssay (MFIA) and *Entamoeba* sp. after annual mouse health monitoring (HM) Federation of European Laboratory Animal Science Associations (FELASA) screen.

Generation of L7-GFPrelnF1/mouse hybrids

Hybrids (L7-GFP *reln*F1/) were generated by crossing L7-green fluorescent protein (GFP) (RRID:IMSR_JAX:004690) female mice (L7GFP ^+/+^) with Reeler heterozygous ( *reln* ^+/-^) male mice (RRID:IMSR_JAX:000235). ^7^ L7GFP ^+/+^ mice express GFP under the control of the L7 promoter. ^21^^,^^22^ As the L7 gene is specifically expressed by the PNs, these neurons are tagged by GFP, allowing their visualization without the need for immunocytochemical labeling. Before use, all animals were sexed and genotyped by routine methods to ascertain GFP expression ( *Note 1*) and their appropriate *reln* genetic background. ^3^

*Methods for the model development*

Preparation of organotypic co-cultures from L7-GFPrelnF1/of different genetic backgrounds

Experiments are reported in compliance with the ARRIVE guidelines, ^42^ including randomization of samples in culture inserts (slices in a single insert came from different mice), blinding of the experimenter who performed image analysis with unblinding at end of image processing, and/or automation of quantification, as indicated in the following sections.

**Figure 1.**
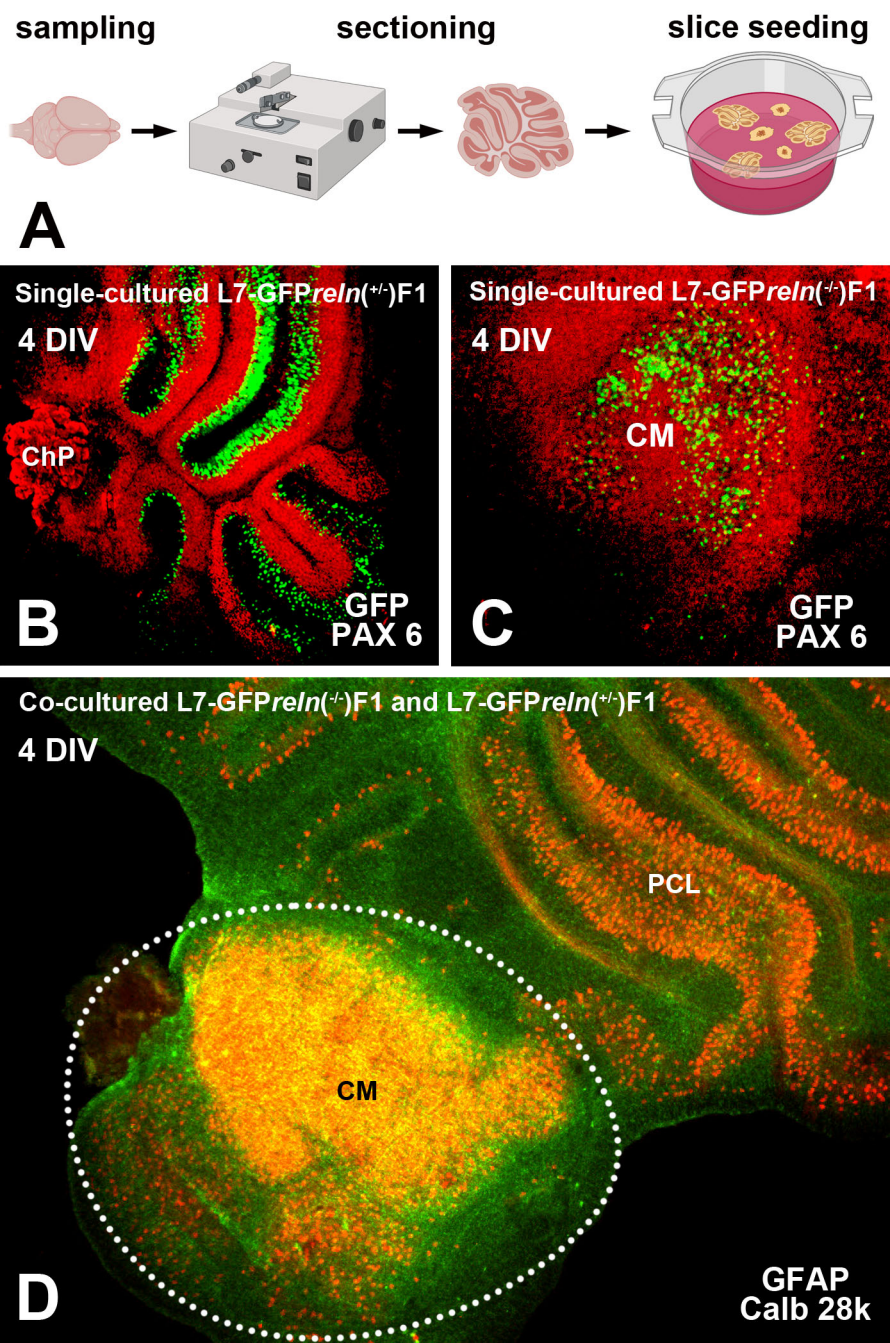
General features of cerebellar cultures. A: Flowchart showing the main steps for co-culture preparation (see protocols.io for details). B-D: Histological aspects of four DIV single- and co-cultured slices after immunostaining with different markers of cerebellar neurons and glia. B-C: Immunostaining of the cerebellar granule cells with PAX6 permits clear identification of these neurons (red) and the GFP-tagged PNs (green). D: Two co-cultured slices with different *reln* genetic backgrounds. The slices have originally plated at a distance from each other but tend to expand with time *in vitro* and thus are in contact in this image. The slice from an L7-GFP *reln* ^-/-^F1/mouse is indicated by the dotted white line. Note that in D PNs have been stained for calb 28k and thus appear yellowish-orange for the superimposition of the green GFP signal and the red calb 28k fluorescence. *Abbreviations*: calb 28k = 28kD calbindin; ChP = choroid plexus; CM = central mass; DIV = days *in vitro*; GFAP = Glial fibrillary acidic protein; GFP = green fluorescent protein; PAX-6 = paired-box protein PAX6; PCL = Purkinje cell layer; PNs = Purkinje neurons.

A step-to-step protocol for the preparation of cerebellar organotypic cultures ( Figure 1A) has been deposited on protocols.io ( https://dx.doi.org/10.17504/protocols.io.6qpvr67bbvmk/v1). This protocol is a refinement of previously published procedures from our laboratory. ^23^^,^^24^

In the co-culture protocol, slices from *reln* heterozygous ( *reln* ^+/-^) and homozygous ( *reln* ^-/-^) hybrid mice were plated together. The positions of each genotypically identified slice in the insert were recorded so that they could be identified and monitored for the entire duration of the experiments ( *Note 2*). Slices in each insert were numbered in a clockwise direction starting from a point indicated by a permanent mark on the bottom of the plastic dish and in the following analysis, the experimenter remained unaware of the matching between the slice number and the genotype of the donor mouse.

Qualitative analysis of PN migration

**Figure 2.**
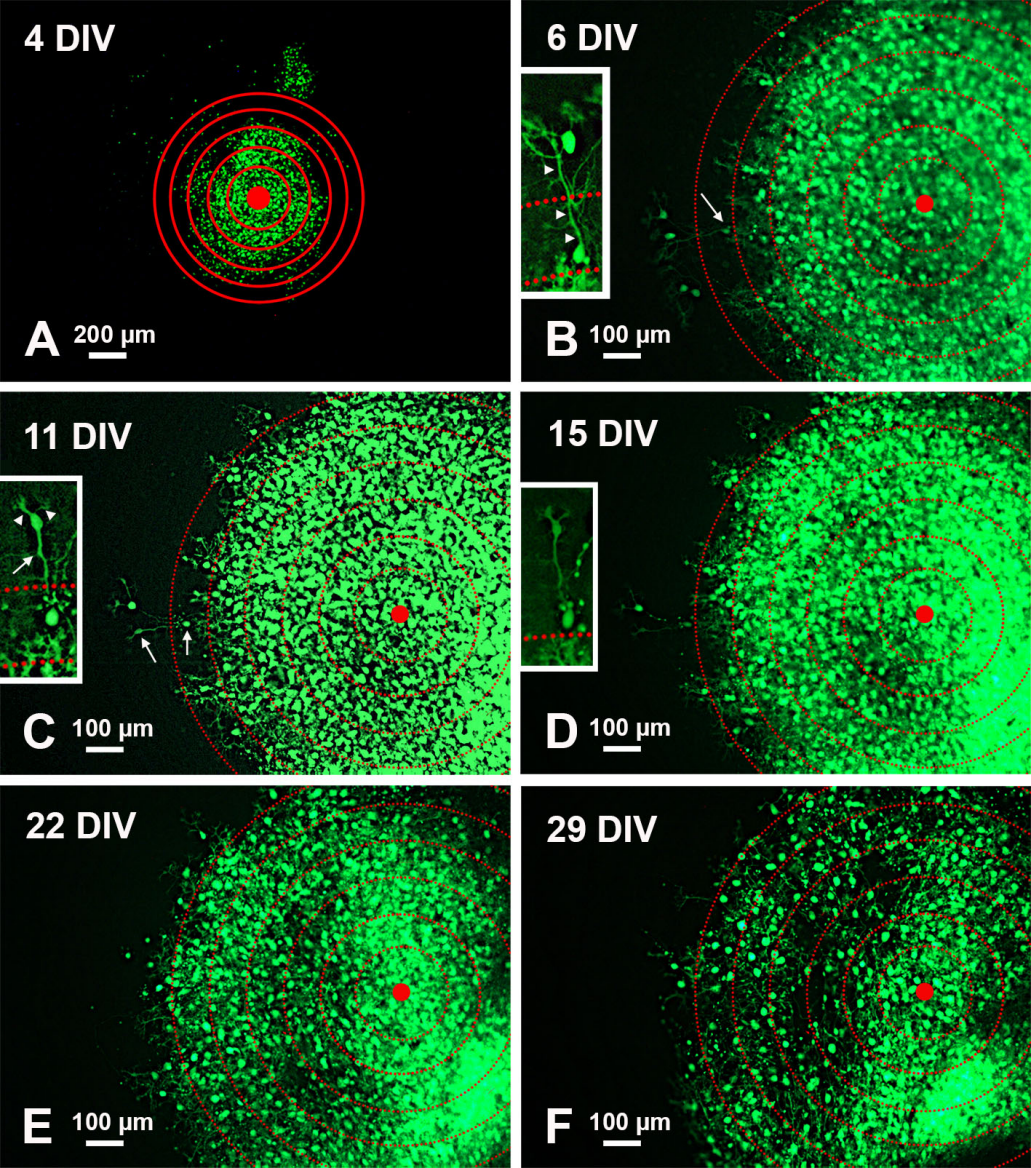
Temporal modifications of a single-cultured slice from an L7-GFP *reln* ^-/-^F1/mouse. A: Low magnification view of the slice with superimposed concentric circles surrounding the center of the slice. B-F: Higher magnification of the same slice and its evolution over time. The apparent dispersion of the PNs is mainly due to the death of individual cells. The inserts in B-D show the histological features of two PNs at the periphery of the central mass. Note the reduction of fluorescence in D and the disappearance of the two cells in E-F. *Arrows* in the main panels point to the PNs shown in the inserts at higher magnification. Arrows in the inserts indicate the PN axon, arrowheads the main dendrites. *Abbreviations*: DIV = days *in vitro*; GFP = green fluorescent protein; PNs = Purkinje neurons.

To analyze PN migration, organotypic cultures were photographed under a transmitted fluorescence light microscope. A series of six concentric circles spaced by 100 μm was superimposed on each photograph and roughly centered to the geometric center of the slice ( Figure 2).

Immunocytochemistry

A step-to-step protocol for the immunofluorescence staining of organotypic cultures can be found in Ref. 23. In this report, we simply show exemplificative staining with a rabbit anti-glial fibrillary acidic protein (GFAP – astrocytic marker) polyclonal antibody (Abcam Cat# ab7260, RRID: AB_305808), a mouse anti-28kD calbindin (a marker of the PNs) monoclonal antibody (Abcam Cat# ab9481, RRID: AB_2811302), and with a mouse anti-paired-box protein PAX6 (PAX6 – a marker of the differentiating granule cells) monoclonal antibody (Santa Cruz Biotechnology Cat# sc-81649, RRID: AB_1127044). All antibodies were used at dilutions ranging from 1:100 to 1:200.

Microscopy and photography

Cultures were photographed directly under a 10× or 20× objective of a Leika DM 6000 transmitted light microscope taking care not to expose them to environmental contaminants. Alternatively, they have been maintained in a microscope stage incubator fitted to a Leika SP5 Laser confocal microscope and photographed with a 20× lens (see *Note 2*).

#### Notes

**Figure 3.**
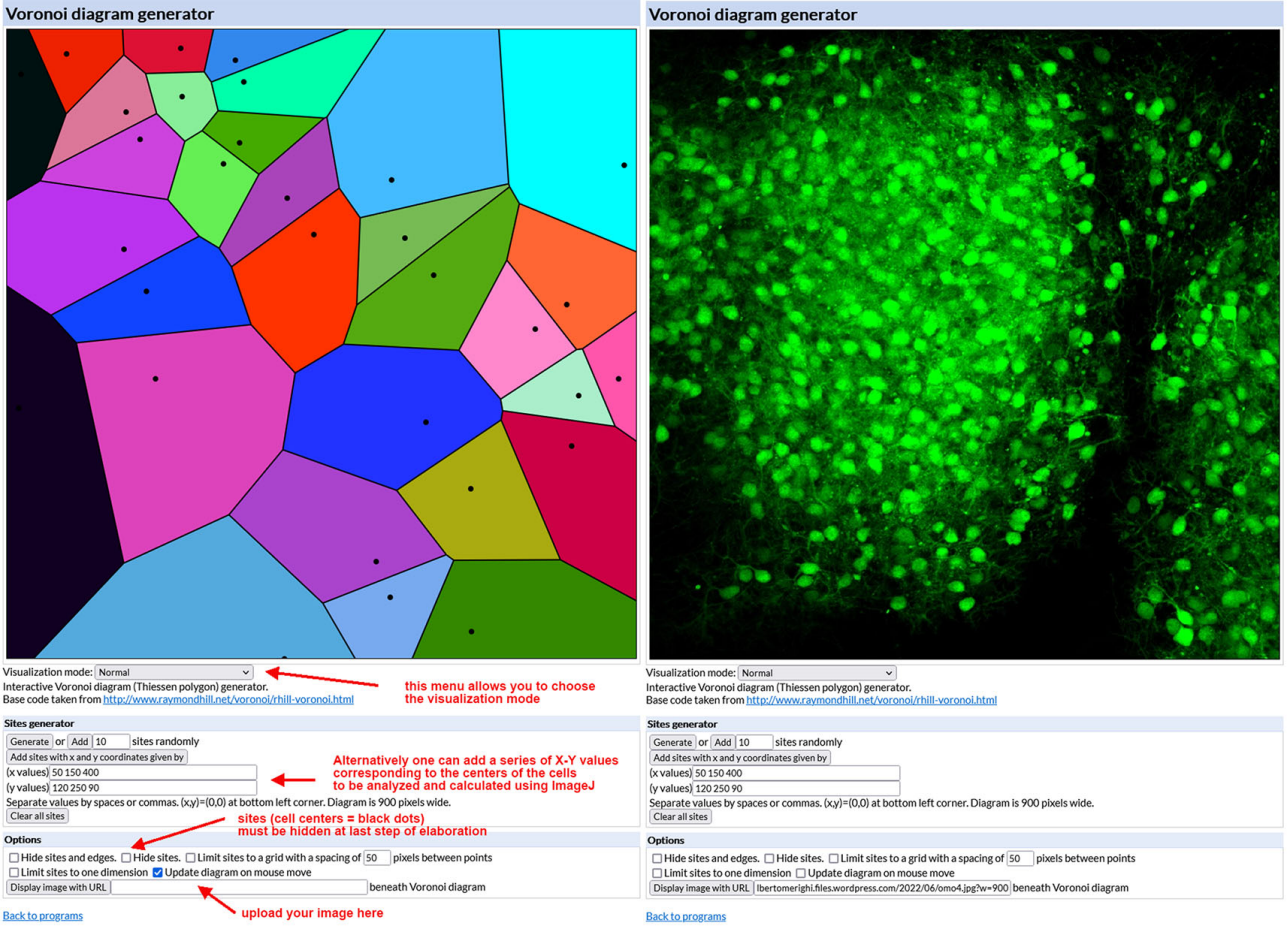
Use of the Voronoi diagram generator. *Left* – The mask of the Voronoi diagram generator with indications of its main commands and some hints for image elaboration. *Right* – The diagram generator with an uploaded example image of a single-cultured cerebellar slice from an L7-GFP *reln* ^-/-^F1/mouse. GFP = green fluorescent protein.

1. Genotyping was done by sampling a small piece of the pinna so that at the same time it was possible to identify the subject and extract the genomic DNA.
2. Although not strictly necessary, one can use an incubator that is fitted to the microscope stage (see *e.g.*, Figures 2 and 3 in Reference 25) to longitudinally monitor cultures and easily take photographs of the same slice so that individual microscopic fields can be easily recognized. Although this is ideal when it is necessary to pharmacologically challenge the cultures over time, it may be unpractical when several cultures must be processed together such is the case of the co-culture protocol here described.

*Methods for the characterization and validation of the model*

Cell dispersion was analyzed using Voronoi tessellation ^26^ aiming at demonstrating the precise relationship with cultural conditions, as this cellular sociology approach has proved useful in other biological contexts. ^27^

The numbers of technical repeats (individual slices from a single cerebellum) and independent biological repeats (organotypic cultures/co-cultures made by adding 3–6 individual slices to a single culture dish) are indicated in figure legends.

Cultures were obtained from two groups of mice: L7-GFPreln ^(+/-)^F1/ (n = 5) and L7-GFPreln ^(-/-)^F1 (n = 5). Cerebellar slices from these animals were subdivided into three groups:

1) single cultured L7-GFPreln ^(+/-)^F1/ slices, 2) single cultured L7-GFPreln ^(-/-)^F1/ slices, and 3) co-cultured L7-GFPreln ^(+/-)^F1/ slices + L7-GFPreln ^(-/-)^F1/ slices. In co-cultures, the ratio of L7-GFPreln ^(+/-)^F1/to L7-GFPreln ^(-/-)^F1/slices was 2:1/3:1. At least eight slices from each group were used for the analysis of cellular sociology (see below). The sample size (number of slices) was calculated using the G*Power calculator 3.1.9.4. ^28^ Input parameters were (unpaired t-test power calculator): tails, Two; parent distribution, Normal; α error probability, 0.05; power (1-β error probability), 0.95; effect size d, 2. The effect size was considered high based on qualitative observations and considering the mean area of the Voronoi polygons as the primary outcome. Output parameters were: non centrality r δ, 3.9088201; critical t, 2.1557656; Df, 13.2788745; sample size group 1, 8; sample size group 2, 8; Total sample size, 16; actual power, 0.9508778.

The eight slices/group of mice were randomly selected after sectioning the cerebella of at least five different animals.

The detailed procedure for calculation of Voronoi diagrams has been deposited on protocols.io ( https://dx.doi.org/10.17504/protocols.io.yxmvmnrx6g3p/v1).

The parameters extracted from the analysis were: the mean area of Voronoi polygons (forms), the average roundness factor of those forms (RFav), the measure of the roundness factor homogeneity (RFH), and the measure of their area heterogeneity (area disorder (AD)). The last three parameters are characteristic of the population topography. ^26^

### Statistics

We have used GraphPad Prism (RRID:SCR_002798) version 9.0.2 for Windows, GraphPad Software, San Diego, California USA, to assess the variations in the mean areas of Voronoi polygons, and RFav using a 95% confidence interval. Data were checked for outliers with the ROUT method (Q = 1) and normality with the Kolmogorov-Smirnov test. Further details of the tests used are given in figure legends. Inferential statistics were performed using ordinary one-way ANOVA followed by Tukey’s multiple comparison tests when data had a Gaussian distribution. The Brown-Forsythe test for equality of means and the Welch test was used for data sampled from populations with different variances.

### Protocols

*Protocol for establishing the co-culture model*

The protocol below describes the step-by-step procedure required to establish and validate the long-term co-cultures of the postnatal murine cerebellum. With minimal modifications, it could be adapted to co-cultures of other areas of the brain, *e.g.*, the hippocampus and entorhinal cortex or the cerebellum and the medulla oblongata containing the caudal (inferior) olivary nucleus. It stemmed from the single culture protocol previously developed in our laboratory. ^23^

Equipment

• Surgical instruments for brain dissection: universal scissors (length 13 cm), fine scissors straight and curved, Adson forceps, student anatomical standard pattern forceps, Dumont #7 forceps, gross anatomy blade (#20) and handle (#4), straight and curved spatulas, razor blades
• Dissecting microscope, *e.g.*, Stereo microscope EZ4, Leica 10447197
• CO _2_ incubator, *e.g.*, Certomat CS-18 Sartorius BBI-8863385
• McIlwain tissue chopper with Petri dish modification Campden Instruments Model TC752-PD – see *Note 1.*
• Millicell-CM ^®^ Cell Culture Inserts, 30 mm, hydrophilic PTFE, 0.4 μm, Merck, PICM0RG50
• Sterile 35-mm Petri dishes
• Nalgene ^®^ vacuum filtration system, filter capacity 1000 mL, pore size 0.2 μm, Sigma-Aldrich, Z358207
• 500 μL disposable insulin syringes
• Sterile glass/disposable Pasteur pipettes
• Sterile filter paper dishes

Chemicals

• Pentobarbital sodium, Sigma-Aldrich, Y00021941
• D-(+)-Glucose, Sigma-Aldrich, G8270
• L-Ascorbic acid, Sigma-Aldrich, A92902
• Pyruvic acid, Sigma-Aldrich, 107360
• N-Methyl-D-glucamine, Sigma-Aldrich, M2004
• Sodium bicarbonate, Sigma-Aldrich, S5761
• Potassium chloride, Sigma-Aldrich, P3911
• Sodium phosphate monobasic, Sigma-Aldrich, S0751
• Calcium chloride, Sigma-Aldrich, C1016
• Magnesium chloride, Sigma-Aldrich, M8266
• Basal Medium Eagle, Sigma-Aldrich, B9638
• Horse serum, Sigma-Aldrich, H1138
• Hanks′ Balanced Salt solution, Sigma-Aldrich Catalog, H6648
• L-Glutamine solution, Sigma-Aldrich Catalog, G7513
• Antibiotic Antimycotic Solution (100×) Stabilized, Sigma-Aldrich, A5955
• Paraformaldehyde, powder, 95%, Sigma-Aldrich, 158127

**Step 1:** Preparation of solutions and culture medium (see *Note 2*)

*1a. Stock solutions*: 1 M CaCl _2_; 1 M MgCl _2_; 5% volume pentobarbital sodium in ddH _2_O.

*1b. Cutting solution:* 130 mM n-methyl-D-glucamine Cl (NMDG); 24 mM NaHCO _3_; 3.5 mM KCl; 1.25 mM NaH _2_PO _4_; 0.5 mM CaCl _2_; 5 mM MgCl _2_; 10 mM D-(+)-glucose; 1 mg/mL ascorbic acid; 2 mg/mL pyruvic acid.

To make 1 L, pour 850 mL of double-distilled water into a volumetric flask. Add 25.38 g NMDG, 2.017 g NaHCO _3_, 261 mg KCl, 172 mg NaH _2_PO _4_, 1.80 g D-(+)-glucose, 1 g ascorbic acid, 2 g pyruvic acid. After complete dissolution SLOWLY add 5 mL MgCl _2_ stock solution and 500 μL CaCl _2_ stock solution. Bring to pH7.2-H7.4 with HCl. Sterile filter and store at 4 °C. The solution is stable for several months. Discharge if it becomes turbid. The addition of MgCl _2_ and CaCl _2_ is a critical step. If added too quickly, they precipitate making the solution cloudy. In this case, it must be discharged.

*1c. Culture medium:* 50% Basal medium Eagle (BME), 25% horse serum; 25% Hank's balanced salt solution (HBSS); 0.5% D-(+)-glucose; 0.5% L-glutamine (200 mM solution); 1% antibiotic antimycotic solution (100×).

To prepare 50 mL work under a laminar flow hood and use sterile glassware/plasticware. In a 100 mL cylinder add the components in the following order: 25 mL BME, 12.5 mL horse serum; 12.5 mL HBSS; 250 μL D-(+)-glucose; 250 μL L-glutamine; 500 μL antibiotic antimycotic solution. Transfer in a glass bottle and protect from light with aluminum foil. Store at 4 °C. Medium is stable for at least six months. Discharge if color changes and/or it becomes turbid.

*1d. Fixative*: Paraformaldehyde (PFA) 4% in 0.1 M phosphate buffer (PB), pH 7.4.

*1e.* *Buffer solution:* Phosphate-buffered saline (PBS) pH 7.4.

**Step 2:** Tissue sampling

Have ready the following: ice-cooled cutting solution; 50 mL sterile glass or plastic becker; 150 mm diameter sterile glass or plastic Petri dishes; sterile dissection/slice handling tools; sodium pentobarbital stock solution (room temperature); 500 μL disposable insulin syringes; sterile razor blades; sterile glass/disposable Pasteur pipettes; sterile filter paper dishes.

• Dissection of the brain and separation of individual slices after cutting (see Slice seeding below) should be carried out under sterile conditions as far as possible. If it is not possible to place the stereomicroscope under the laminar flow hood, dissection should be carried out under a simple plastic box opened in the front. The entire dissecting area should be cleaned and wiped off with 70% volume ethanol. During the production of slices, all procedures must be carried out in an ice-cold cutting solution. To keep the temperature a few degrees above 0 °C during the dissection, prepare some blocks of the frozen cutting solution to be added to the 4 °C chilled cutting solution contained in the Petri dish used to dissect the brain.
• Euthanize mice at the required post-natal age with an overdose of intraperitoneal sodium pentobarbital (60 mg/100 g body weight). Check for the absence of specific signs of life, *i.e.*, absence of withdrawal reflexes that normally disappear within 5 min of the pentobarbital injection. When the animal is dead cut the head with scissors and drop it into a small plastic box or a 50 mL beaker filled with ice-cooled cutting solution (about 2–4 °C). Wait a couple of minutes for the head to be cooled and at the same time washed from the blood.
• Transfer the head to a glass Petri dish (10 cm diameter or more) filled with the clean cutting solution at 2–4 °C. Quickly remove the brain from the skull while the head is kept submerged in the ice-cooled cutting solution. To do so use straight fine scissors: insert scissors laterally in the foramen magnum and cut the bone at the basis of the skull on both sides of the brain, use a scalpel to make a transversal cut at the level of the olfactory bulbs, and lift the calvarium. Scoop out the brain with a curved spatula to prevent damage.
• Before separating the cerebellum from the other parts of the brain, completely remove the meninges with a pair of N.7 Dupont forceps.
• Isolate the cerebellum under the stereomicroscope: use a razor blade to make a transversal cut at the level of the mesencephalon and to separate the cerebellum from cerebellar peduncles connecting it to the cerebral trunk.
• Place the cerebellum on the stage of the tissue chopper within a drop of the ice-cooled cutting solution. Operate the chopper and cut 350 μm-thick parasagittal slices. Once terminated slicing, collect slices with a curved spatula (they are usually stuck together) and place them in a sterile 50-mm Petri dish filled with the ice-cooled cutting solution. Store at 4 °C until ready to separate slices. Slices should be separated and plated as soon as possible. We have stored slices for at least 30 min before slicing with no obvious detrimental effects on survival. However, it could be possible to culture slices that have been stored for longer.
• Separate individual slices under the stereomicroscope with a spatula and a needle, trying not to damage the tissue. During the entire procedure, slices must be submerged in the ice-cooled cutting solution. Discharge the damaged slices and/or very small (lateral) slices. If cutting was done smoothly, at least 10-12 slices should be obtained from a P5-P7 cerebellum. If the cerebellum is not submerged by an excess of the cutting solution, cutting with the chopper is easier. Set section thickness to any value between 200 μm - 400 μm after wiping out the solution with a piece of filter paper. Other cutting parameters, such as blade force, must be adjusted based on the type of chopper in use. With the McIlwain tissue chopper in use, we set the blade force knob at ¾ of its rotation clockwise, and the speed control knob at ½ of its rotation clockwise.
• Use a spatula with curved edges to collect slices and transfer them from the cutting stage of the chopper to the Petri dish.

**Step 3** Slice seeding

• Before starting to seed slices onto the Millicell inserts bring the culture medium to room temperature and fill the required number of sterile 35-mm plastic Petri dishes with a 1.1 mL medium. Work under sterile conditions. The number of dishes required depends on the number of recovered slices, their size, and the experimental setup. In general, slices of the mouse post-natal cerebellum at day 5 have a maximum size of about 5 mm ^2^. Therefore, one can easily plate 5-6 slices (technical replicate when not co-culturing)/insert (experimental unit). Working with older animals or larger areas of the brain, *i.e.*, the cerebral cortex allows plating a maximum of (roughly) three slices/insert.
• If planning co-culture experiments, like those described here, remember to have all slices ready, *i.e.*, the L7-GFPreln ^(+/-)^F1/ slices and the L7-GFPreln ^(-/-)^F1/slices, before plating.
• Collect slices one by one and carefully lift them onto the dry Millicell membrane using a curved spatula. In co-culture experiments ( Figure 1) carefully mark the positions of individual slices so that it will be possible to easily recognize them during subsequent manipulations. See *Note 3.*
• Once the required number of slices has been plated in the insert, place it inside a 35-mm Petri dish filled with the medium as indicated at the beginning of this section. Be careful to avoid air bubbles forming between the insert membrane and the medium, *i.e.*, check that the membrane's lower surface is completely wet. Slices should be also wet but not submerged by the medium.
• Incubate at 34 °C in 5% volume CO _2_ for up to 30 days *in vitro* (DIV) – see *Note 4.* Cultures can be maintained *in vitro* even longer, if necessary. Medium has to be changed twice a week. Allow slices to equilibrate to the *in vitro* conditions for at least 4 DIV before follow-up or starting a pharmacological treatment (if applicable), because during this initial interval there is a massive phase of cell death, as a consequence of the cutting procedure, see Ref. 29.

*Notes*

1. Slices can also be prepared with an oscillating vibratome. This is often required for subsequent electrophysiological studies as the cutting procedure is less destructive than chopping. However, cutting with the chopper is easier and less time-consuming, which is advantageous if one has to plate many slices in the course of a single experiment.
2. Several media are available and the best medium must be chosen according to the experimenter’s needs. Table 1 below compares the solutions/media in our protocol with two protocols used by other authors that have been employed to also cultivate adult brain slices.
3. To recollect the slice positions in the insert it is advisable to mark a reference point in the insert border with a waterproof pen and to make a drawing of the insert and the slices seeded inside.
4. Slices obtained from the cerebellum (and other central nervous system (CNS) areas) survive better at temperatures below 37 °C, hence the temperature settings of the incubator are important for survival. However, it should be noted that the neuroprotective effect of mild hypothermia on cultured neurons may obscure the action of certain apoptotic inductors if one is interested in the study of cell death.

**Table 1.** Protocols for the preparation of brain slices.

Procedure/Solutions	This protocol	Ullrich *et al.* (2011) ^30^	Schommer *et al.* (2017) ^31^
Cutting solution	See text	No indication of a cutting solution	**To prepare 50 mL**: 40 mL Hibernate A 10 mL Horse Serum 0.5 mM L-Glutamine
Cutting	Chopper	Vibratome	Chopper
Growth medium	See text	50% MEM/HEPES 25% Horse Serum (inactivated) 25% Hank’s solution (HBSS) 2 mM NaHCO _3_ 2 mM L-glutamine pH 7.2	**To prepare 50 mL**: Horse Serum 8 mL 400 μL antibiotic/antimycotic solution 40 mL Neurobasal A
Treatment medium (Day 1)	N/A	N/A	**To prepare 50 mL**: Horse Serum 8 mL 400 μL antibiotic/antimycotic solution 40 mL Neurobasal A
Treatment medium (Following days)	N/A	N/A	**To prepare 50 mL**: B27 suppl. 800 μL 400 μL antibiotic/antimycotic solution 40 mL Neurobasal A

*Protocol for the characterization and validation of the model*

This protocol is advantageous to analyze cellular migration and dispersion in longitudinal studies.

Starting from biological images, it can be used to study cellular sociology, *i.e.*, to study the interactions of cells based on mathematical algorithms that rely on the analogies between cells and human societies. ^32^ It relies on a model of parametrization and quantitation of cellular population topographies developed by Marcelpoil and Usson (1992). ^26^

Software

- Voronoi Diagram Generator by Frederik Brasz
- ImageJ (RRID:SCR_003070) by NIH
- FIJI (RRID:SCR_002285) (Image J) by NIH
- Microsoft Windows 10 by Microsoft

**Step 1:** Generation of Voronoi diagrams (see *Note 1*)

**Figure 4.**
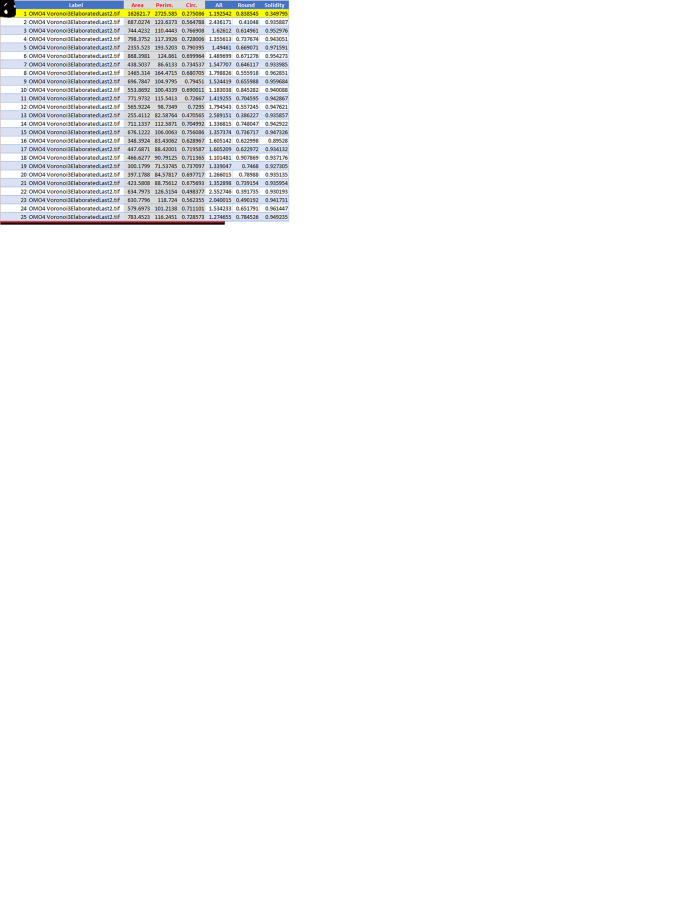
Elaboration of images for Voronoi analysis. The image of Figure 1 is taken as an example to show the individual steps of the technique. A: Generation of Voronoi polygons over the microscope image. Note that the center points (black dots) correspond to the cell centers; B: Color visualization of Voronoi polygons with center points; C: Color visualization of Voronoi polygons without center points; D: Elimination of the open polygons, *i.e.*, the polygons with one or more summits/sides outside the picture frame; E: Construction of the convex hull; F: Elimination of the polygons intersected by the convex hull. Note that the image in C (without center points) is used for this elaboration. This is because center points will be otherwise counted as particles by the ImageJ program in the subsequent elaboration; G: Elimination of the sides of the marginal polygons; H: Generation of the thresholded image to be elaborated by ImageJ with the Analyze Particles command; I: Generation of the overlay image with the indication of the number of each polygon analyzed by ImageJ. Note the number 1 circled in red at the center of the image. This number identifies the area in red in the following image; J: Image showing in red the area that ImageJ processes as a single particle. This area is discarded in the following elaborations; K: The values of Area, Perimeter, and Circularity (in red with gray background) of the first 25 particles (polygons) analyzed by ImageJ. In the example image processed here, ImageJ has analyzed a total of 318 particles of which particle #1 (highlighted in yellow) has to be discarded.

• Open the interactive Voronoi diagram (Thiessen polygon) generator. Figure 3 (left) shows the aspect of the generator mask.
• Upload the image to be analyzed (size must be 900×900 pixels and preferably saved as a PNG file). To do so your image has to be uploaded to the internet first ( *e.g.*, using Figshare or a personal website) so that it is possible to copy and paste its URL into the Voronoi generator. After uploading, the generator displays the image in its working space as shown in Figure 3 (right).
• Using the mouse, click above the center of each cell to generate the Voronoi polygons. Due to the thickness of the slice, it may be possible that two very close cells in the Z axis are not easily distinguished. This introduces an error that can be neglected considering that all slices are cut at the same initial thickness. In the end, you will obtain the image shown in Figure 4A. Save the image on your computer (right-click on the image and choose “save” from the drop-down menu).
• Choose “Visualization Normal” from the Visualization mode drop-down menu of the generator. The tessellation appears as shown in Figure 4B. Again, save the image on your computer (right-click on the image and choose “save” from the drop-down menu).
• Select “Hide sites” from the Options menu of the generator. The tessellation appears as shown in Figure 4C as the black dots corresponding to cell centers have disappeared. Again, save the image on your computer (right-click on the image and choose “save” from the drop-down menu).

**Step 2:** Elimination of the marginal polygons

Due to the properties of the Voronoi partition, some polygons of the paving are not statistically representative of the set of polygons. ^26^ Those polygons, referred to as the *marginal polygons* are associated with points located on the border of the cell population and have one or more summits that do not contain total information on their “surround”. Such summits are created by points that belong to a half-plane outside the image area. Therefore, every point of the cell population whose associated polygon satisfies one of the two following conditions must not be taken into account in the subsequent computations:

- The polygon is open (the central point belongs to the convex hull) - see Figure 4D.
- At least one of the summits of the polygon is outside the convex hull - see Figure 4E.

The convex hull of a set of N points, *i.e.*, the centers of the cells, is defined as the smallest convex set that contains all of the points. In the plane, this is a convex polygon.

• Elimination of the open polygons is carried out with Adobe Photoshop (RRID:SCR_014199) using the ***Magic wand tool*** to select and erase them from the image shown in Figure 4B. The result is shown in Figure 4D.
• Construct the convex hull from the image in Figure 4D. The convex hull is constructed with the ***Line tool*** by drawing segments that join the site points (cell centers) of the eliminated open polygons so that there are no concavities, as shown in Figure 4E.
• Using Photoshop, eliminate the polygons intersected by the convex hull and the polygons with open sides using the image of Figure 4C (without cell sites). The result is shown in Figure 4F.
• Cancel the sides of the marginal polygons. Use the ***Magic wand tool*** of Photoshop followed by the commands: ***Selection* → *Expand 2px***; ***Selection* → *Contract 1px***; ***Cancel***; ***Modify* → *Stroke*** ( ***color black***) ***2px***. You should obtain an image in which the area of the marginal polygons is empty as in Figure 4G. This is the last elaboration that will be used for the subsequent steps of analysis.

**Step 3:** Analysis of Voronoi polygons

• Open the image to be analyzed with ImageJ. Set the appropriate scale with ***Analyze* → *Set scale***.
• Run the following Macro by selecting ***Plugins* → *Macros* → *Run* → *Voronoi Macro*** ( Box 1).
• The macro enhances image contrast (optional – line 1), converts the image into a black and white (B&W) 8-bit image (line 2), finds the edges of the Voronoi polygons (line 3), and optimizes their contrast (lines 4-6) as shown in Figure 4H. It then sets up the measurements necessary for the following analysis of polygons: ***Area***, ***Shape descriptors***, and ***Perimeter*** (line 7). It also permits the creation of an image ( Figure 4I) with the overlay numerical indication of the individual polygons that the program has measured ( ***Add to overlay*** and ***Display label***). It also sets the number of Decimal places to 6 (line 7). Finally, the Macro performs the command Analyze Particles (line 8). Note the number 1 at the center of Figure 4I (encircled in red). This corresponds to the first counted particle that the program considers being the ensemble of the marginal polygons (highlighted in red in Figure 4J). Note that the red circle is only added here for clarity but not displayed at the end of the elaboration by ImageJ.
• At the end of the Macro, save all computed values in a .csv or a .xls file (according to the version of ImageJ used). This file must then be converted into a.xlsx Microsoft Excel file.

Box 1. **Voronoi Macro.**

run("Enhance Contrast…", "saturated=2");

run("8-bit");

run("Find Edges");

//run("Brightness/Contrast…");

setMinAndMax(0, 0);

run("Apply LUT");

run("Set Measurements…", "area perimeter shape limit display redirect=None decimal=6");

run("Analyze Particles…", "display summarize add in_situ");

**Step 4:** Analysis of data

• Open the .csv or .xls file generated by ImageJ with Microsoft Excel (RRID:SCR_016137). A table extracted from the file is shown in Figure 4K. It contains the following information: Column A: progressive numbering of the particles (polygons) counted by ImageJ; Column B: Identification of the image analyzed; Column C: Area (in μm ^2^ if the Set scale command has been set properly); Column D: Perimeter (in μm if the Set scale command has been set properly); Column E: Circularity (or Roundness factor); Columns F-H: Other shape descriptors computed by ImageJ that are not used in the analysis. Note that line 2 (highlighted in yellow) corresponding to Particle 1 must be deleted (as indicated above).
• Save the file as a.xlsx file.
• Open the.xlsx file in Microsoft Excel and calculate the following:
  ‐ **Mean of area**, **perimeter**, and **circularity** ( **roundness**)
  ‐ **Standard deviation of area**, **perimeter**, and **circularity** ( **roundness**)
  ‐ **Area Disorder** ( **AD**)
  ‐ **Roundness Factor Homogeneity** ( **RFH**)
  The **mean circularity** ( **roundness**) ( **RF**_**av**_) is computed directly by the ImageJ program using the following formula:
$${\text{RF}}_{\mathrm{av}}=\frac{1}{N}\sum_{i=1}^{N}\frac{4\mathrm{\pi A}\left(X_{i}\right)}{L{\left(X_{i}\right)}^{2}}$$

where A(X) is the area and L(X) is the perimeter of the N polygons generated by the Voronoi generator. RFav is a pure number (0 < RF _av_ ≤ 1).

The AD is calculated as follows:

$$\mathrm{AD}=1-{\left(1+\frac{\sigma_{A}}{A_{\mathrm{av}}}\right)}^{-1}$$

where σ _A_ is the area standard deviation, and A _av_ is the mean area.

The RFH is calculated as follows:

$$\mathrm{RFH}={\left(1+\frac{\sigma_{\mathrm{RF}}}{{\mathrm{RF}}_{\mathrm{av}}}\right)}^{-1}$$

where σ _RF_ is the roundness factor standard deviation, and RF _av_ is the mean roundness factor.

Both are pure numbers with values >0 and ≤1.

• Transfer the values of RF _av_, AD, and RFH to a new Microsoft Excel spreadsheet for subsequent statistical analysis.

*Notes*

1. It is possible to use several other Voronoi generators that can be found online as freeware or in dedicated programs. We found it particularly advantageous to use this generator because it is possible to directly upload the image to be analyzed and draw the sites, *i.e.*, the cell centers, straight on it. As an alternative, it is possible to upload the X-Y coordinates of the sites in this and other generators. To do so one can use the ImageJ program and the ***Multipoint tool*** to obtain the spatial coordinates to be then uploaded to the Voronoi generator.

## Results

### Histology

**Figure 5.**
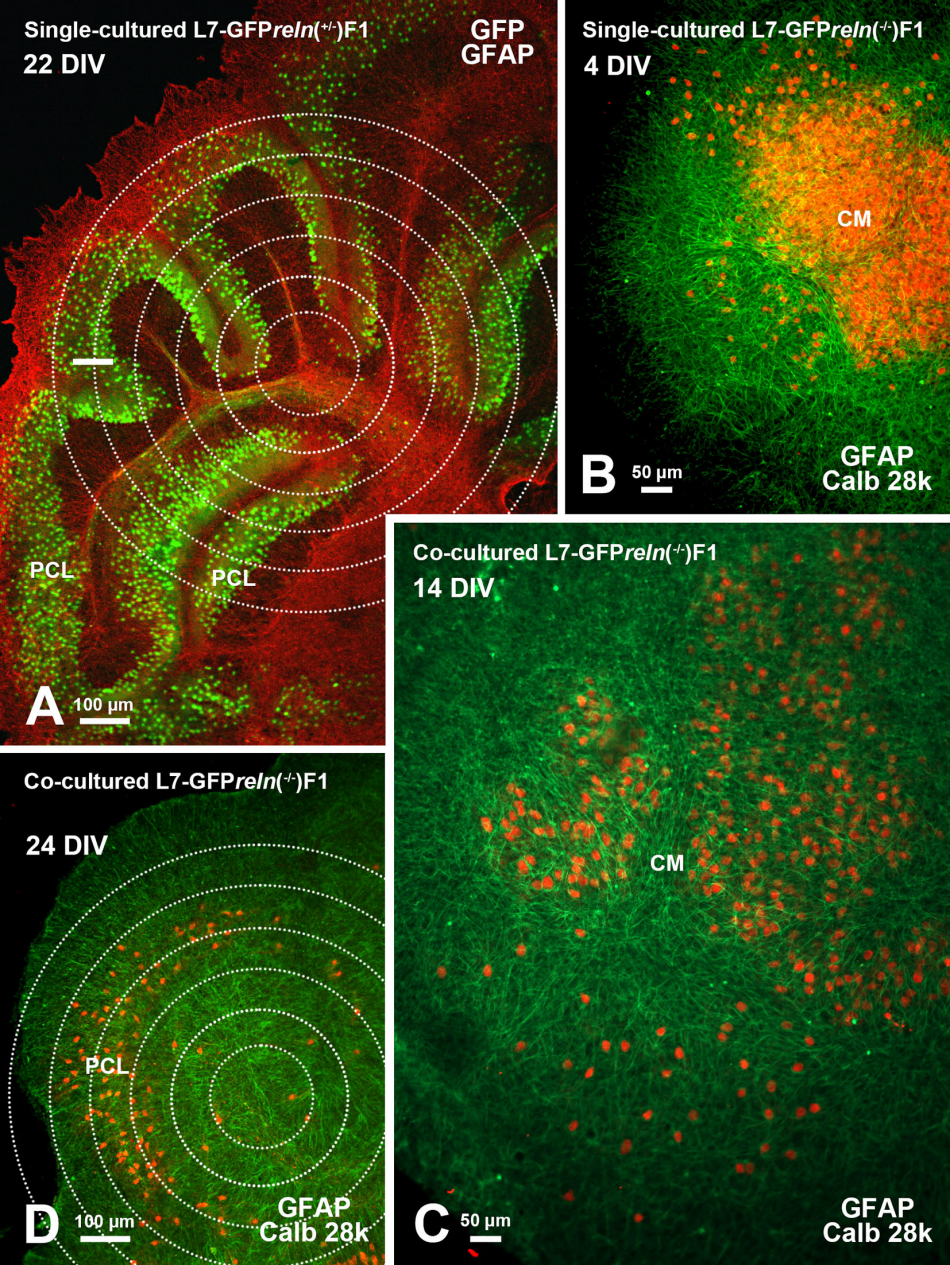
Histological aspects of single- and co-cultured slices after immunostaining with markers of PNs and glia. A: The cerebellar histology of a slice from an L7-GFPreln ^+/-^F1/ mouse shows a cortical stratification that is similar to that of an early postnatal wild-type mouse *in vivo.* The PNs are stratified to form a PCL composed of several layers of these neurons. B: A single-cultured slice from an L7-GFP *reln* ^-/-^F1/ mouse shows a large central mass of PNs. C-D: Exemplificative temporal evolution of a slice from an L7-GFP *reln* ^-/-^F1/mouse co-cultured with slices from L7-GFP *reln ^+/-^* F1/mice. The PNs are spread from the central mass (C) and try to form a multilayer PCL similar to that in A. Concentric circles in A and D are 100 μm spaced. Note that in B-D PNs have been stained for calb 28k and thus appear yellowish-orange for the superimposition of the green GFP signal and the red calb 28k fluorescence. *Abbreviations*: calb 28k = 28kD calbindin; CM = central mass; DIV = days *in vitro*; GFAP, Glial fibrillary acidic protein (red in A and green in B-D); GFP, green fluorescent protein; PCL = Purkinje cell layer; PNs = Purkinje neurons.

Organotypic cultures from L7-GFP *reln*F1/ ( Figure 1A) ^39^^–^^41^ permit a dynamic study of the effects of Reelin on neuronal migration and lamination of the cerebellar cortex. Thanks to GFP fluorescence, PNs can be visualized without the need for immunocytochemical labeling. In addition, our approach makes it unnecessary to use several groups of mice to be sacrificed at given postnatal ages to properly follow the cerebellar maturation. Figure 1B- D shows that, at the end of the experiments, cultures can be easily stained with two common markers of the cerebellar neurons. Figure 1D shows two co-cultured slices from a *reln* ^+/-^ (top right) and a *reln* ^-/-^ (bottom left). A comparison of the histology of the two slices permits clear identification of the phenotypical differences deriving from the different genetic backgrounds of the donor mice. Figure 2 shows the modifications over time in a single-cultured slice from an L7-GFP *reln* ^-/-^F1/mouse. After 29 days *in vitro,* the mass of the GFP fluorescent PNs tends to spread from the center of the slice but the neurons do not migrate to form a layered structure. Figure 5 shows that in co-cultures the histology of the slice derived from a homozygous ( *reln* ^-/-^) mouse ( Figure 5B- D) progressively changes to eventually become related to that from a heterozygous ( *reln* ^+/-^) mouse ( Figure 5A).

### Analysis of cellular sociology

**Figure 6.**
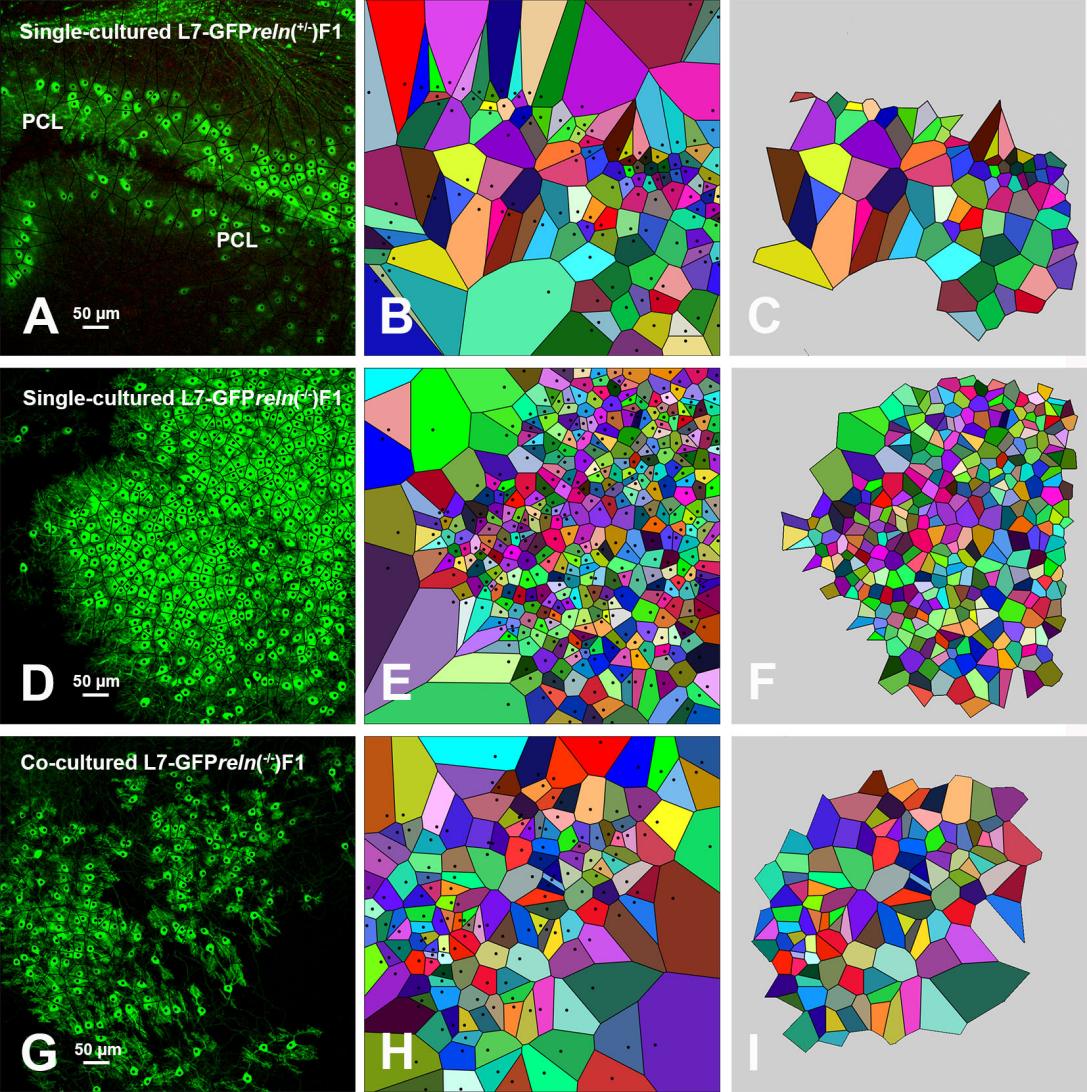
Voronoi tessellation of slices. Confocal images of the GFP-tagged PNs with superimposed Voronoi polygons (A, D, and G) that were generated and further elaborated as described in the Methods section and protocols.io. Images in B, E, and H show the initial elaboration of Voronoi polygons; those in C, F, and H show the last step of elaboration with the exclusion of the marginal polygons that are outside the convex hull. It can be seen that polygons are smaller and have a more homogeneous size in single cultured slices from L7-GFP *reln* ^-/-^F1/ mice (D-F), become larger and have less homogeneous sizes in co-cultured slices from L7-GFP *reln* ^-/-^F1/ mice (G-I), whereas slices from L7-GFPreln ^+/-^F1/mice display larger polygons of quite homogeneous sizes. Black dots in A-B, D-E, and G-H are the centers of the PNs. They have been cleared in the subsequent elaboration (C, F, and I) to avoid interference with automated counting. *Abbreviations*: GFP, green fluorescent protein; PCL = Purkinje cell layer; PNs = Purkinje neurons.

**Figure 7.**
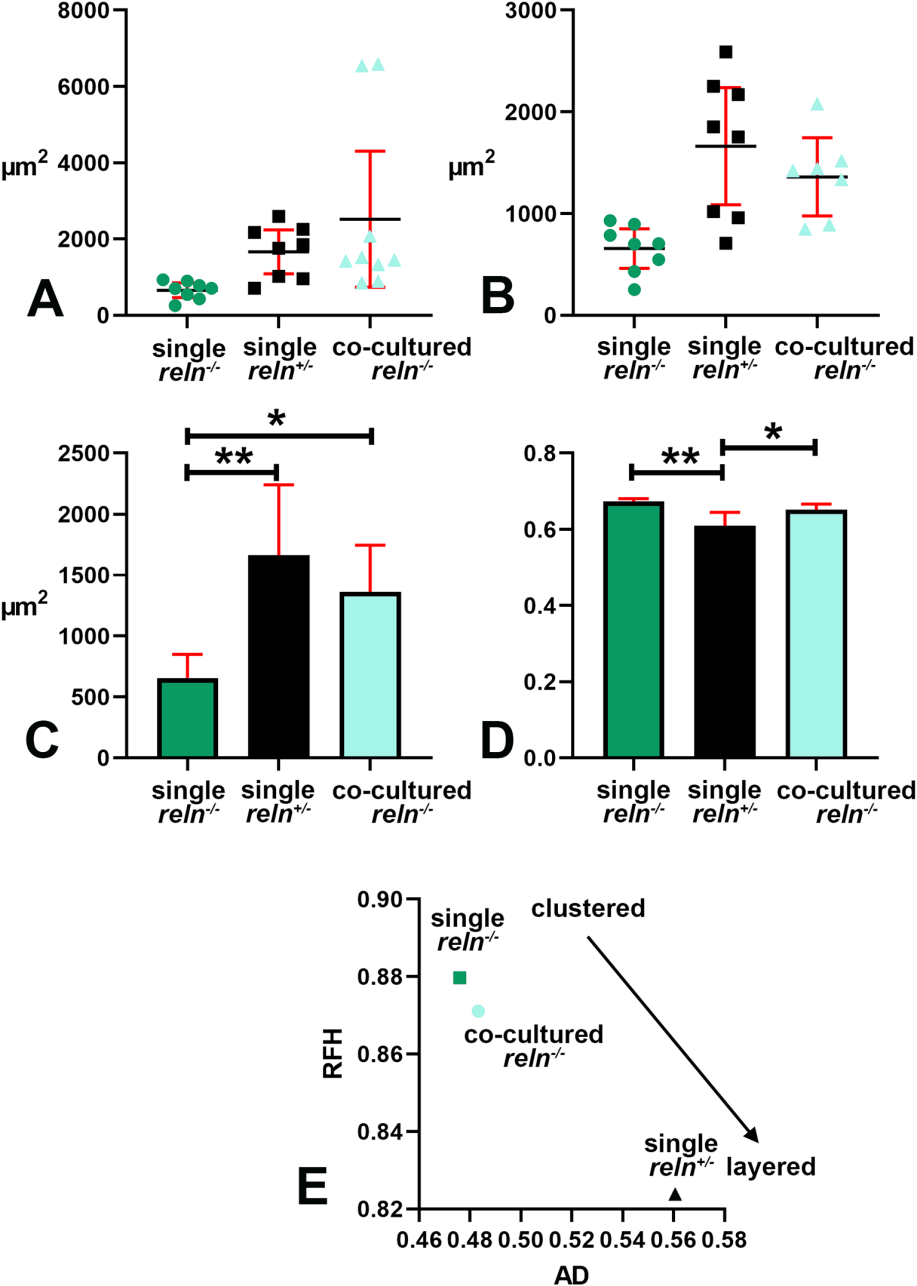
Quantitative analysis of Voronoi tessellation. A-B: Descriptive statistics of the mean areas of Voronoi polygons. Note that there is little variability in the mean areas of polygons among singularly cultured slices obtained from *reln* ^-/-^ homozygous mice as PNs remain aggregated into a central mass deep to the cerebellar cortex (see Figures 1, 2, 5); in the two other groups of cultures values are more dispersed and this indicates the dispersion of the PNs to form the PCL that will be typical of the mature cortex. A: Raw data plotted without any adjustment; B: Cleaned data after removing the two outliers identified in the co-cultured slices from *reln* ^-/-^ homozygous mice with the ROUT method (Q = 1%). Error bars are 95% confidence interval. Data passed the Kolmogorov-Smirnov normality test. C: Ordinary one-way ANOVA [F(2, 20) = 8.966; P value = 0.0017] followed by Tukey’s multiple comparison test shows that in co-cultured slices from *reln* ^-/-^ homozygous mice the mean polygon area is larger than in single-cultured slices from animals with the same genetic background (mean ± 95% CI: 1,361 ± 385 μm ^2^ *versus* 656 ± 195 μm ^2^, adjusted P value = 0.0287) and becomes closer to that of polygons in slices of *reln* ^+/-^ heterozygous mice (mean ± 95% CI: 1,361 ± 385 μm ^2^ *versus* 1,663 ± 576 μm ^2^, adjusted P value = 0.4678). This observation confirms quantitatively the dispersion of the PNs in co-cultured slices from *reln* ^-/-^ homozygous mice that lack Reelin but are exposed to the protein produced *ex vivo* by the slices from *reln* ^+/-^ heterozygous mice. Also note the difference in mean areas of Voronoi polygons in single cultures of slices explanted from homozygous *versus* heterozygous mice (mean ± 95% CI: 56 ± 195 μm ^2^ *versus* 1,663 ± 576 μm ^2^, adjusted P value = 0.0014). n (number of slices from five different mice) = 8; * 0.05≤ adjusted P value >0.01; ** 0.001≤ adjusted P value >0.001. D: Brown-Forsythe [F* (2, 10.38) =11.41, P value = 0.0024] and Welch [W (2, 11.78) =11.94, P value = 0.0015) ANOVA tests of RFav. Since Voronoi polygons are convex, the average type of spatial occupation of the PNs is well-characterized by the RFav (mean circularity). In slices from single-cultured *reln* ^-/-^ homozygous mice the RFav of the Voronoi polygons is higher than that of the polygons in slices from co-cultured slices of the same genetic background (mean ± 95% CI: 0.6733 ± 0.007 *versus* 0.6515 ± 0.0149, adjusted P value = 0.0314) and single-cultured *reln* ^+/-^ heterozygous mice (mean ± 95% CI: 0.6733 ± 0.007 *versus* 0.6091 ± 0.0298, adjusted P value = 0.0082). On the other hand, the difference in RFav between single cultured slices from heterozygous mice and co-cultured slices from *reln* ^-/-^ homozygous mice is not statistically significant (mean ± 95% CI: 0.6091 ± 0.0298 *versus* 0.6515 ± 0.0149, adjusted P value = 0.0718). The RF of a circle is 1 while that of a line is 0. Therefore, our analysis confirms mathematically that in single-cultured slices from *reln* ^+/-^ heterozygous mice and in co-cultures of slices from *reln* ^-/-^ homozygous mice there is a tendency to the alignment of the PNs, whereas in single-cultured slices from mice that lack Reelin the population of the PNs displays a spatial occupation consistent with the formation of a mass of cells in the cerebellar white matter. E: X-Y diagram showing topographical information of the PN population in the three experimental groups of cerebellar slices under the different culturing conditions reported in the Materials and Methods section. The X-axis displays the values of AD. AD varies when the value of the intrinsic disorder ( *i.e.*, the heterogeneity of the Voronoi polygon areas) increases, and a given value of AD corresponds to a given value of intrinsic disorder for any cell population. The Y-axis displays the values of RFH that varies in parallel to geometric disorder, *i.e.*, the homogeneity/inhomogeneity of the circularity of the Voronoi polygons. Both AD and RFH vary from 0 to 1. A highly ordered population is characterized by values of AD and RFH, respectively, corresponding to 0 and 1 ^26^, this means that all the polygons have the same area and circularity. The AD and RFH values are typical of a highly ordered population (high RFH, low AD) when one analyzes the clustered population of the PNs forming the central mass in the single-cultured *reln* ^-/-^ slices. When the PNs align to eventually form a well-defined layer in the slices from *reln* ^+/-^ heterozygous mice, the RFH diminishes and becomes closer to that of a line (=0), whereas the AD increases because the Voronoi polygons are small where the PNs tend to be aligned, but larger in the other parts of the slice (see Figure 4A- C). Note that in the co-cultured slices from *reln* ^-/-^ mice, there is a shift towards the coordinates of the *reln* ^+/-^ heterozygous mice. *Abbreviations*: PNs = Purkinje neurons; PCL = Purkinje cell layer; RF = roundness factor; RFav = mean roundness factor; AD = area disorder; RFH = roundness factor homogeneity.

Voronoi’s partition allowed for quantifying the dispersion of PNs in the presence or absence of Reelin. Starting from a set of points locating the position of the cell nuclei it was possible to obtain information on the order/disorder of the PN population ( Figure 6). Polygon areas in single-cultured slices from *reln* ^+/-^ animals ( Figure 6A- C and Figure 7A- C) are larger than those from *reln* ^-/-^ mice ( Figure 6D- F and Figure 7A- C). Conversely, in co-cultures polygon areas in *reln* ^-/-^ slices ( Figure 6G- I and Figure 7A- C) become larger than in single-cultured *reln* ^-/-^ slices, confirming a better dispersion of PNs in the presence of Reelin. RFav was not different in *reln* ^-/-^ slices under different culture conditions indicating that the geometry of the polygons was unchanged ( Figure 7D). We have also plotted AD and RFH in X/Y diagrams to show the spatial behavior of the PNs in the three groups of cultures ( Figure 7E) and established that in the co-cultures the Reelin provided by the *reln* ^+/-^ slices was sufficient to produce a measurable shift in the distribution of the PNs in *reln* ^-/-^ slices from the pattern observed when slices are cultivated singularly.

## Discussion

Here, not only we have been able to reduce the number of animals necessary to study *ex vivo* the effects of Reelin on cerebellar lamination, but also to avoid the use of the severe procedures that are necessary for *in vivo* longitudinal studies. Theoretically, our approach can be used for the study of the biological activities of any other brain-secreted protein, particularly if mutant and/or transgenic animals are available in which the protein under investigation is absent and thus it is possible to prepare co-cultures of the donor (normally expressing) and recipient (protein-lacking) slices. Several examples can be given to show the potential of our approach, a few of which are listed below. Remarkably, some of them refer to studies in *Drosophila*, a species that is easier to manipulate experimentally than mice and other mammals. For instance, the secreted neurotrophin Spätzle 3 was demonstrated to promote glial morphogenesis and neuronal survival and function in the fruitfly, ^33^ and, in rescue experiments, it had these effects only over very short distances. On the other hand, it was recently demonstrated that Slit, an evolutionarily conserved protein essential for brain development, acts at a long range and does not require processing by extracellular proteases in *Drosophila*. ^34^ Thus, our approach would be valuable to investigate the function of these two proteins in the mammalian brain, and, by plating slices at different distances, it could be useful to better establish their spatial range of action. Similarly, there is a growing interest in a better understanding of the function(s) of the Cyr61/CTGF/NOV (CCN) protein family in the nervous system. ^35^ CNN proteins bind directly to integrins and heparan sulfate proteoglycans and trigger multiple intracellular signaling pathways. At the cellular level, these proteins regulate gene expression and cell survival, proliferation, differentiation, senescence, adhesion, and migration, but little is known about their function in neural development. Likewise, co-cultures would be useful in studying the secretion of mutated huntingtin, which leads to neuronal degeneration in Huntington disease, ^36^ one of the most devastating neurodegenerative diseases. Our 3Rs approach could be primarily applied to neuroscience, although it is possible to envisage the preparation of slice cultures from organs such as the muscle, heart, liver, and solid tumors.

We have discussed in a previous publication the barriers for other potential end-users in the adoption of rodent *ex vivo* platforms in the field of neuroscience. ^17^ In short, the main disadvantage of organotypic cultures lies in the disconnection of the explants from other areas of the brain with interruption of afferent and/or efferent pathways. Potential solutions to address/overcome these problems should be mainly sought in the reconstruction *ex vivo* of these connections by co-cultivating areas that are physiologically connected *in vivo*. ^37^^,^^38^

We believe it is important that this or similar approaches are adopted by others, as they can be used in medium throughput screening experiments preliminary to (if necessary) true experimentation *in vivo.* They have several scientific benefits, ^29^ such as the possibility to precisely control the experimental environment, pharmacologically manipulating the system with ease, the relative facility to perform longitudinal studies, the possibility to use several complementary techniques (genetic engineering, electrophysiology, immunocytochemistry) for biological characterization.

As partly discussed elsewhere previously, ^17^ the potential of our approach in terms of animal reduction is remarkable as one can theoretically envisage reducing the number of experimental animals to at least one-fifth when aiming to characterize a single bioactive molecule.

## Conclusions

This 3Rs approach is useful to study the effect of secreted biomolecules in a system modeling the *in vivo* condition but with remarkable benefits for animal reduction and refinement, avoiding the use of heavy surgery that is often necessary for the molecule(s) to reach the brain.

## Data Availability

Figshare: Voronoi analysis - Cultured Reln haplodeficient heterozygous mouse cerebellar slices, https://doi.org/10.6084/m9.figshare.21063616. ^39^ This project contains the following underlying data:
- Original images and images elaborated for Voronoi analysis of cerebellar slices from postnatal day 5-7 *Reln* haplodeficient heterozygous hybrid mice (L7-GFPreln ^+/-^F1/) cultured for 21 days in vitro. - Area, Perimeter, and Circularity of individual Voronoi polygons. Area Disorder (AD), Roundness Factor Homogeneity (RFH), and Mean Roundness Factor (RFav) of the Voronoi polygon population. Figshare: Voronoi analysis - Cultured Reln deficient homozygous mouse cerebellar slices, https://doi.org/10.6084/m9.figshare.21063517. ^40^ This project contains the following underlying data:
- Original images and images elaborated for Voronoi analysis of cerebellar slices from postnatal day 5-7 *Reln* deficient homozygous hybrid mice (L7-GFPreln ^-/-^F1/) cultured for 21 days in vitro. - Area, Perimeter, and Circularity of individual Voronoi polygons. Area Disorder (AD), Roundness Factor Homogeneity (RFH), and Mean Roundness Factor (RFav) of the Voronoi polygon population. Figshare: Voronoi analysis - Reln deficient homozygous mouse cerebellar slices co-cultured with Reln haplodeficient heterozygous mouse cerebellar slices, https://doi.org/10.6084/m9.figshare.21063280. ^41^ This project contains the following underlying data:
- Original images and images elaborated for Voronoi analysis of cerebellar slices from postnatal day 5-7 Reln deficient homozygous hybrid mice (L7-GFPreln ^-/-^F1/) co-cultured for 21 days in vitro in the presence of slices from Reln haplodeficient homozygous hybrid mice (L7-GFPreln ^+/-^F1/). - Area, Perimeter, and Circularity of individual Voronoi polygons. Area Disorder (AD), Roundness Factor Homogeneity (RFH), and Mean Roundness Factor (RFav) of the Voronoi polygon population. Data are available under the terms of the Creative Commons Zero “No rights reserved” data waiver (CC0 1.0 Public domain dedication). Figshare: ARRIVE checklist for ‘Co-cultures of cerebellar slices from mice with different *reelin* genetic backgrounds as a model to study cortical lamination’, https://doi.org/10.6084/m9.figshare.21299211. ^42^ Data are available under the terms of the Creative Commons Zero “No rights reserved” data waiver (CC0 1.0 Public domain dedication).
