# Co-cultures of cerebellar slices from mice with different *reelin* genetic backgrounds as a model to study cortical lamination

**DOI:** 10.12688/f1000research.126787.2

**Published:** 2023-10-02

**Authors:** Adalberto Merighi, Laura Lossi

**Affiliations:** 1 Department of Veterinary Sciences, University of Turin, Grugliasco, 10095, Italy

**Keywords:** Reelin, Neuronal migration, Cerebellum, Purkinje neurons, Secreted proteins, Ex vivo methods, Cellular sociology, Voronoi tessellation, Spacial statistics

## Abstract

**Background:** Reelin has fundamental functions in the developing and mature brain. Its absence gives rise to the Reeler phenotype in mice, the first described cerebellar mutation. In homozygous mutants missing the Reelin gene ( *reln* ^-/-^), neurons are incapable of correctly positioning themselves in layered brain areas such as the cerebral and cerebellar cortices. We here demonstrate that by employing *ex vivo* cultured cerebellar slices one can reduce the number of animals and use a non-recovery procedure to analyze the effects of Reelin on the migration of Purkinje neurons (PNs).

**Methods:** We generated mouse hybrids (L7-GFP *reln*F1/) with green fluorescent protein (GFP)-tagged PNs, directly visible under fluorescence microscopy. We then cultured the slices obtained from mice with different *reln* genotypes and demonstrated that when the slices from *reln* ^-/-^ mutants were co-cultured with those from reln ^+/-^ mice, the Reelin produced by the latter induced migration of the PNs to partially rescue the normal layered cortical histology. We have confirmed this observation with Voronoi tessellation to analyze PN dispersion.

**Results:** In images of the co-cultured slices from *reln* ^-/-^ mice, Voronoi polygons were larger than in single-cultured slices of the same genetic background but smaller than those generated from slices of *reln* ^+/-^ animals. The mean roundness factor, area disorder, and roundness factor homogeneity were different when slices from *reln* ^-/-^ mice were cultivated singularly or co-cultivated, supporting mathematically the transition from the clustered organization of the PNs in the absence of Reelin to a layered structure when the protein is supplied *ex vivo.*

**Conclusions:** Neurobiologists are the primary target users of this 3Rs approach. They should adopt it for the possibility to study and manipulate *ex vivo* the activity of a brain-secreted or genetically engineered protein (scientific perspective), the potential reduction (up to 20%) of the animals used, and the total avoidance of severe surgery (3Rs perspective).

Research highlights **Scientific benefit(s):**

• Co-culturing slices from animals with different *reln* genetic backgrounds allows studying *ex vivo* the effects of Reelin in cerebellar cortical lamination
• Co-cultures can be pharmacologically manipulated and transfected with different types of fluorescent reporter proteins (FRP)
• They are amenable to electrophysiological recordings and immunocytochemical labeling

**3Rs benefit(s):**

• As several viable slices can be obtained from every single animal, these cultures substantially reduce the necessary number of mice in different experiments
• When a secreted molecule is to be studied (such as in the case of Reelin), this approach can be used to replace *in vivo* experiments where the substance has to be administered through more or less invasive routes, involving heavy surgery for molecules that are unable to pass the blood-brain barrier

**Practical benefit(s):**

• As experiments *in vivo* are more expensive than those in *ex vivo*/ *in vitro* conditions, slice co-cultures are highly valuable in terms of cost *vs.* effectiveness
• They allow mid-throughput screening of different culture conditions, *e.g.*, days *in vitro*, the chemical composition of the medium, *etc.*, offering the possibility to save time and plan fewer *in vivo* confirmatory experiments, if necessary
• They are less technically demanding than *in vivo* experiments

**Current applications:**

• Study of the effect of *Reln* dosage on the differentiation of the laminated structures of the brain, primarily the cerebral and cerebellar cortex

**Potential applications:**

• Co-cultures can be used for the study of cerebellar neuronal wiring *ex vivo*, *e.g.*, to reconstruct in a dish the olivo-cerebellar tract (climbing fibers) by cultivating together slices from cerebellum and medulla oblongata
• Co-cultures can be used to study the development and/or neurodegeneration in other areas of the brain and spinal cord

## Introduction

Like any other animal tissue, the nervous tissue is made up of cells and the surrounding extracellular matrix. Proteins that are released from the neural cells consist of those in the extracellular matrix itself, as well as extracellular signaling and adhesion molecules.

Compared to neurons and neural precursor cells, glial cells release a comparatively modest amount of proteins with a narrower range of functions, and according to a two-dimensional (2D) gels and liquid chromatography/mass spectrometry study, about 22% of the proteins secreted by neural cells intervene in cell-to-cell interactions. ^1^

Reeler was the first discovered mouse cerebellar mutation. ^2^ It was distinguished by typical gait changes ("reeling"), and thus thereafter named. In *reln* ^(-/-)^ recessive homozygous mutants, Reelin, a large secreted extracellular matrix glycoprotein, was completely absent and proved to be required for the normal development of layered brain structures ( *i.e.*, the cerebral and cerebellar cortices) being directly involved in neuronal migration. ^3^ Reelin absence causes severe cerebellar hypoplasia in *reln* ^(-/-)^ mice. This is because during the development of the cerebellar cortex, the granule cells synthesize and release the molecule into the neuropil, and Reelin acts as an attractant for correct migration and placement of the Purkinje neurons (PNs). ^3^ Remarkably, under the absence of Reelin, only 5% of the PNs align into their typical location between the mature molecular and granular layers of the cerebellar cortex, 10% remain in the internal granular layer, and those left behind are distributed throughout the white matter of the medullary body in a rather compact central mass. ^4^^–^^6^ Differently from *reln* ^(-/-)^ mice, heterozygous *reln* ^(+/-)^, and homozygous *reln* ^(+/+)^ animals, do not display obvious disturbances in cortical histology, although the size and number of the PNs, as well as their topology, may be somewhat altered also in the former. ^7^

More recently, it was demonstrated that not only Reelin is implicated in neuronal migration but, after development, it intervenes in synaptogenesis, neuronal plasticity, ^8^^–^^10^ and several neuropsychiatric disorders. ^11^^–^^13^ In addition, as Reelin is somehow the prototype of the brain extracellular matrix proteins because of its widely demonstrated intervention in the process of neural migration, there is a wide interest in gaining more information about its role in the normal and pathological brain. Finally, it seems reasonable to hold that the development of a reliable method to study the effects of Reelin on neuronal migration on live cells would be of benefit to the study of many other secreted brain proteins that regulate cell-to-cell interactions and their final spatial relations.

Several approaches are available for the study of these proteins. Among those *in vitro*, one can, for example, mention the above proteomic study, which was carried out on cortical neurons and astrocytes, as well as cell lines that were derived from dividing neural precursor cells of E16 rats. ^1^ Other approaches have used *in vivo* microdialysis combined with proteomics to discover new bioactive neuropeptides in the striatum ^14^ or biopanning, an affinity selection technique that selects for peptides binding to a given target to identify proteins of the extracellular matrix. ^15^ These and other more sophisticated secretome studies, for example, ^16^ are very important in the initial identification of individual proteins in specific neural cell populations but do not offer any cues about their function and are not suitable to be used in longitudinal studies aiming to understand the effects of a given protein over time.

Longitudinal studies *in vivo* that are necessary to follow Reelin (and other brain-secreted proteins) intervention at different time points of development or in adulthood require a high number of animals at different ages to lead to conclusive and biologically relevant results. We here report on an *ex vivo* procedure to study the effect of Reelin on neuronal migration. Our procedure is based on the use of organotypic co-cultures of the mouse postnatal cerebellum ^17^ but can be broadly employed in the study of the biological role of this (and other) secreted molecule in the brain. Alternatively, one could use three-dimensional (3D) cultures, but a reliable reconstruction of neural circuits is still very difficult to achieve and one should very well know these circuits *in vivo* such *e.g.*, as the case of the retina. ^18^ Moreover, the approach is usually expensive, and time-consuming, and cellular and biomolecular analysis is difficult to perform. ^19^

The 3Rs relevance of our approach is primarily related to 1. The reduction of the number of experimental animals; 2. The refinement of the procedures eluding the administration *in vivo* of molecules with (potential) toxic effects and the use of heavy brain surgery ( *e.g.*, the intraventricular administration of substances that are unable to cross the blood-brain barrier and/or the need to implant osmotic pumps for sustained administration over time).

Potential end-users are neurobiologists chiefly interested in brain development and neurodegeneration from a structural, functional, and pharmacological point of view. Neuromodulation, *i.e.*, the continuous change of synaptic network parameters, is required for adaptive neural circuit performance. This process is primarily based on the binding of a variety of secreted “modulatory” ligands to G protein-coupled receptors, which govern the operation of the ion channels affecting synaptic weights and membrane excitability. ^20^ The possibility to also apply our approach to studies on neuromodulation substantially widens the number of potentially interested researchers and opens yet unexplored avenues to implement the 3Rs principles.

The need for 3Rs research in these fields is supported by quantitative data. It is difficult to give an accurate estimate of the number of animals used for the purpose locally and worldwide. Yet one can reasonably hold that at least a 20% reduction (a figure based on the number of slices that can usually be generated per mouse) in their total number could be achieved by the adoption of this (and other) procedures *ex vivo*, as discussed in Ref. 17.

With specific regard to Reelin activity in normal and pathological conditions, a PubMed search (August 2023) with the string “reelin brain” gives back 1,560 results with a peak of 98 papers published in 2010 and a mean of 45 papers/year starting from 1993 (year of the first publication). A similar number of papers is retrieved from the Web of Science™ (1,578) and groups that have published at least two papers belong to 46 different countries. One has to consider that these figures increase substantially if the search is widened to secreted proteins more generally (i.e., 3,306 papers in PubMed for the string: secreted proteins AND “cell migration” AND brain). Although not often easy to glean from the Material and Methods section, in a typical publication *in vivo*, animal number ranges from 50 to 80 depending on the types of experiments, the number of experimental groups, and the approaches used.

The severity classification of our procedure as defined under Directive 2010/63/EU is non-recovery.

## Methods

### Materials and methods

*Mouse model*

Ethical statement

All experimental procedures described here have been approved by the Italian Ministry of Health (n. 65/2016-PR dated 21/01/2016 and n.1361.EXT.1 dated 27/12/2016) and the Bioethics Committees of the University of Turin and the Department of Veterinary Sciences (DSV). The number of animals (8 *reln* ^-/-^ and 8 *reln* ^+/-^) was kept to a minimum and all efforts were made to minimize their suffering.

Mouse housing and husbandry

Animals were housed in the facility of DSV under the following conditions: temperature 19–21 °C, humidity 55% ± 10%, light-dark cycle 12-12 h. Food (normal maintenance diet – meat-free rat and mouse diet SF00-100, Specialty Feeds, Glen Forrest Western Australia) and water (normal tap water) were given *ad libitum.* The bedding was a non-sterile woodchip. Environmental enrichment consisted of mini tubes, sizzle nests, and burrowing treats (Volkman Seed Small Animal Rodent Gourmet). Animals were bred in couples in a standard 484 cm ^2^ mouse cage. The mice themselves were not health-screened, the animal enclosure was free of the major rodent pathogens but some sentinels were positive for adventitious agents, *i.e.*, mouse hepatitis virus after indirect fluorescent antibody (IFA) test and Multiplexed Fluorometric ImmunoAssay (MFIA) and *Entamoeba* sp. after annual mouse health monitoring (HM) Federation of European Laboratory Animal Science Associations (FELASA) screen.

Generation of L7-GFP *reln*F1/mouse hybrids

Hybrids (L7-GFP *reln*F1/) were generated by crossing L7-green fluorescent protein (GFP) (RRID:IMSR_JAX:004690) female mice (L7GFP ^+/+^) with Reeler heterozygous ( *reln* ^+/-^) male mice (RRID:IMSR_JAX:000235). ^7^ L7GFP ^+/+^ mice express GFP under the control of the L7 promoter. ^21^^,^^22^ As the L7 gene is specifically expressed by the PNs, these neurons are tagged by GFP, allowing their visualization without the need for immunocytochemical labeling. Before use, all animals were genotyped by routine methods to ascertain their appropriate *reln* genetic background ^3^ and GFP expression (see *Note 1 and Supplemenatary Material 1* ^23^).

*Methods for the model development*

Preparation of organotypic single cultures and co-cultures from L7-GFP *reln*F1/of different genetic backgrounds

Experiments are reported in compliance with the ARRIVE guidelines, ^24^ including randomization of samples in culture inserts (slices in a single insert came from different mice), blinding of the experimenter who performed image analysis with unblinding at the end of image processing, and/or automation of quantification, as indicated in the following sections. Cultures were prepared from postnatal day 5 (P5) mice.

A step-to-step protocol for the preparation of cerebellar organotypic cultures (see also Figure S1-1 in *Supplementary Material 1* ^23^) has been deposited on protocols.io ( https://dx.doi.org/10.17504/protocols.io.6qpvr67bbvmk/v1). This protocol is a refinement of previously published procedures from our laboratory. ^25^^,^^26^

**Figure 1.**
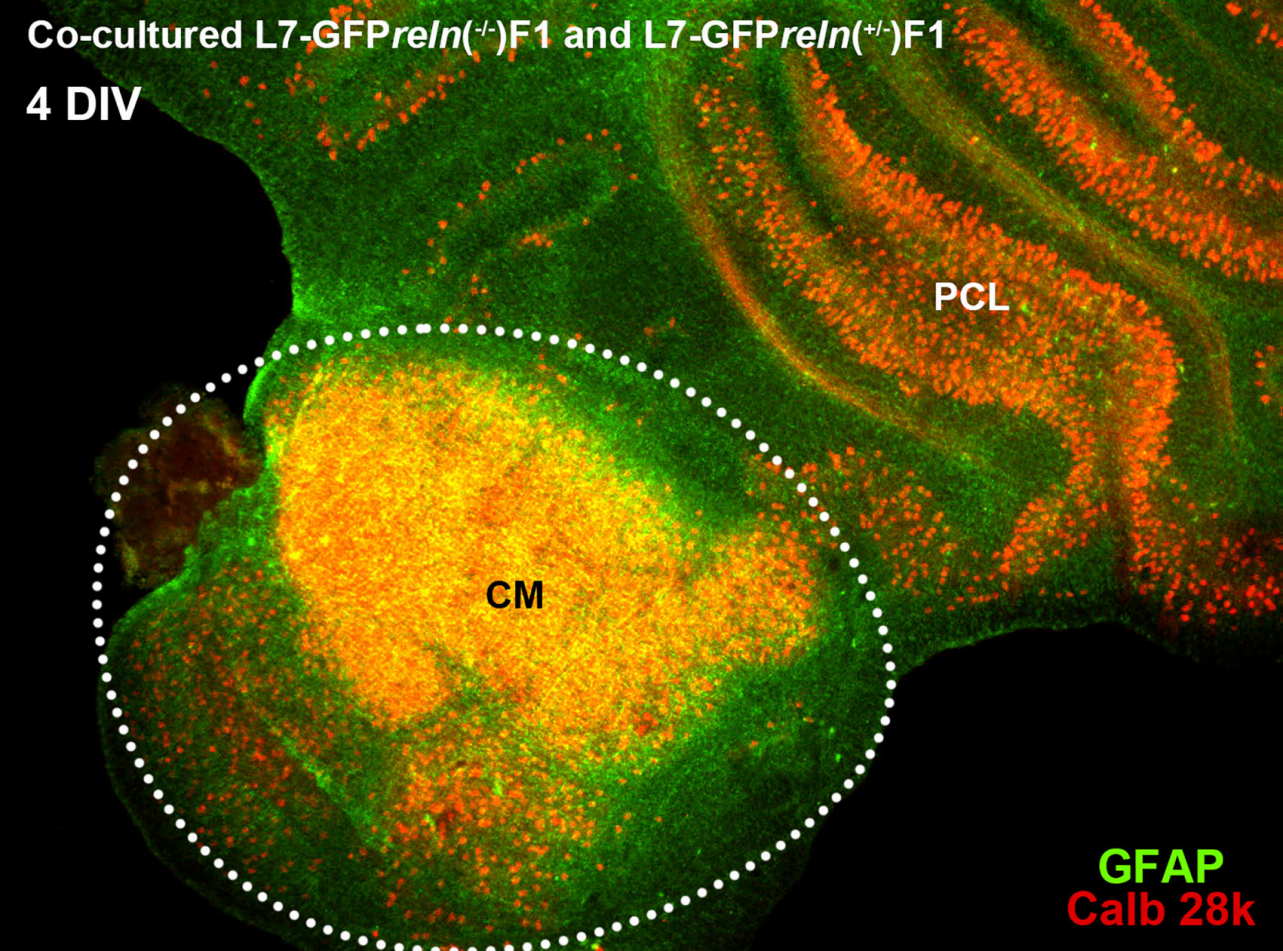
General features of cerebellar cultures. Histological aspects of two four-day *in vitro* (DIV) co-cultured slices explanted from mice with different *reln* genetic backgrounds. The slices were originally plated at a distance from each other but tend to expand with time *in vitro* and thus are in contact in this image. The slice from an L7-GFP *reln* ^(-/-)^ F1/mouse is marked by the dotted white line. Note the mass of PNs at the center of the slice (CM). The other slice was obtained from an L7-GFP *reln* ^(+/-)^ F1/mouse. Note that PNs are stratified in an attempt to form a discrete layer. PNs have been stained for calb 28k and thus appear yellowish-orange for the superimposition of the green GFP signal and the red calb 28k fluorescence. *Abbreviations*: calb 28k = 28kD calbindin; CM = central mass; DIV = days *in vitro*; GFP = green fluorescent protein; PCL = Purkinje cell layer.

In the co-culture protocol, slices from L7-GFP *reln* ^(+/-)^ F1/and L7-GFP *reln* ^(-/-)^ F1/mice were plated together ( Figure 1). The positions of each genotypically identified slice in the insert were recorded so that they could be identified and monitored for the entire duration of the experiments. Slices in each insert were numbered in a clockwise direction starting from a point indicated by a permanent mark on the side of the plastic insert. In the following analysis, the experimenter remained unaware of the matching between the slice number and the genotype of the donor mouse (see *Supplementary Material 1* ^23^ and *Note 2*).

Qualitative analysis of PN migration

**Figure 2.**
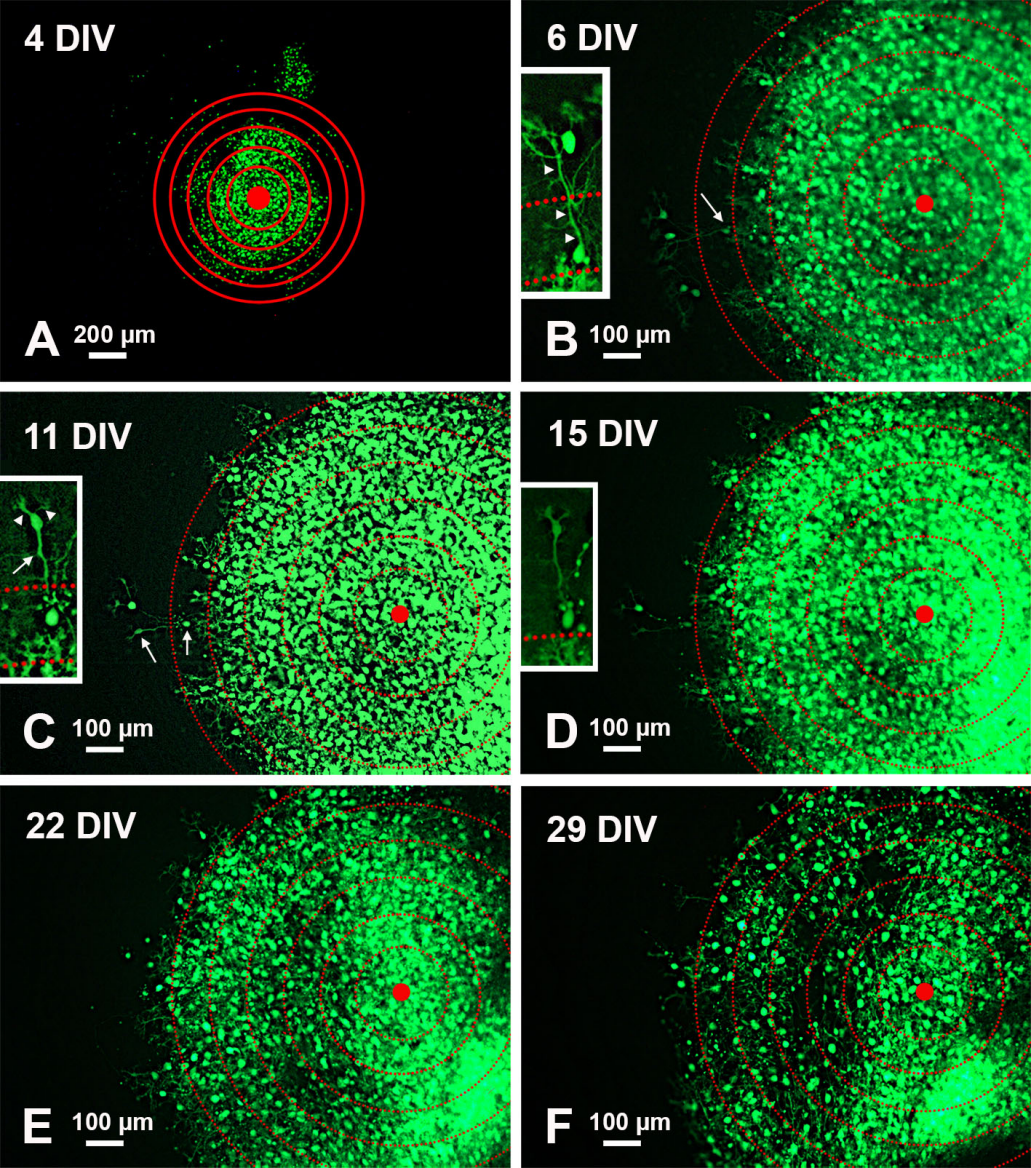
Temporal modifications of a single-cultured slice from an L7-GFP *reln* ^-/-^F1/mouse. A: Low magnification view of the slice with superimposed concentric circles surrounding its center. B-F: Higher magnification of the same slice and its evolution over time. The apparent dispersion of the PNs is mainly due to the death of individual cells. The inserts in B-D show the histological features of two PNs at the periphery of the central mass. Note the reduction of fluorescence in D and the disappearance of the two cells in E-F. *Arrows* in the main panels point to the PNs shown in the inserts at higher magnification. Arrows in the inserts indicate the PN axon, and arrowheads the main dendrites. *Abbreviations*: DIV = days *in vitro*; GFP = green fluorescent protein; PNs = Purkinje neurons.

To analyze qualitatively PN migration in individual slices, organotypic cultures were photographed under a transmitted fluorescence light microscope at different time intervals. A series of six concentric circles spaced by 100 μm was superimposed on each photograph and roughly centered to the geometric center of the slice ( Figure 2).

Immunocytochemistry

A step-to-step protocol for the immunofluorescence staining of organotypic cultures can be found in Ref. 25. In the main text and *Supplementary material 1* ^23^ (Figure S1-2), we simply show exemplificative staining with a rabbit anti-glial fibrillary acidic protein (GFAP – astrocytic marker) polyclonal antibody (Abcam Cat# ab7260, RRID: AB_305808), a mouse anti-28kD calbindin (a marker of the PNs) monoclonal antibody (Abcam Cat# ab9481, RRID: AB_2811302), and three markers of differentiating granule cells: mouse anti-paired-box protein PAX6 (PAX6) monoclonal antibody (Santa Cruz Biotechnology Cat# sc-81649, RRID: AB_1127044), rabbit anti-neuronal differentiation 1 (NEUROD1) monoclonal antibody (Abcam Cat# 3181-1, RRID: AB_2251162), and rabbit anti-Zic Family Member 2 (ZIC2) polyclonal antibody (Antibodies-Online Cat# ABIN129629, RRID: AB_10784806). We also stained some slices with the DNA-synthesis marker 5-bromo-2′-deoxyuridine (BrdU) with a mouse monoclonal antibody (BD Biosciences Cat# 556028, RRID: AB_396304). All antibodies were used at dilutions ranging from 1:100 to 1:200.

Microscopy and photography

Cultures were photographed directly under a 10× or 20× objective of a Leika DM 6000 transmitted light microscope taking care not to expose them to environmental contaminants. Alternatively, they have been maintained in a microscope stage incubator fitted to a Leika SP5 Laser confocal microscope and photographed with a 20× lens (see *Note 2*).

*Notes*

1. Genotyping was done by sampling a small piece of the pinna so that at the same time it was possible to identify the subject and extract the genomic DNA.
2. Although not strictly necessary, one can use an incubator that is fitted to the microscope stage (see *e.g.*, Figures 2 and 3 in Ref. 27) to longitudinally monitor cultures and easily take photographs of the same slice so that individual microscopic fields can be easily recognized. Although this is ideal when it is necessary to pharmacologically challenge the cultures over time, it may be unpractical when several cultures must be processed together such is the case of the co-culture protocol here described.

*Methods for the characterization and validation of the model*

We used two different methods to analyze cell dispersion quantitatively aiming at demonstrating the precise relationship between cultural conditions and the spatial distribution of the PNs. First, we employed Voronoi tessellation, ^28^ as this cellular sociology approach has proved useful in other biological contexts. ^29^ In addition, we used a Geographic Information Systems (GIS)-based method to statistically analyze the 2D data distribution of the PNs spatially. GIS was originally developed for cartographic studies but is more and more widely employed in the biomedical field at different levels of complexity, from cells to tissues, organs, and entire populations. ^30^

The numbers of technical repeats (individual slices from a single cerebellum) and independent biological repeats (organotypic cultures/co-cultures made by adding 3–6 individual slices to a single culture dish) are indicated in figure legends.

Cultures were obtained from two groups of mice: L7-GFP *reln* ^(+/-)^F1/ (n = 5) and L7-GFP *reln* ^(-/-)^F1 (n = 5). Cerebellar slices from these animals were subdivided into three groups ( *Supplementary Material 1* ^23^):

1) single cultured L7-GFP *reln* ^(+/-)^F1/ slices, 2) single cultured L7-GFP *reln* ^(-/-)^F1/ slices, and 3) co-cultured L7-GFP *reln* ^(+/-)^F1/ slices + L7-GFP *reln* ^(-/-)^F1/ slices. In co-cultures, the ratio of L7-GFP *reln* ^(+/-)^F1/to L7-GFP *reln* ^(-/-)^F1/slices was 2:1/3:1. At least eight slices from each group were used for the analysis of cellular sociology (see below). The sample size (number of slices) was calculated using the G*Power calculator 3.1.9.4. ^31^ Input parameters were (unpaired t-test power calculator): tails, two; parent distribution, Normal; α error probability, 0.05; power (1-β error probability), 0.95; effect size d, 2. The effect size was considered high based on qualitative observations and considering the mean area of the Voronoi polygons as the primary outcome. Output parameters were: non-centrality r δ, 3.9088201; critical t, 2.1557656; Df, 13.2788745; sample size group 1, 8; sample size group 2, 8; Total sample size, 16; actual power, 0.9508778.

The eight slices/group of mice were randomly selected after sectioning the cerebella of five different animals at least.

The detailed procedure for the calculation of Voronoi diagrams has been deposited on protocols.io ( https://dx.doi.org/10.17504/protocols.io.yxmvmnrx6g3p/v1). The parameters extracted from the analysis of Voronoi polygons (forms) were: the mean area, the average roundness factor (RFav), the roundness factor homogeneity (RFH), and the area heterogeneity, referred to as area disorder (AD). These parameters are characteristic of the population topography ^28^ and were used for subsequent statistical analysis.

The detailed procedures for the spatial analysis with GIS have also been deposited on protocols.io ( https://dx.doi.org/10.17504/protocols.io.ewov1o697lr2/v2). With GIS Measuring_Geographic_Distribution, Analyzing_Patterns, and Mapping_Clusters tools we have statistically compared the 2D distribution of the PNs among the three experimental groups of slices.

### Statistics

We have used GraphPad Prism (RRID:SCR_002798) version 9.0.2 for Windows, GraphPad Software, San Diego, California USA, to assess the variations in the mean areas of Voronoi polygons, and RFav using a 95% confidence interval. Data were checked for outliers with the ROUT method (Q = 1) and normality with the Kolmogorov-Smirnov test. Further details are given in figure legends. Inferential statistics were performed using ordinary one-way ANOVA followed by Tukey’s multiple comparison tests when data had a Gaussian distribution. The Brown-Forsythe test for equality of means and the Welch test was used for data sampled from populations with different variances.

Spatial statistics were calculated with ArcGIS for Desktop Basic (RRID: SCR_011081).

### Protocols

*Protocol for establishing the co-culture model*

The protocol below describes the step-by-step procedure required to establish and validate the long-term co-cultures of the postnatal murine cerebellum. With minimal modifications, it could be adapted to co-cultures of other areas of the brain, *e.g.*, the hippocampus and entorhinal cortex or the cerebellum and the medulla oblongata containing the caudal (inferior) olivary nucleus. It stemmed from the single culture protocol previously developed in our laboratory. ^25^

Equipment

• Surgical instruments for brain dissection: universal scissors (length 13 cm), fine scissors straight and curved, Adson forceps, student anatomical standard pattern forceps, Dumont #7 forceps, gross anatomy blade (#20) and handle (#4), straight and curved spatulas, razor blades
• Dissecting microscope, *e.g.*, Stereo microscope EZ4, Leica 10447197
• CO _2_ incubator, *e.g.*, Certomat CS-18 Sartorius BBI-8863385
• McIlwain tissue chopper with Petri dish modification Campden Instruments Model TC752-PD – see *Note 1.*
• Millicell-CM ^®^ Cell Culture Inserts, 30 mm, hydrophilic PTFE, 0.4 μm, Merck, PICM0RG50
• Sterile 35-mm Petri dishes
• Nalgene ^®^ vacuum filtration system, filter capacity 1000 mL, pore size 0.2 μm, Sigma-Aldrich, Z358207
• 500 μL disposable insulin syringes
• Sterile glass/disposable Pasteur pipettes
• Sterile filter paper dishes

Chemicals

• Pentobarbital sodium, Sigma-Aldrich, Y00021941
• D-(+)-Glucose, Sigma-Aldrich, G8270
• L-Ascorbic acid, Sigma-Aldrich, A92902
• Pyruvic acid, Sigma-Aldrich, 107360
• N-Methyl-D-glucamine, Sigma-Aldrich, M2004
• Sodium bicarbonate, Sigma-Aldrich, S5761
• Potassium chloride, Sigma-Aldrich, P3911
• Sodium phosphate monobasic, Sigma-Aldrich, S0751
• Calcium chloride, Sigma-Aldrich, C1016
• Magnesium chloride, Sigma-Aldrich, M8266
• Basal Medium Eagle, Sigma-Aldrich, B9638
• Horse serum, Sigma-Aldrich, H1138
• Hanks’ Balanced Salt solution, Sigma-Aldrich Catalog, H6648
• L-Glutamine solution, Sigma-Aldrich Catalog, G7513
• Antibiotic Antimycotic Solution (100×) Stabilized, Sigma-Aldrich, A5955
• Paraformaldehyde, powder, 95%, Sigma-Aldrich, 158127

**Step 1:** Preparation of solutions and culture medium (see *Note 2*)

*1a. Stock solutions*: 1 M CaCl _2_; 1 M MgCl _2_; 5% volume pentobarbital sodium in ddH _2_O.

*1b. Cutting solution:* 130 mM n-methyl-D-glucamine Cl (NMDG); 24 mM NaHCO _3_; 3.5 mM KCl; 1.25 mM NaH _2_PO _4_; 0.5 mM CaCl _2_; 5 mM MgCl _2_; 10 mM D-(+)-glucose; 1 mg/mL ascorbic acid; 2 mg/mL pyruvic acid.

To make 1 L, pour 850 mL of double-distilled water into a volumetric flask. Add 25.38 g NMDG, 2.017 g NaHCO _3_, 261 mg KCl, 172 mg NaH _2_PO _4_, 1.80 g D-(+)-glucose, 1 g ascorbic acid, 2 g pyruvic acid. After complete dissolution SLOWLY add 5 mL MgCl _2_ stock solution and 500 μL CaCl _2_ stock solution. Bring to pH 7.2-H7.4 with HCl. Sterile filter and store at 4 °C. The solution is stable for several months. Discharge if it becomes turbid. The addition of MgCl _2_ and CaCl _2_ is a critical step. If added too quickly, they precipitate making the solution cloudy. In this case, it must be discharged.

*1c. Culture medium:* 50% Basal medium Eagle (BME), 25% horse serum; 25% Hank’s balanced salt solution (HBSS); 0.5% D-(+)-glucose; 0.5% L-glutamine (200 mM solution); 1% antibiotic antimycotic solution (100×).

To prepare 50 mL work under a laminar flow hood and use sterile glassware/plasticware. In a 100 mL cylinder add the components in the following order: 25 mL BME, 12.5 mL horse serum; 12.5 mL HBSS; 250 μL D-(+)-glucose; 250 μL L-glutamine; 500 μL antibiotic antimycotic solution. Transfer to a glass bottle and protect from light with aluminum foil. Store at 4 °C. Medium is stable for at least six months. Discharge if color changes and/or it becomes turbid.

*1d. Fixative*: Paraformaldehyde (PFA) 4% in 0.1 M phosphate buffer (PB), pH 7.4.

*1e.* *Buffer solution:* Phosphate-buffered saline (PBS) pH 7.4.

**Step 2:** Tissue sampling

Have ready the following: ice-cooled cutting solution; 50 mL sterile glass or plastic becker; 150 mm diameter sterile glass or plastic Petri dishes; sterile dissection/slice handling tools; sodium pentobarbital stock solution (room temperature); 500 μL disposable insulin syringes; sterile razor blades; sterile glass/disposable Pasteur pipettes; sterile filter paper dishes.

• Dissection of the brain and separation of individual slices after cutting (see Slice seeding below) should be carried out under sterile conditions as far as possible. If it is not possible to place the stereomicroscope under the laminar flow hood, dissection should be carried out under a simple plastic box opened in the front. The entire dissecting area should be cleaned and wiped off with 70% volume ethanol. During the production of slices, all procedures must be carried out in an ice-cold cutting solution. To keep the temperature a few degrees above 0 °C during the dissection, prepare some blocks of the frozen cutting solution to be added to the 4 °C chilled cutting solution contained in the Petri dish used to dissect the brain.
• Euthanize mice at the required post-natal age with an overdose of intraperitoneal sodium pentobarbital (60 mg/100 g body weight). Here we have used postnatal day 5 (P5) mice based on the known data on PNs’ migration in the mouse cerebellum. Check for the absence of specific signs of life, *i.e.*, the absence of withdrawal reflexes that normally disappear within 5 min of the pentobarbital injection. When the animal is dead cut the head with scissors and drop it into a small plastic box, or a 50 mL beaker filled with ice-cooled cutting solution (about 2–4 °C). Wait a couple of minutes for the head to be cooled and at the same time washed from the blood.
• Transfer the head to a glass Petri dish (10 cm diameter or more) filled with the clean cutting solution at 2–4 °C. Quickly remove the brain from the skull while the head is kept submerged in the ice-cooled cutting solution. To do so use straight fine scissors: insert scissors laterally in the foramen magnum and cut the bone at the basis of the skull on both sides of the brain, use a scalpel to make a transversal cut at the level of the olfactory bulbs, and lift the calvarium. Scoop out the brain with a curved spatula to prevent damage.
• Before separating the cerebellum from the other parts of the brain, completely remove the meninges with a pair of N.7 Dupont forceps.
• Isolate the cerebellum under the stereomicroscope: use a razor blade to make a transversal cut at the level of the mesencephalon and to separate the cerebellum from cerebellar peduncles connecting it to the cerebral trunk.
• Place the cerebellum on the stage of the tissue chopper within a drop of the ice-cooled cutting solution. Operate the chopper and cut 350 μm-thick parasagittal slices. Once terminated slicing, collect slices with a curved spatula (they are usually stuck together) and place them in a sterile 50-mm Petri dish filled with the ice-cooled cutting solution. Store at 4 °C until ready to separate slices. Slices should be separated and plated as soon as possible. We have stored slices for at least 30 min before slicing with no obvious detrimental effects on survival. However, it could be possible to culture slices that have been stored for longer.
• If the cerebellum is not submerged by an excess of the cutting solution, cutting with the chopper is easier. Set section thickness to any value between 200 μm - 400 μm after wiping out the solution with a piece of filter paper. Other cutting parameters, such as blade force, must be adjusted based on the type of chopper in use. With the McIlwain tissue chopper, we set the blade force knob at ¾ of its rotation clockwise, and the speed control knob at ½ of its rotation clockwise.
• Use a spatula with curved edges to collect slices and transfer them from the cutting stage of the chopper to the Petri dish. Separate individual slices under the stereomicroscope with a spatula and a needle, trying not to damage the tissue. During the entire procedure, slices must be submerged in the ice-cooled cutting solution. Discharge the damaged slices and/or very small (lateral) slices. If cutting was done smoothly, at least 10–12 slices should be obtained from a P5-P7 cerebellum.

**Step 3:** Slice seeding

• Before starting to seed slices onto the Millicell inserts bring the culture medium to room temperature and fill the required number of sterile 35-mm plastic Petri dishes with a 1.1 mL medium. Work under sterile conditions. The number of dishes required depends on the number of recovered slices, their size, and the experimental setup. In general, slices of the mouse post-natal cerebellum at day 5 have a maximum size of about 5 mm ^2^. Therefore, one can easily plate 5-6 slices (technical replicate when not co-culturing)/insert (experimental unit). Working with older animals or larger areas of the brain, *i.e.*, the cerebral cortex allows plating a maximum of (roughly) three slices/insert.
• If planning co-culture experiments, like those described here, remember to have all slices ready, *i.e.*, the L7-GFP *reln* ^(+/-)^F1/ slices and the L7-GFP *reln* ^(-/-)^F1 /slices, before plating.
• Collect slices one by one and carefully lift them onto the dry Millicell membrane using a curved spatula. In co-culture experiments ( Figure 1) carefully mark the positions of individual slices so that it will be possible to easily recognize them during subsequent manipulations. See *Supplementary Material 1* ^23^ and *Note 3.*
• Once the required number of slices has been plated in the insert, place it inside a 35-mm Petri dish filled with the medium as indicated at the beginning of this section. Be careful to avoid air bubbles forming between the insert membrane and the medium, *i.e.*, check that the membrane’s lower surface is completely wet. Slices should be also wet but not submerged by the medium.
• Incubate at 34 °C in 5% volume CO _2_ for up to 30 days *in vitro* (DIV) – see *Note 4.* Cultures can be maintained *in vitro* even longer, if necessary. The medium has to be changed twice a week. Allow slices to equilibrate to the *in vitro* conditions for at least 4 DIV before follow-up or starting a pharmacological treatment (if applicable), because during this initial interval there is a massive phase of cell death, as a consequence of the cutting procedure, see Ref. 32.

*Notes*

1. Slices can also be prepared with an oscillating vibratome. This is often required for subsequent electrophysiological studies as the cutting procedure is less destructive than chopping. However, cutting with the chopper is easier and less time-consuming, which is advantageous if one has to plate many slices in the course of a single experiment.
2. Several media are available and the best medium must be chosen according to the experimenter’s needs. Table 1 below compares the solutions/media in our protocol with two protocols used by other authors that have been employed to cultivate adult brain slices.
3. To recollect the slice positions in the insert it is advisable to mark a reference point in the insert border with a waterproof pen and to make a drawing of the insert and the slices seeded inside (see *Supplementary Material 1* ^23^).
4. Slices obtained from the cerebellum (and other central nervous system (CNS) areas) survive better at temperatures below 37 °C, hence the temperature settings of the incubator are important for survival. However, it should be noted that the neuroprotective effect of mild hypothermia on cultured neurons may obscure the action of certain apoptotic inductors if one is interested in the study of cell death.

**Table 1.** Protocols for the preparation of brain slices.

Procedure/Solutions	This protocol	Ullrich *et al.* (2011) ^33^	Schommer *et al.* (2017) ^34^
Cutting solution	See text	No indication of a cutting solution	**To prepare 50 mL**: 40 mL Hibernate A 10 mL Horse Serum 0.5 mM L-Glutamine
Cutting	Chopper	Vibratome	Chopper
Growth medium	See text	50% MEM/HEPES 25% Horse Serum (inactivated) 25% Hank’s solution (HBSS) 2 mM NaHCO _3_ 2 mM L-glutamine pH 7.2	**To prepare 50 mL**: Horse Serum 8 mL 400 μL antibiotic/antimycotic solution 40 mL Neurobasal A
Treatment medium (Day 1)	N/A	N/A	**To prepare 50 mL**: Horse Serum 8 mL 400 μL antibiotic/antimycotic solution 40 mL Neurobasal A
Treatment medium (Following days)	N/A	N/A	**To prepare 50 mL**: B27 suppl. 800 μL 400 μL antibiotic/antimycotic solution 40 mL Neurobasal A

*Protocol for the characterization and validation of the model*

Protocol 1: Voronoi’s Tessalation

This protocol is advantageous for analyzing cellular migration and dispersion in longitudinal studies. Starting from biological images, it can be used to study cellular sociology, *i.e.*, to study the interactions of cells based on mathematical algorithms that rely on the analogies between cells and human societies. ^35^ It relies on a model of parametrization and quantitation of cellular population topographies developed by Marcelpoil and Usson (1992). ^28^

Software

• Voronoi Diagram Generator by Frederik Brasz
• ImageJ (RRID:SCR_003070) by NIH
• FIJI (RRID:SCR_002285) (Image J) by NIH
• Microsoft Windows 10 by Microsoft

**Step 1:** Generation of Voronoi diagrams (see *Note 1*)

**Figure 3.**
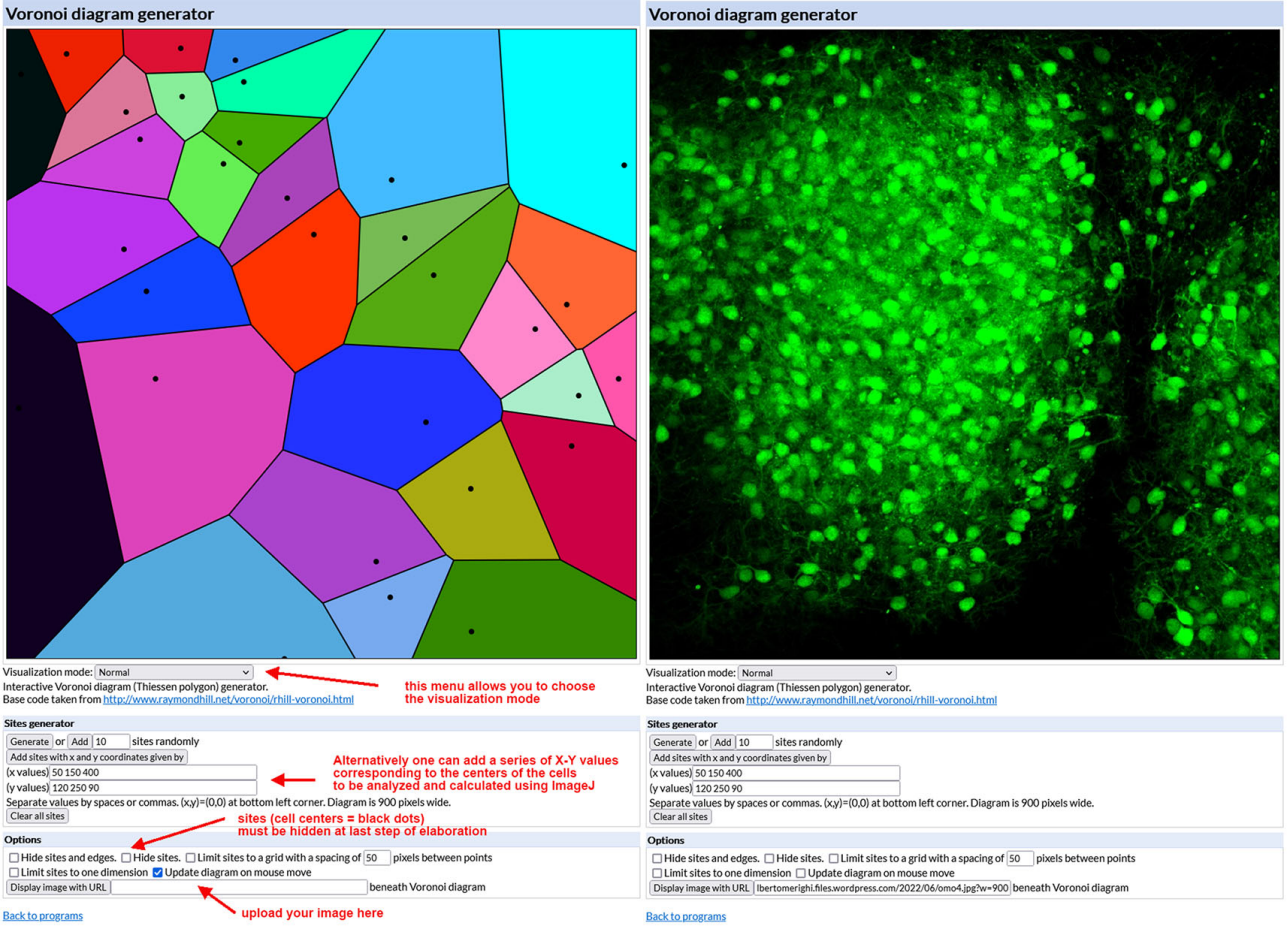
Use of the Voronoi diagram generator. *Left* – The mask of the Voronoi diagram generator with indications of its main commands and some hints for image elaboration. *Right* – The diagram generator with an uploaded example image of a single-cultured cerebellar slice from an L7-GFP *reln* ^(-/-)^ F1/mouse. GFP = green fluorescent protein.

**Figure 4.**
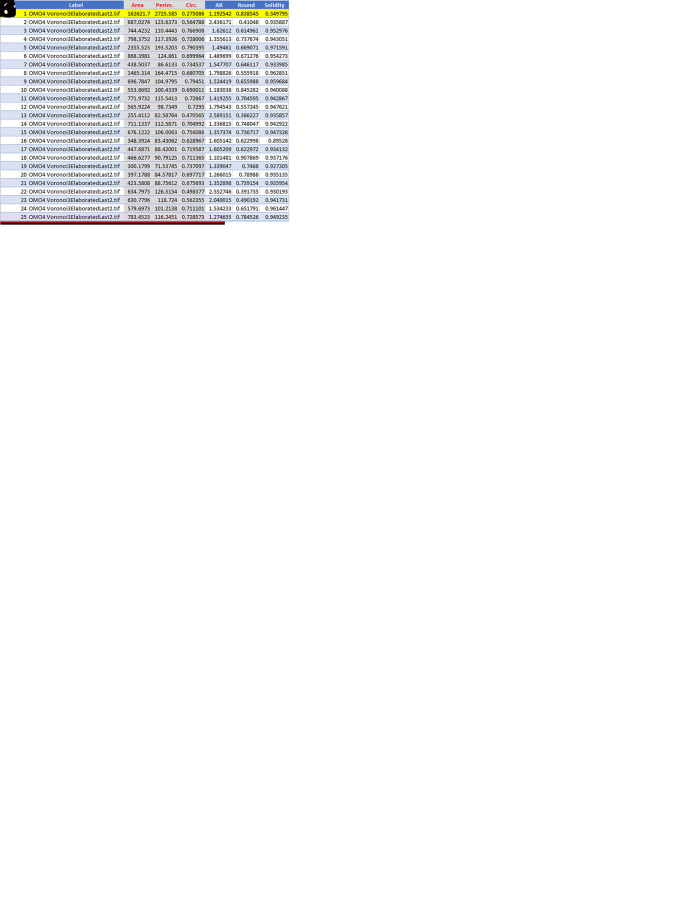
Elaboration of images for Voronoi analysis. The image is an example to show the individual steps of the technique. A: Generation of Voronoi polygons over the microscope image. Note that the center points (black dots) correspond to the cell centers; B: Color visualization of Voronoi polygons with center points; C: Color visualization of Voronoi polygons without center points; D: Elimination of the open polygons, *i.e.*, the polygons with one or more summits/sides outside the picture frame; E: Construction of the convex hull; F: Elimination of the polygons intersected by the convex hull. Note that the image in C (without center points) is used for this elaboration. This is because center points will be otherwise counted as particles by the ImageJ program in the subsequent elaboration; G: Elimination of the sides of the marginal polygons; H: Generation of the thresholded image to be elaborated by ImageJ with the Analyze Particles command; I: Generation of the overlay image with the indication of the number of each polygon analyzed by ImageJ. Note the number 1 circled in red at the center of the image. This number identifies the area in red in the following image; J: Image showing in red the area that ImageJ processes as a single particle. This area is discarded in the following elaborations; K: The values of Area, Perimeter, and Circularity (in red with gray background) of the first 25 particles (polygons) analyzed by ImageJ. In the example image processed here, ImageJ has analyzed a total of 318 particles of which particle #1 (highlighted in yellow) has to be discarded.

• Open the interactive Voronoi diagram (Thiessen polygon) generator. Figure 3 (left) shows the aspect of the generator mask.
• Upload the image to be analyzed (size must be 900×900 pixels and preferably saved as a PNG file). To do so your image has to be uploaded to the internet first ( *e.g.*, using Figshare or a personal website) so that it is possible to copy and paste its URL into the Voronoi generator. After uploading, the generator displays the image in its working space as shown in Figure 3 (right).
• Using the mouse, click above the center of each cell to generate the Voronoi polygons. Due to the thickness of the slice, cells that are below the plane of focus along the Z-axis cannot be easily distinguished. This introduces an error that can be neglected considering that only the perfectly focused cells are considered to define the sites for Voronoi analysis (see *Supplementary Material 2* ^36^). In the end, you will obtain the image shown in Figure 4A. Save the image on your computer (right-click on the image and choose “save” from the drop-down menu).
• Choose “Visualization Normal” from the Visualization mode drop-down menu of the generator. The tessellation appears as shown in Figure 4B. Again, save the image on your computer (right-click on the image and choose “save” from the drop-down menu).
• Select “Hide sites” from the Options menu of the generator. The tessellation appears as shown in Figure 4C as the black dots corresponding to cell centers have disappeared. Again, save the image on your computer (right-click on the image and choose “save” from the drop-down menu).

**Step 2:** Elimination of the marginal polygons

The principle at the basis of Voronoi tessellation is that a plane can be divided into regions close to each of a given set of objects, in our case the centers of the GFP-tagged PNs. Cell centers are mathematically referred to as sites (or seeds or generators). For each site, there is a corresponding region, called a Voronoi cell ^1^ (polygon), consisting of all points of the plane closer to that seed than to any other. In our case, PNs lay on a Euclidean plane (2D) and their centers form a discrete set of points. Due to the properties of the Voronoi partition, some polygons of the paving are not statistically representative of the set of polygons. Those polygons, referred to as the *marginal polygons* are associated with points located on the border of the cell population and have one or more summits that do not contain total information on their “surround”. Such summits are created by points that belong to a half-plane outside the image area. In other words, the marginal polygons must be excluded from analysis because one or two (in the corners of the microscope image) of their sides are indeed extending to the infinite (i.e. they are OPEN polygons) as they are defined by the perimeter of the image and NOT by the existence of another site outside the microscopic field (see *Supplementary Material 3* ^37^). Therefore, every point of the cell population whose associated polygon satisfies one of the two following conditions must not be considered in the subsequent computations:

• The polygon is open (the central point belongs to the convex hull). i.e. is a marginal polygon - see Figure 4D. Note that the software designs these polygons only because the image is a finite portion of the space, but the marginal polygons do not derive from the algorithm at the basis of the tessellation.
• At least one of the summits of the polygon is outside the convex hull - see Figure 4E.

The convex hull of a set of N points, *i.e.*, the centers of the cells, is defined as the smallest convex set that contains all of the points. In a 2D plane, it is a convex polygon whose vertices are points from N and which contains all points of N. It can be demonstrated that a Voronoi polygon is unbounded if and only if one of its points is on the convex hull (indicated by asterisks in Figure S3-1). As a corollary, the convex hull can be computed from the Voronoi diagram in linear time. Being unbounded, the polygons intersecting the convex hull do not have a finite area and thus cannot be used in the analysis. ^38^

• Elimination of the open polygons is carried out with Adobe Photoshop (RRID:SCR_014199) using the ***Magic Wand tool*** to select and erase them from the image shown in Figure 4B. The result is shown in Figure 4D.
• Construct the convex hull from the image in Figure 4D. The convex hull is constructed with the ***Line tool*** by drawing segments that join the site points (cell centers) of the eliminated open polygons so that there are no concavities, as shown in Figure 4E.
• Using Photoshop, eliminate the polygons intersected by the convex hull and the polygons with open sides using the image of Figure 4C (without cell sites). The result is shown in Figure 4F.
• Cancel the sides of the marginal polygons. Use the ***Magic Wand tool*** of Photoshop followed by the commands: ***Selection* → *Expand 2px***; ***Selection* → *Contract 1px***; ***Cancel***; ***Modify* → *Stroke*** ( ***color black***) ***2px***. You should obtain an image in which the area of the marginal polygons is empty as in Figure 4G. This is the last elaboration that will be used for the subsequent steps of analysis.

**Step 3:** Analysis of Voronoi polygons

• Open the image to be analyzed with ImageJ. Set the appropriate scale with ***Analyze* → *Set scale***.
• Run the following Macro by selecting ***Plugins* → *Macros* → *Run* → *Voronoi Macro*** ( Box 1).
• The macro enhances image contrast (optional – line 1), converts the image into a black and white (B&W) 8-bit image (line 2), finds the edges of the Voronoi polygons (line 3), and optimizes their contrast (lines 4-6) as shown in Figure 4H. It then sets up the measurements necessary for the following analysis of polygons: ***Area***, ***Shape descriptors***, and ***Perimeter*** (line 7). It also permits the creation of an image ( Figure 4I) with the overlay numerical indication of the individual polygons that the program has measured ( ***Add to overlay*** and ***Display label***). It also sets the number of Decimal places to 6 (line 7). Finally, the Macro performs the command Analyze Particles (line 8). Note the number 1 at the center of Figure 4I (encircled in red). This corresponds to the first counted particle that the program considers to be the ensemble of the marginal polygons (highlighted in red in Figure 4J). Note that the red circle is only added here for clarity but not displayed at the end of the elaboration by ImageJ.
• At the end of the Macro, save all computed values in a .csv or a .xls file (according to the version of ImageJ used). This file must then be converted into a .xlsx Microsoft Excel file.

Box 1. **Voronoi Macro.**

run("Enhance Contrast…", "saturated=2");

run("8-bit");

run("Find Edges");

//run("Brightness/Contrast…");

setMinAndMax(0, 0);

run("Apply LUT");

run("Set Measurements…", "area perimeter shape limit display redirect=None decimal=6");

run("Analyze Particles…", "display summarize add in_situ");

**Step 4:** Analysis of data

• Open the .csv or .xls file generated by ImageJ with Microsoft Excel (RRID:SCR_016137). A table extracted from the file is shown in Figure 4K. It contains the following information: Column A: progressive numbering of the particles (polygons) counted by ImageJ; Column B: Identification of the image analyzed; Column C: Area (in μm ^2^ if the Set scale command has been set properly); Column D: Perimeter (in μm if the Set scale command has been set properly); Column E: Circularity (or Roundness factor); Columns F-H: Other shape descriptors computed by ImageJ that are not used in the analysis. Note that line 2 (highlighted in yellow) corresponding to Particle 1 must be deleted (as indicated above).
• Save the file as a .xlsx file.
• Open the .xlsx file in Microsoft Excel and calculate the following:
  ○ **Mean of area**, **perimeter**, and **circularity** ( **roundness**)
  ○ **Standard deviation of area**, **perimeter**, and **circularity** ( **roundness**)
  ○ **Area Disorder** ( **AD**)
  ○ **Roundness Factor Homogeneity** ( **RFH**)
  The **mean circularity** ( **roundness**) ( **RF**_**av**_) is computed directly by the ImageJ program using the following formula:
$${\text{RF}}_{\mathrm{av}}=\frac{1}{N}\sum_{i=1}^{N}\frac{4\mathrm{\pi A}\left(X_{i}\right)}{L{\left(X_{i}\right)}^{2}}$$

where A(X) is the area and L(X) is the perimeter of the N polygons generated by the Voronoi generator. RFav is a pure number (0 < RF _av_ ≤ 1).

The AD is calculated as follows:

$$\mathrm{AD}=1-{\left(1+\frac{\sigma_{A}}{A_{\mathrm{av}}}\right)}^{-1}$$

where σ _A_ is the area standard deviation, and A _av_ is the mean area.

The RFH is calculated as follows:

$$\mathrm{RFH}={\left(1+\frac{\sigma_{\mathrm{RF}}}{{\mathrm{RF}}_{\mathrm{av}}}\right)}^{-1}$$

where σ _RF_ is the roundness factor standard deviation, and RF _av_ is the mean roundness factor.

Both are pure numbers with values >0 and ≤1.

• Transfer the values of RF _av_, AD, and RFH to a new Microsoft Excel spreadsheet for subsequent statistical analysis.

*Notes*

1. It is possible to use several other Voronoi generators that can be found online as freeware or in dedicated programs. We found it particularly advantageous to use this generator because one can directly upload the image to be analyzed and draw the sites, *i.e.*, the cell centers, straight on it. As an alternative, the X-Y coordinates of the sites can be uploaded in this and other generators. To do so one can use the ImageJ program and the ***Multipoint tool*** to obtain the spatial coordinates, i.e. the centers of mass of the cell nuclei, to be then uploaded to the Voronoi generator of choice (see *Supplementary Material 4* ^39^).

Protocol 2: Geographic Information Systems (GIS)-based spatial analysis

GIS-based technologies are used to store, view, analyze, and interpret geographic data. The location of features (i.e. the objects of interest in a study) is determined by geographic data, often also referred to as spatial or geospatial data. As microscopic images can be represented in an X-Y Cartesian coordinate system, GIS can be used for performing spatial analysis of specific biological features, i.e. the positions of the PNs in this study.

**Figure 5.**
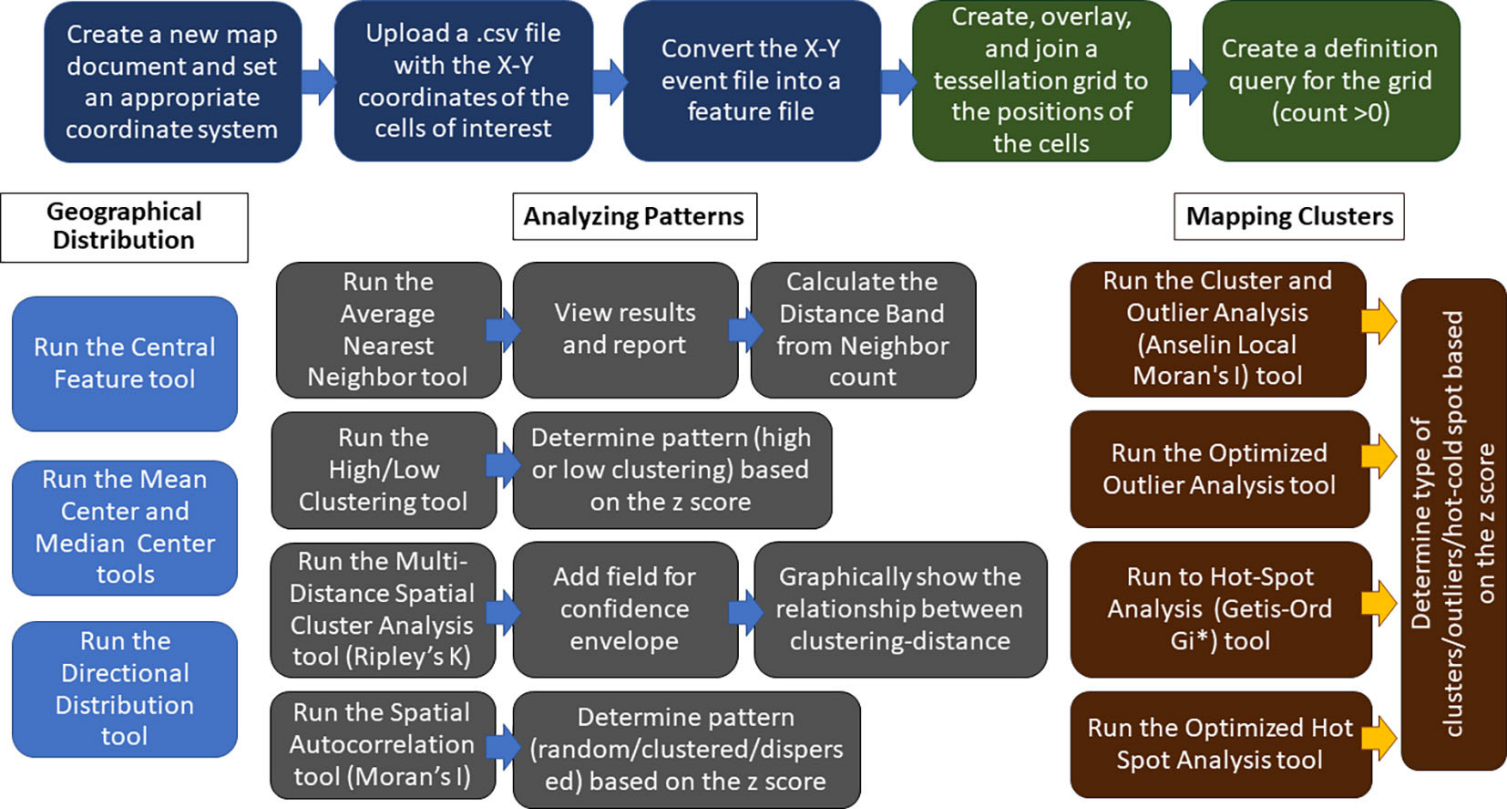
Flowchart of the steps of GIS-based analysis with ArcMap. The top row blocks show the preliminary steps to create a map starting from the X-Y coordinates of the PNs (blue blocks) and the steps of tessellation and joining of PN numbers to tessellated areas (green blocks). The other blocks show the main steps in the use of Geographic Distribution (light blue blocks), Analyzing Patterns (gray blocks), and Mapping Clusters (brown blocks) tools in ArcMap. Further details are given in the main text.

The protocols described here can be employed singularly or in combination to spatially analyze cellular clustering/dispersion, and use completely different approaches than Voronoi’s tessellation. A flowchart of the steps of GIS-based analysis is shown in Figure 5.

**Figure 6.**
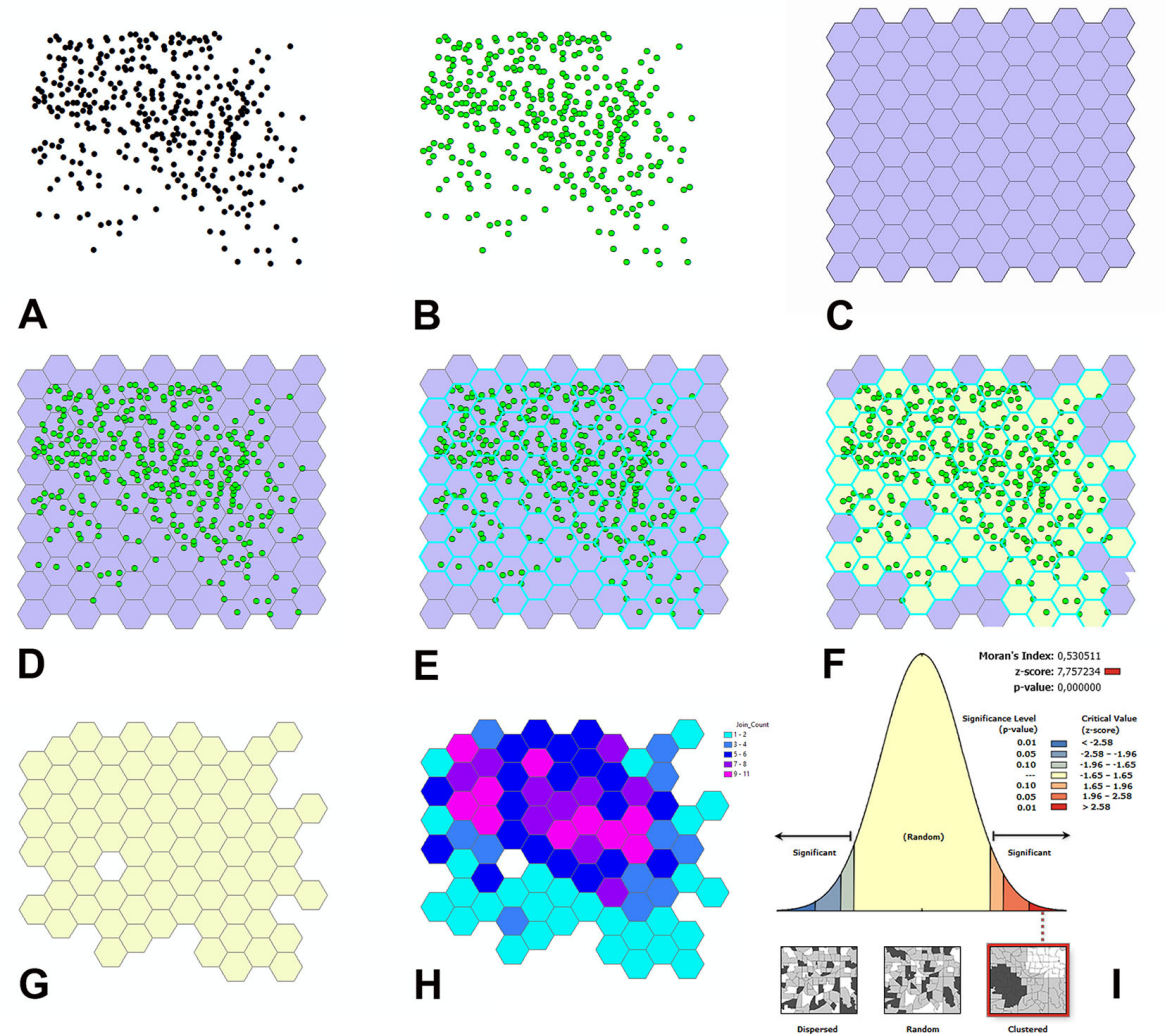
Main steps of the steps of GIS-based analysis with ArcMap. A: Position of the PNs as they appear in the X-Y event layer of ArcMap. B: The X-Y event layer is converted into a feature layer (using the symbology dialog sheet of the program, PNs are shown in green to stress the difference with the event layer in A). C: Tessellation of the study area. D: The tessellation is superimposed on the feature layer displaying the position of the PNs. E: Hexagons containing the PNs are highlighted with deep sky-blue margins. F: A new layer shows in a different color (gorse) the hexagons with the PNs. G: Selected visualization of the hexagons with the PNs for the subsequent graphical localization of cell counts on the map. H: Graphical visualization of cell counts on the map using a color ramp. Hexagons are displayed in different colors according to the number of PNs that they contain (see legend at top right). I: Example of the graphical output of Moran’s I tool.

**Figure 7.**
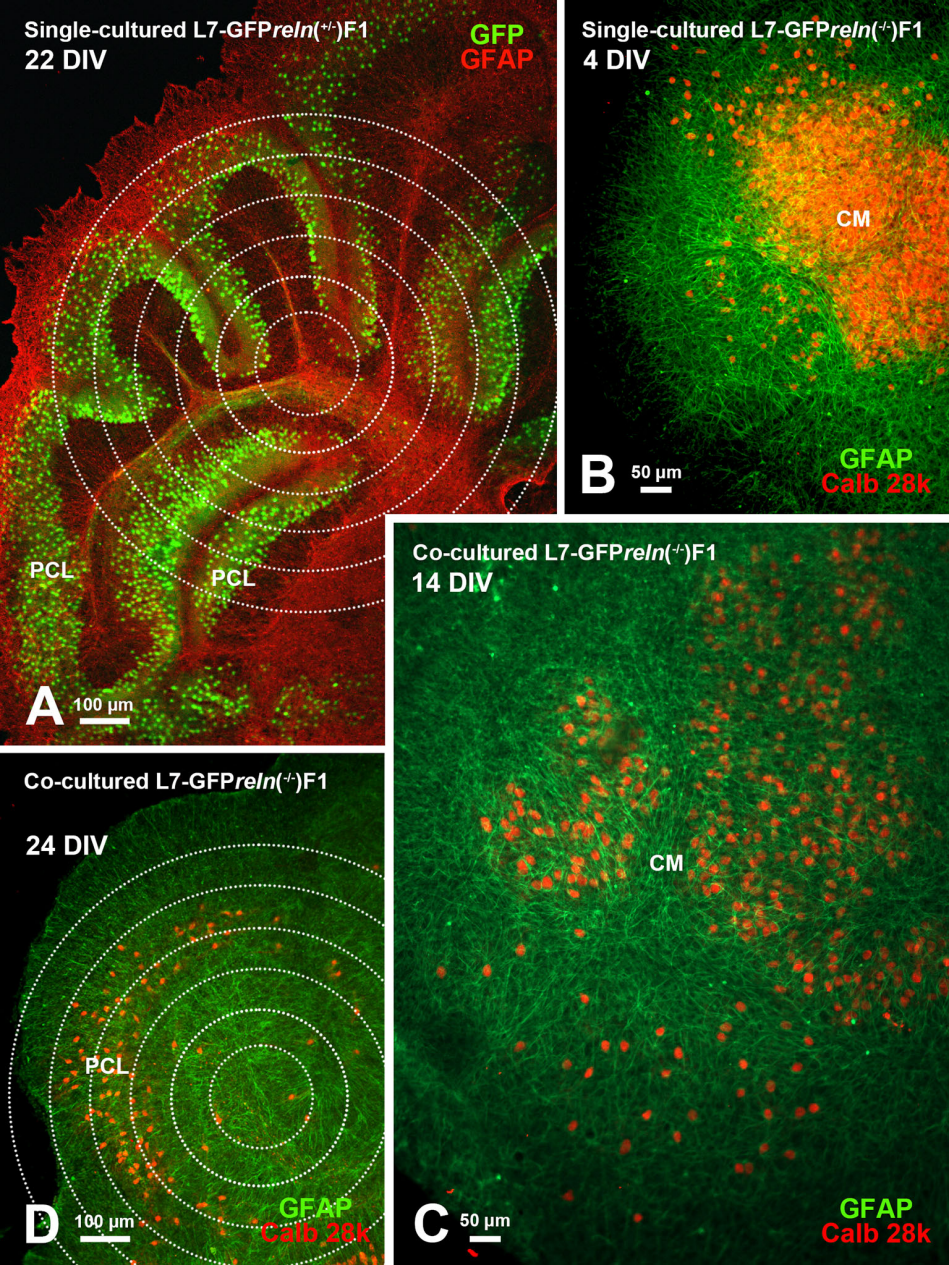
Histological aspects of single- and co-cultured slices after immunostaining with markers of PNs and glia. A: The cerebellar histology of a slice from an L7-GFP *reln* ^(+/-)^F1/ mouse shows a cortical stratification that is similar to that of an early postnatal wild-type mouse *in vivo.* The PNs are stratified to form a PCL composed of several layers of these neurons. B: A single-cultured slice from an L7-GFP *reln* ^(-/-)^F1/ mouse shows a large central mass of PNs. C-D: Exemplificative temporal evolution of a slice from an L7-GFP *reln* ^(-/-)^F1/mouse co-cultured with slices from L7-GFP *reln* ^(+/-)^F1/mice. The PNs are spread from the central mass (C) and try to form a multilayer PCL similar to that in A. Concentric circles in A and D are 100 μm spaced. Note that in B-D PNs have been stained for calb 28k and thus appear yellowish-orange for the superimposition of the green GFP signal and the red calb 28k fluorescence. *Abbreviations*: calb 28k = 28kD calbindin; CM = central mass; DIV = days *in vitro*; GFAP, Glial fibrillary acidic protein (red in A and green in B-D); GFP, green fluorescent protein; PCL = Purkinje cell layer; PNs = Purkinje neurons.

Figures 6 and 7 illustrate some key points along the use of the Analyzing Patterns and Analyze Clusters toolset of ArcMap. Additional information on the GIS tools used here can be found in *Supplementary Material 5*. ^40^

Software

• FIJI (RRID: SCR_002285) (Image J) by NIH
• ArcGIS for Desktop Basic (RRID: SCR_011081) by Esri

**Step 1:** Calculation of cells’ X-Y coordinates

• Open the image (see *Note 1*) to be analyzed using FIJI: ***File* → *Open***
• Set the appropriate scale for the image using ***Analyze*** → ***Set Scale***. In the pop-up window report the distance in pixels related to the known distance using the correct unit of length (μm). Leave the pixel aspect ratio at 1.0 (see *Note 2*).
• Use the ***Multipoint tool*** and click with the mouse on the center of each labeled PN. The tool should be configured so that clicked cells are visualized directly on the image. To do so double-click with the mouse on the tool icon and tick the *Label points* box.
• Set the measurements to be computed using ***Analyze* → *Set Measurements***. In the pop-up window verify that all boxes are not ticked. Set the *Decimal places* box to 3. Use ***Analyze* → *Measure*** to calculate the X-Y coordinates. A new window pops up where the results are shown in tabular form.
• Save data as a.csv file.

**Step 2:** Image elaboration

• Load all .csv files to an *ad hoc* folder in ArcMap.
• Open the program and create a new map document: ***File* → *New* → *New Maps* → *My Templates* → *Blank Map***.
• On the ribbon click ***View* → *Data Frame Properties***. In the pop-up window click ***Coordinate System***. Click on the *world* icon and select ***New* → *Projected Coordinate System.*** For *Name* type a name for the new coordinate system e.g. *Microscope Coordinate System.* For *Linear Unit Name* choose *Millimeter. Leave all other parameters unchanged* (see *Note 2*) *.* Save the new coordinate system. The program creates a new folder named *Custom* with the file *Microscope_Coordinate_System.*
• Save the file and name it with the name used for the image under investigation.
• Add the X-Y coordinates of the cells to the map. On the toolbar click the **Add data icon**, then → **Add Data**. Choose the .csv file with the X-Y coordinates of the cells and upload it. The program creates a new layer on the map with the same name as the .csv file and an attribute table containing the cell coordinates. Right-click with the mouse on the new layer and choose ***Display XY Data***. In the pop-up window, be sure that the X and Y fields for the layer correspond to the fields of X and Y coordinates in your .csv file, and press the ***OK*** button. A window appears with the warning *Table Does Not Have Object-ID Field.* This is because the layer created so far is an *XY event layer* that must be converted into a *feature layer* for further analysis. Press the ***OK*** button and the positions of the cells will be displayed (see Figure 6A).
• Convert the XY event layer into a feature layer. On the layer right-click → ***Data*** → ***Export Data*** → ***All features***. Select ***Use the same coordinate system as this layer source data*** and press ***OK***. The program generates a new layer named **Export_Output_#** (see *Note 3*). By double-clicking with the mouse on the layer name, the *Layer Properties* window opens and it is possible to customize the data by e.g. changing the layer name and using a different symbology to display the cells (see Figure 6B).

**Step 3:** Visualization of cell counts and preliminary steps for subsequent analyses

• Generate tessellation. With the mouse select the ***Export_Output #*** layer. On the toolbar select the ***ArcToolbox*** icon then → ***Data Management Tools*** → ***Sampling*** → ***Generate Tessellation***. This tool generates a polygon feature class of a tessellated grid of regular polygons which will entirely cover a given extent. In the pop-up window *leave unchanged the path of the Output Feature Class.* For *Extent* click on the folder icon and choose ***Same as layer Export Output #***. Selection of the shape type is optional. Check that **HEXAGON** is selected by the program. The program creates a new layer named ***Generate Tessellation #*** and the tessellation appears above the cells (see Figure 6C-D).
• Select hexagons with cells. From the ribbon click ***Selection*** → ***Select by location***. In the pop-up window, for the *Target layer(s)* select ***Generate Tessellation #***, for the *Source layer* select ***Export_Output #***, and for the *Spatial selection method for target layer feature(s)* choose ***Intersect the source layer feature***. Click OK. The hexagons containing cells are highlighted (see Figure 6E).
• Join cell positions to selected hexagons (see *Note 4*). On the toolbar select the ***ArcToolbox*** icon then → ***Analysis Tools*** → ***Overlay*** → ***Spatial Join***. In the pop-up window, for *Target features* select ***Generate Tessellation #***, for *Join Features* select ***Export_Output #***. The program creates a new layer named ***Export_Output #* SpatialJoin#** with the hexagons containing cells visualized in a different color than those with no cells (see Figure 6F).
• Graphical visualization of the cell counts on the map. Remove the layer ***Generate Tessellation #*** from the map (right-click with the mouse) and turn off the visibility of the layer ***Export Output #***. Only the hexagons with cells remain visible (see Figure 6G). With the mouse right-click on layer → ***Properties*** → ***Symbology*** → ***Show quantities*** → ***Select color ramp*** (e.g. cyan-to-purple) → ***Fields***: ***Value = Join-Count***; ***Normalization = none***. Hexagons on the map are displayed in different colors according to the number of cells that they contain (see Figure 6H).

**Step 4:** Analysis of the geographical distribution of the PNs

We have used five tools to measure a set of features that allowed us to analyze some characteristics of the distribution of the PNs and compare them among the three experimental groups of this study. The procedures for the use of these tools follow a series of similar steps that, for brevity, are reported in Table 2 below (see *Supplementary Material 5* ^40^).

**Table 2.** Steps and settings in the use of the Measuring Geographic Distributions tools of ArcMap.

Tools	Input Feature Class	Output Feature Class	Distance Method	Ellipse/Circle Size
Central Feature	Export_Output_# ( *see* the last step of Step 2 above)	Export_Output_# CentralFeat	EUCLIDEAN DISTANCE	Not applicable
Mean Center		Export_Output_# MeanCenter	Not applicable	
Median Center		Export_Output_# MedianCenter		
Directional Distribution		Export_Output_# Directional		1 STANDARD DEVIATION
Standard Distance		Export_Output_# StandardDis		

**Step 5:** Analysis of the pattern of cell distribution

• Average Nearest Neighbor (see *Notes 5-6*)

The *Average Nearest Neighbor tool* calculates the nearest neighbor index based on the average distance from each PN to its nearest neighboring PN.

• On the toolbar select the ***ArcToolbox*** icon then → ***Spatial Statistics Tools*** → ***Analyzing Patterns*** → ***Average Nearest Neighbor***
• In the pop-up windows for *Input Feature Class* select ***Export Output #***, for the *Distance Method*, select ***EUCLIDEAN DISTANCE***, and tick the box *Generate Report* so that the tool creates an HTML report file with a graphical summary of results.
• High/Low Clustering (G tool - see *Notes 5*-6)

The High/Low Clustering tool measures the degree of spatial clustering of the PNs for either high or low values using the Getis-Ord General G statistic. ^41^

• On the toolbar select the ***ArcToolbox*** icon then → ***Spatial Statistics Tools*** → ***Analyzing Patterns*** → ***High/Low Clustering (Getis-Ord General G)***
• In the pop-up windows for *Input Feature Class* select ***Export Output #***, for *Input Field* select ***XM***, for *Conceptualization of Spatial Relationship* select ***INVERSE DISTANCE***, for *Distance Method*, select ***EUCLIDEAN DISTANCE***, for *Standardization* select ***NONE***, and tick the box *Generate Report* so that the tool creates an HTML report file with a graphical summary of results.
• Spatial Autocorrelation (Global Moran’s I - see *Notes 5*-6)

The Spatial Autocorrelation (Global Moran’s I) tool measures spatial autocorrelation based on PN locations and attribute values using the Global Moran’s I statistic. ^42^

• On the toolbar select the ***ArcToolbox*** icon then → ***Spatial Statistics Tools*** → ***Analyzing Patterns*** → ***Spatial Autocorrelation (Global Moran’s I)***.
• In the pop-up windows for *Input Feature Class* select ***GenerateTessellation#Spati#***, for *Input Field* select ***Join-Count***; Tick ***Generate Report***, for *Conceptualization of Spatial Relationship* select ***INVERSE_DISTANCE***, for *Distance Method* select ***EUCLIDEAN DISTANCE***, for *STANDARDIZATION* select ***ROW***. Click ***OK***.
• After the tool has run, no layer is added to the map but a report is generated. To view the report in the ribbon, click ***Geoprocessing*** → ***Results***.
• In the results list expand the Spatial Autocorrelation (Moran’s I) folder and click on ***Report File*** ( Figure 6I) to view the report in a browser window. The report file is automatically saved as a.html file in the ArcGIS folder of the computer (see *Note* 7).
• Multi-distance Spatial Cluster Analysis (Ripley’s K Function)

The Multi-Distance Spatial Cluster Analysis (Ripley’s K Function) determines whether PNs exhibit statistically significant clustering or dispersion over a range of distances.

• On the toolbar select the ***ArcToolbox*** icon then → ***Spatial Statistics Tools*** → ***Analyzing Patterns*** → ***Multi-distance spatial cluster analysis (Ripley’s K function)***
• In the pop-up windows for *Input Feature Class* select ***Export Output #***, for *Output Table* leave the program generated name ( ***Export Output # MultiDistan)***, for *Compute Confidence Envelope* (optional) choose ***99_PERMUTATIONS***, tick the box ***Display Results Graphically***
  The graph should be exported in JPEG format at a size of *900×510 pixels* if used for publication. In the *Options* window select Quality 100% and 300 DPI.

*Notes*

1. All images to be analyzed should be of the same pixel size and at the same magnification if one wants to further compare the results of the analysis of individual images within a single experimental group and/or between groups. As we aimed to compare the results of the GIS approach with those of Voronoi analysis we have used images of 900×900 pixels at a resolution of 200 dpi.
2. It is important to set the appropriate scales of the images because it may be possible that not all images are acquired at the same magnification or with the same microscope. Set the unit of length in microns (μM) in the FIJI dialog window.
  The ArcMap Coordinate System dialog window allows you to add a new customized coordinate system but does not permit you to set the Linear Unit in microns. It is advisable to set the Linear Unit of the Microscope Coordinate System in Millimeters. With these settings, the output of ArcMap elaborations is nominally in millimeters, but actually in microns.
3. There may be differences in the way the coordinate system is displayed according to the version in use of the software. Be sure that the following parameters are applied:
  Projection: Transverse_Mercator
  False_Easting: 0.0
  False_Northing: 0.0
  Central_Meridian: 0.0
  Scale_Factor: 1.0
  Latitude_Of_Origin: 0.0
  Linear Unit: Millimeter (0.001)
4. Most tools used for spatial analysis require entering an *Input Field.* In our analysis, after applying the Spatial Join tool, this field reports the number of GFP-tagged PNs per tessellation hexagon. For the analysis of spatial clustering, it is of interest to analyze the density of the GFP-tagged PNs (# positive PNs/area) and not their absolute numbers.
5. If the image scale and the coordinate system are set as indicated in *Note 2* the tool output will be indicated in millimeters but it will correspond to microns (μm).
6. For every session of use the program numbers progressively the operations done and the map layers. Layers can be renamed by opening the *Layer Properties* window (mouse right-click) and then selecting *General.*
7. The program saves the Moran’s I report files in the following format MoransI_Result_####_####.html. It is advisable to rename these files to properly refer them to the image of origin.

**Step 6:** Mapping PN clusters

• Cluster and Outlier Analysis (Anselin Local Moran’s I).

The Cluster and Outlier Analysis tool identifies spatial clusters of PNs, with high or low values. The tool also identifies spatial outliers (See also *Supplementary Material 5* ^40^).

• On the toolbar select the ***ArcToolbox*** icon then → ***Spatial Statistics Tools*** → ***Mapping Clusters*** → ***Cluster and Outlier Analysis (Anselin Local Moran’s I)***.
• In the pop-up windows for the *Input Feature Class* select ***GenerateTessellation#Spati#***, for the *Input Field* select ***Join-Count***; for *Conceptualization of Spatial Relationship* select ***INVERSE_DISTANCE***, for *Distance Method* select ***EUCLIDEAN DISTANCE***, for *STANDARDIZATION* select ***ROW***. Click ***OK***.
• After the tool has run, a new layer is added to the map displaying in different colors statistically significant clusters and outliers for a 95 percent confidence level based on the local I index (Figure S5) – See *Note 1.*
• Optimized Outlier Analysis

This tool identifies statistically significant spatial clusters of high values (hot spots) and low values (cold spots) as well as high and low outliers. It automatically aggregates incident data, identifies an appropriate scale of analysis, and corrects for both multiple testing and spatial dependence (See also *Supplementary Material 5* ^40^).

• On the toolbar select the ***ArcToolbox*** icon then → ***Spatial Statistics Tools*** → ***Mapping Clusters*** → ***Optimized Outlier Analysis.***
• In the pop-up windows for *Input Feature Class* select ***GenerateTessellation#Spati#***, for *Input Field* select ***Join-Count***; for *Conceptualization of Spatial Relationship* select ***INVERSE_DISTANCE***, for *Distance Method* select ***EUCLIDEAN DISTANCE***, for *STANDARDIZATION* select ***ROW***. Click ***OK***.
• After the tool has run, a new layer is added to the map displaying in different colors statistically significant clusters and outliers based on their z-scores (Figure S5) – See *Note 1.*
• Hot Spot Analysis (Getis-Ord Gi*)

The Hot Spot Analysis tool calculates the Getis-Ord Gi* statistic of the PN spatial distribution. The resultant z-scores and p-values show where high or low numbers of PNs cluster spatially (See also *Supplementary Material 5* ^40^).

• On the toolbar select the ***ArcToolbox*** icon then → ***Spatial Statistics Tools*** → ***Mapping Clusters*** → Hot Spot Analysis (Getis-Ord Gi*).
• In the pop-up windows for *Input Feature Class* select ***GenerateTessellation#Spati#***, for *Input Field* select ***Join-Count***; for *Conceptualization of Spatial Relationship* select ***INVERSE_DISTANCE***, for *Distance Method* select ***EUCLIDEAN DISTANCE***, for STANDARDIZATION select ***ROW***. Click ***OK***.
• After the tool has run, a new layer is added to the map (Figure S5) displaying in different colors statistically significant spatial clusters of high values (hot spots) and low values (cold spots) based on their z-scores – See *Note 1.*
• Optimized Hot Spot Analysis

The Hot Spot Analysis tool calculates the Getis-Ord Gi* statistic of the PN spatial distribution. It evaluates automatically the characteristics of the input feature class to produce optimal results (See also *Supplementary Material 5* ^40^).

• On the toolbar select the ***ArcToolbox*** icon then → ***Spatial Statistics Tools*** → ***Mapping Clusters*** → ***Optimized Hot Spot Analysis***.
• In the pop-up windows for *Input Feature Class* select ***GenerateTessellation#Spati#***, for *Input Field* select ***Join-Count***; for *Conceptualization of Spatial Relationship* select ***INVERSE_DISTANCE***; for *Distance Method* select ***EUCLIDEAN DISTANCE***; for *STANDARDIZATION* select ***ROW***. Click ***OK***.
• After the tool has run, a new layer is added to the map (Figure S5) displaying in different colors statistically significant spatial clusters of high values (hot spots) and low values (cold spots) based on their z-scores – See *Note 1.*

*Notes*

• 1. It is possible to change the type of graphical visualization by right-clicking with the mouse on the new layer and opening the *Symbology* window to display the spatial localization and values of the z-score and the p-values as shown in Figure S5.

## Results

### Histology

Organotypic cultures from L7-GFP *reln *F1/ ( Figures 1 and 2) ^43^^–^^45^ permit a dynamic study of the effects of Reelin on neuronal migration and lamination of the cerebellar cortex. Thanks to GFP fluorescence, PNs can be visualized without the need for immunocytochemical labeling. In addition, our approach makes it unnecessary to use several groups of mice to be sacrificed at given postnatal ages to properly follow the cerebellar maturation. Figure 1 shows two co-cultured slices from a *reln* ^(+/-)^ F1/ mouse (top right) and a *reln* ^(-/-)^ F1/ mouse (bottom left). A comparison of the histology of the two slices permits clear identification of the phenotypic differences deriving from the genetic backgrounds of the donor mice. The figure also shows that at the end of the culture period slices can be easily subjected to immunocytochemical staining. Figure 2 shows the modifications over time in a single-cultured slice from an L7-GFP *reln* ^(-/-)^ F1/mouse. After 29 days *in vitro,* the mass of the GFP fluorescent PNs tends to spread from the center of the slice but the neurons do not migrate to form a layered structure. Figure 7 shows that in co-cultures the architecture of the slice derived from a homozygous *reln* ^(-/-)^ mouse ( Figure 7B- D) progressively changes to eventually become related to that from a heterozygous *reln* ^(+/-)^ mouse ( Figure 7A).

### Voronoi’s analysis of cellular sociology

**Figure 8.**
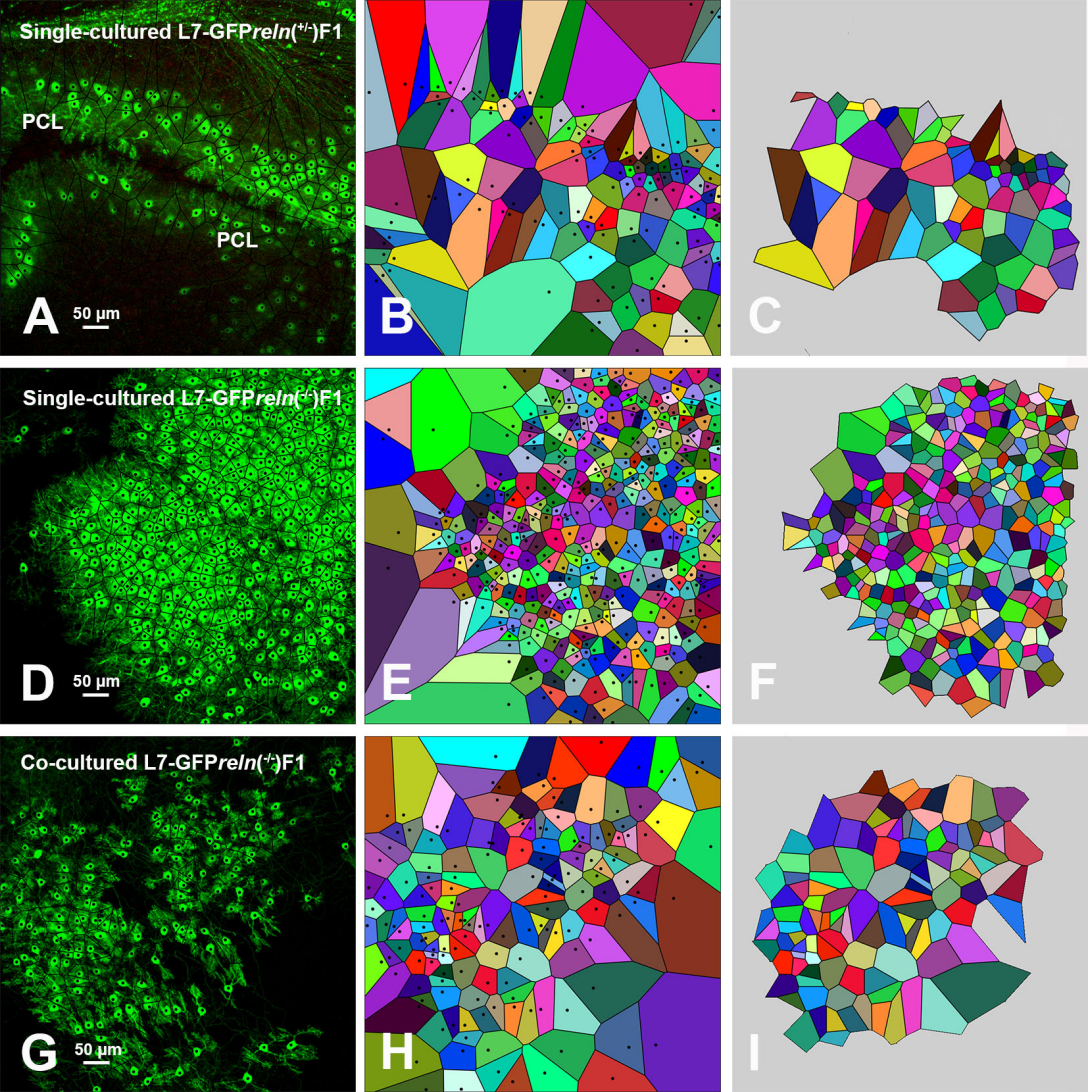
Voronoi tessellation of slices. Confocal images of the GFP-tagged PNs with superimposed Voronoi polygons (A, D, and G) in 21 DIV slices. Polygons were generated and further elaborated as described in the Methods section and protocols.io. Images in B, E, and H show the initial elaboration of Voronoi polygons; those in C, F, and I show the last step of elaboration with the exclusion of the marginal polygons that are outside the convex hull. It can be seen that polygons are smaller and have a more homogeneous size in single cultured slices from L7-GFP *reln* ^(-/-)^F1/ mice (D-F), become larger and have less homogeneous sizes in co-cultured slices from L7-GFP *reln* ^(-/-)^F1/ mice (G-I), whereas slices from L7-GFP *reln* ^(+/-)^F1/ mice display larger polygons of quite homogeneous sizes. Black dots in A-B, D-E, and G-H are the centers of mass of the PNs. They have been cleared in the subsequent elaboration (C, F, and I) to avoid interference with automated counting. Colorization is solely used for better visualization of polygons. *Abbreviations*: GFP = green fluorescent protein; PCL = Purkinje cell layer; PNs = Purkinje neurons.

**Figure 9.**
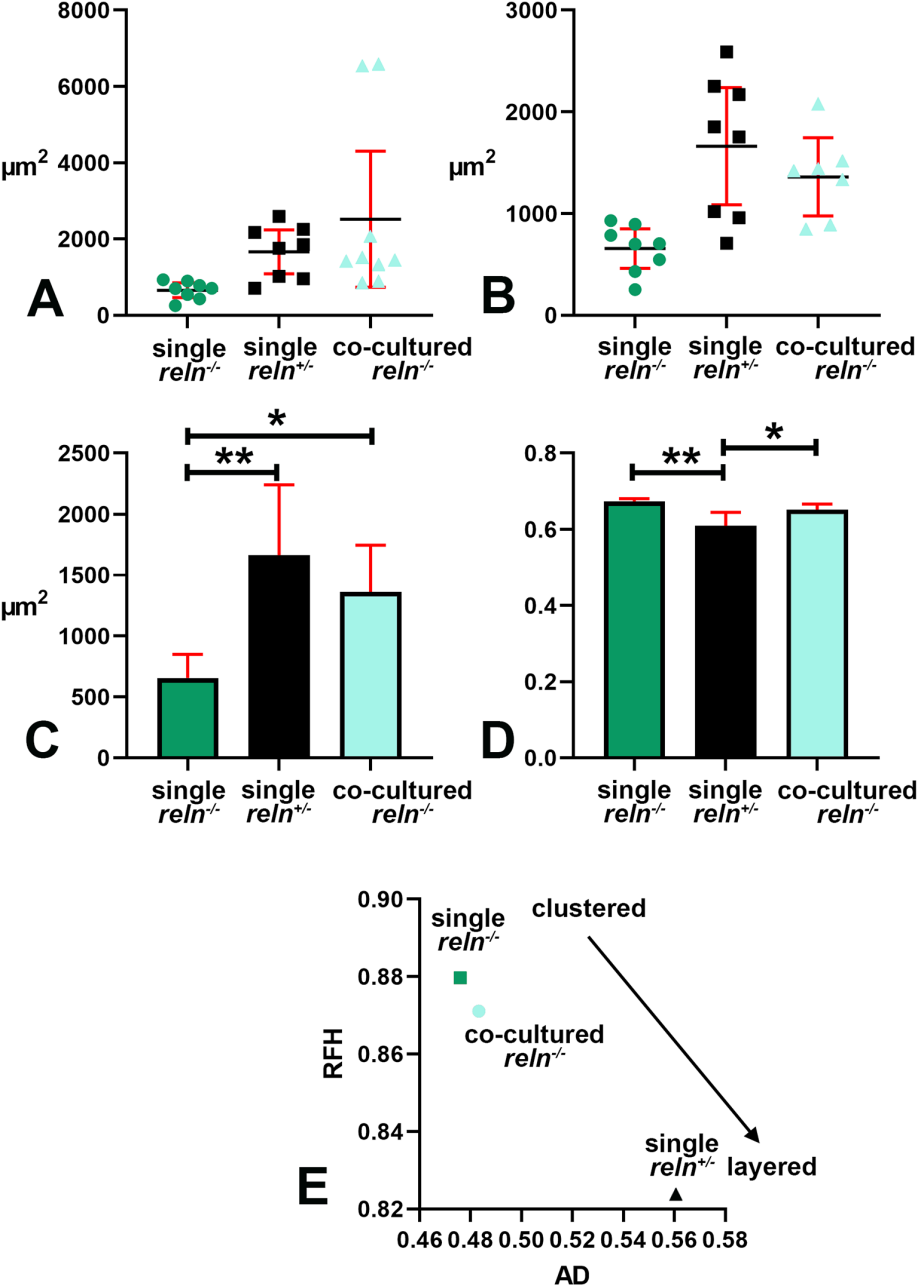
Quantitative analysis of Voronoi tessellation. A-B: Descriptive statistics of the mean areas of Voronoi polygons. Note that there is little variability in the mean areas of polygons among singularly cultured slices obtained from L7-GFP *reln* ^(-/-)^ F1/ mice as PNs remain aggregated into a central mass deep to the cerebellar cortex (see Figures 1, 2, 7, 8, and 10); in the two other groups of cultures values are more dispersed and this indicates the spread of the PNs to form the PCL that will be typical of the mature cortex. A: Raw data plotted without any adjustment; B: Cleaned data after removing the two outliers identified in the co-cultured slices from L7-GFP *reln* ^(-/-)^ F1/ mice with the ROUT method (Q = 1%). Error bars are 95% confidence intervals. Data passed the Kolmogorov-Smirnov normality test. C: Ordinary one-way ANOVA [F(2, 20) = 8.966; P value = 0.0017] followed by Tukey’s multiple comparison test shows that in co-cultured slices from L7-GFP *reln* ^(-/-)^ F1/ mice the mean polygon area is larger than in single-cultured slices from animals with the same genetic background (mean ± 95% CI: 1,361 ± 385 μm ^2^ *versus* 656 ± 195 μm ^2^, adjusted P value = 0.0287) and becomes closer to that of polygons in slices of L7-GFP *reln* ^(+/-)^ F1/ mice (mean ± 95% CI: 1,361 ± 385 μm ^2^ *versus* 1,663 ± 576 μm ^2^, adjusted P value = 0.4678). This observation confirms quantitatively the dispersion of the PNs in co-cultured slices from L7-GFP *reln* ^(-/-)^ F1/ mice that lack Reelin, but are exposed to the protein produced *ex vivo* by the slices from L7-GFP *reln* ^(+/-)^ F1/ mice. Also note the difference in mean areas of Voronoi polygons in single cultures of slices explanted from *reln* ^(-/-)^ mice *versus* mice *reln* ^(+/-)^ (mean ± 95% CI: 56 ± 195 μm ^2^ *versus* 1,663 ± 576 μm ^2^, adjusted P value = 0.0014). n (number of slices from five different mice) = 8; * 0.05≤ adjusted P value >0.01; ** 0.001≤ adjusted P value >0.001. D: Brown-Forsythe [F* (2, 10.38) =11.41, P value = 0.0024] and Welch [W (2, 11.78) =11.94, P value = 0.0015) ANOVA tests of RFav. Since Voronoi polygons are convex, the average type of spatial occupation of the PNs is well-characterized by the RFav (mean circularity). In slices from single-cultured L7-GFP *reln* ^(-/-)^ F1/ mice, the RFav of the Voronoi polygons is higher than that of the polygons in slices from co-cultured slices of the same genetic background (mean ± 95% CI: 0.6733 ± 0.007 *versus* 0.6515 ± 0.0149, adjusted P value = 0.0314) and single-cultured L7-GFP *reln* ^(+/-)^ F1/ mice (mean ± 95% CI: 0.6733 ± 0.007 *versus* 0.6091 ± 0.0298, adjusted P value = 0.0082). On the other hand, the difference in RFav between single-cultured slices from L7-GFP *reln* ^(+/-)^ F1/ mice and co-cultured slices from L7-GFP *reln* ^(-/-)^ F1/ mice is not statistically significant (mean ± 95% CI: 0.6091 ± 0.0298 *versus* 0.6515 ± 0.0149, adjusted P value = 0.0718). The RF of a circle is 1 while that of a line is 0. Therefore, our analysis confirms mathematically that in single-cultured slices from L7-GFP *reln* ^(+/-)^ F1/ mice and in co-cultured slices from L7-GFP *reln* ^(-/-)^ F1/mice there is a tendency to the alignment of the PNs, whereas in single-cultured slices from mice that lack Reelin the population of the PNs displays a spatial occupation consistent with the formation of a mass of cells in the cerebellar white matter. E: X-Y diagram showing topographical information of the PN population in the three experimental groups of cerebellar slices under the different culturing conditions reported in the Materials and Methods section. The X-axis displays the values of AD. AD varies when the value of the intrinsic disorder ( *i.e.*, the heterogeneity of the Voronoi polygon areas) increases and a given value of AD corresponds to a given value of intrinsic disorder for any cell population. The Y-axis displays the values of RFH that vary in parallel to geometric disorder, *i.e.*, the homogeneity/inhomogeneity of the circularity of the Voronoi polygons. Both AD and RFH vary from 0 to 1. A highly ordered population is characterized by values of AD and RFH, respectively, corresponding to 0 and 1, ^27^ this means that all the polygons have the same area and circularity. The AD and RFH values are typical of a highly ordered population (high RFH, low AD) when one analyzes the clustered population of the PNs forming the central mass in the single-cultured *reln* ^(-/-)^ slices. When the PNs align to eventually form a well-defined layer in the *reln* ^(+/-)^ slices from heterozygous mice, the RFH diminishes and becomes closer to that of a line (=0), whereas the AD increases because the Voronoi polygons are small where the PNs tend to be aligned, but larger in the other parts of the slice (see Figure 8A- C). Note that in the co-cultured slices from *reln* ^(-/-)^ mice, there is a shift towards the values observed for the slices from *reln* ^(+/-)^ heterozygous mice. Sample sizes (# slices): single-cultured *reln* ^(-/-)^ and *reln* ^(+/-)^ slices, 8; co-cultured *reln* ^(-/-)^ slices, 9. *Abbreviations*: PNs = Purkinje neurons; PCL = Purkinje cell layer; RF = roundness factor; RFav = mean roundness factor; AD = area disorder; RFH = roundness factor homogeneity.

Voronoi’s partition allowed for quantifying the dispersion of PNs in the presence or absence of Reelin. Starting from a set of points locating the position (center of mass) of the cell nuclei it was possible to obtain information on the order/disorder of the PN population ( Figure 8). Polygon areas in single-cultured slices from *reln* ^(+/-)^ animals ( Figure 8A- C and Figure 9A- C) are larger than those from *reln* ^(-/-)^ mice ( Figure 8D- F and Figure 9A- C). Conversely, in co-cultures polygon areas in *reln* ^(-/-)^ slices ( Figure 8G- I and Figure 9A- C) become larger than in single-cultured *reln* ^(-/-)^ slices, confirming a better dispersion of PNs in the presence of Reelin. RFav is not different in *reln* ^(-/-)^ slices under different culture conditions indicating that the geometry of the polygons was unchanged ( Figure 9D). We have also plotted AD and RFH in X/Y diagrams to show the spatial behavior of the PNs in the three groups of cultures ( Figure 9E) and established that, in the co-cultures, the Reelin provided by the *reln* ^(+/-)^ slices was sufficient to produce a measurable shift in the distribution of the PNs in *reln* ^(-/-)^ slices from the pattern observed when the latter are cultivated singularly.

### GIS spatial statistic

As indicated in the flowchart of Figure 5, we have used a series of GIS tools to analyze the differences among the three groups of cultures regarding the geographic distribution of the PNs, the general patterns of clustering of these neurons, and the type/topography of the PN clusters.

### Geographic distribution (see also *Supplementary Material 5* ^40^)

**Figure 10.**
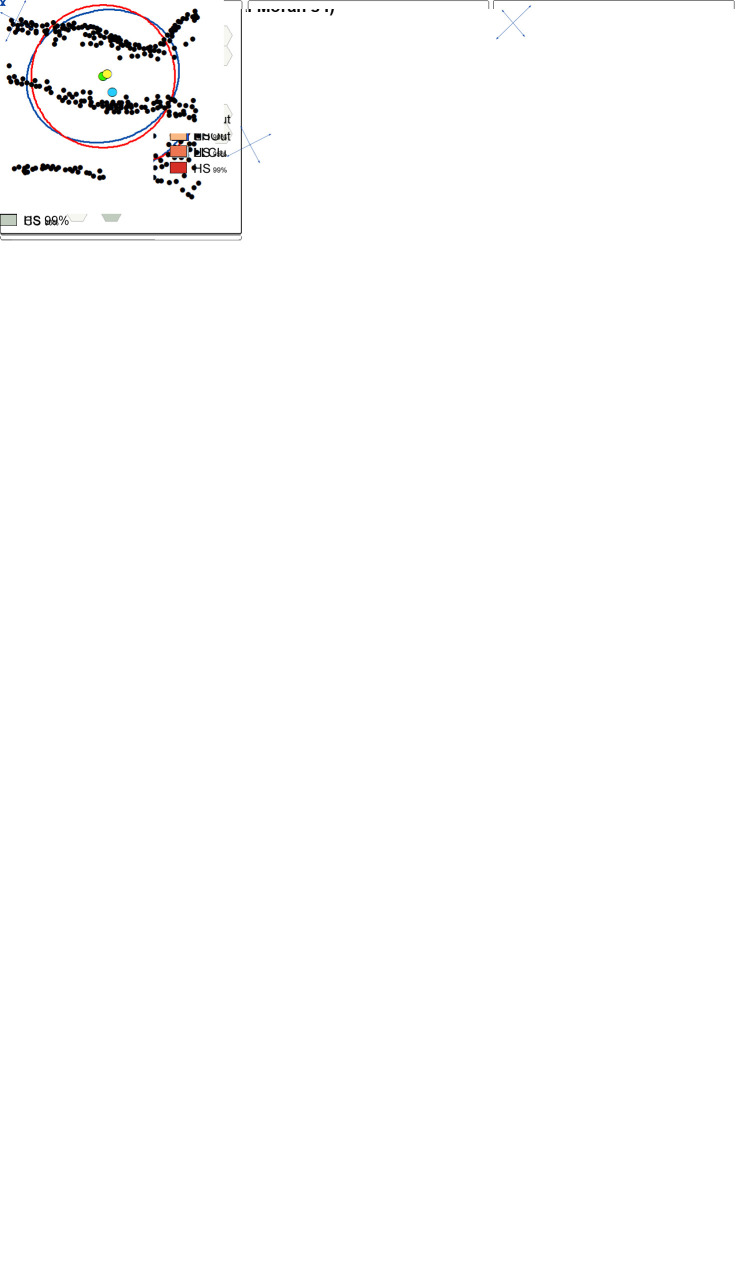
Exemplificative images of GIS spatial statistic analysis. A-C: Original images of slices from mice of different experimental groups. Simple observation shows the differences in the dispersion of the PNs according to the phenotype and culture conditions. D-F: Geographic distribution analysis. Note the difference in the position of the central feature (deep sky-blue dot), the median center (gorse dot), and the mean center (harlequin green dot). The X- and Y-axes of the deviational ellipses (cobalt blue) are 400,67 μm and 490.28 μm (D), 554.39 μm and 474.95 μm (E), 512.94 μm and 370.12 μm (F). The radius of the standard distance circle (red) is 223.87 μm (D), 258.10 μm (E), and 223.63 (F). G-I: Identification of tessellation hexagons using a color ramp scale based on the joint count of the PNs inside each hexagon. J-L: Localization of the different types of clusters and outliers (Anselin local Moran’s index) in slices from the three experimental groups of the study. M-O: Localization of cold and hot spots (Getis-Ord G*) in slices from the three experimental groups of the study. Percentages indicate the confidence values. *Abbreviations*: CF = central feature; CS = cold spot; DD = deviational distance; HHClu = high-high cluster; HLOut = high-low outlier; HS = hot spot; LHOut = low-high outlier; LLClu = low-low cluster; MC = mean center; MeC = median center; NS = not significant; SD = standard distance.

Once we had geolocalized the PNs in the Euclidean space of the microscope field, we studied the geographic distribution of the PNs with the Measuring Geographic Distributions toolset ( Figures 10D-F and S5-1). Panels D-F in Figure 10 display, as an example, the results of the geographic distribution analysis. The positions of the central PN, the mean, and the median center of the PNs’ distribution are partly overlapping in single-cultured slices obtained from *reln* ^(-/-)^ mice. In single-cultured slices from *reln* ^(+/-)^ heterozygous mice, it is notable that the central PN is far from the mean and median center of the distribution of the PNs and that the two centers have moved away from each other.

### Spatial Autocorrelation (Global Moran’s I) analysis

**Figure 11.**
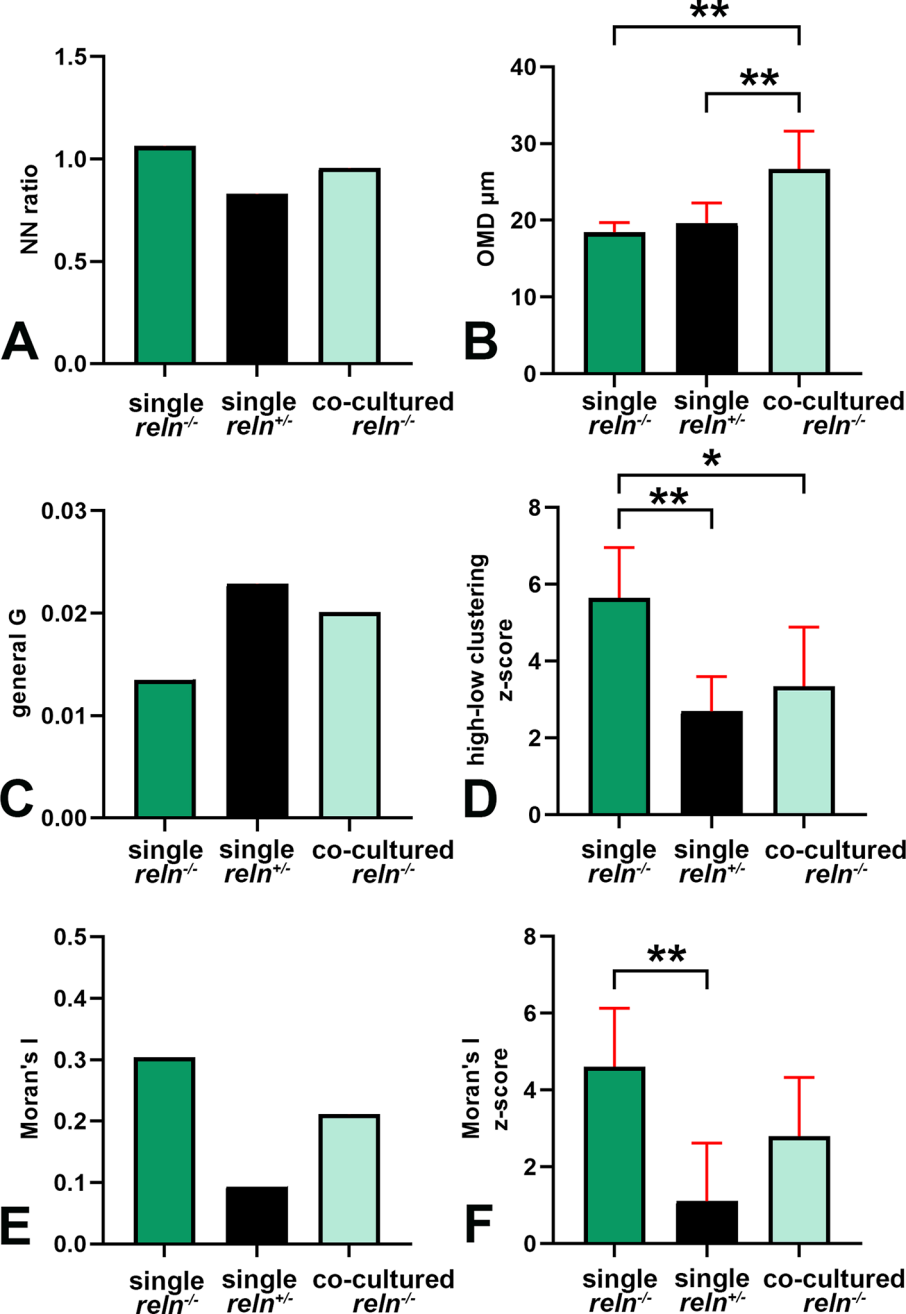
Descriptive and inferential statistics of pattern analysis with GIS. A: The mean NN ratio is different between single-cultured *reln* ^(-/-)^ slices, which display a pattern of clustering (R=1.06), and *reln* ^(+/-)^, which tend to dispersion (R=0.83). For co-cultured *reln* ^(-/-)^ slices, R is 0.96. B: OMD among the PNs under different culturing conditions. Ordinary one-way ANOVA (F = 9.028; P value = 0.0014). C: Mean values of the general G index in the three experimental groups of cultures. The index is the highest in the single cultured slices from *reln* ^(+/-)^ mice and the lowest in single cultured slices from *reln* ^(-/-)^ animals. Note that in co-cultured slices from *reln* ^(-/-)^ mice, the index is very close to that of the single-cultured slices from *reln* ^(+/-)^ mice. D: Mean z-score values of general G statistics in the three experimental groups of cultures. Ordinary one-way ANOVA with multiple comparisons (F = 7.463; P value = 0.0034). E: The mean Global Morans I is different for the three experimental groups of slices. In all three experimental groups, there is a tendency to cluster as the index is positive. The index is the highest in the single cultured slices from *reln* ^(-/-)^ mice (more clustering) and the lowest in single cultured slices from *reln* ^(+/-)^ animals (less clustering). Note that in co-cultured slices from *reln* ^(-/-)^ mice, the index is smaller than that calculated from single-cultured slices from *reln* ^(-/-)^ mice. F: Z-scores under different culturing conditions. Ordinary one-way ANOVA with multiple comparisons (F = 6.924; P value = 0.0047). Bars in inferential statistics panels B, D, and F are 95% CI.

Tables 4 (tool applied without standardization of data) and 5 (tool applied with row standardization of data) in *Supplementary Material 6* ^46^ show the statistical data of Spatial Autocorrelation (Global Moran’s I) analysis. The results of the analysis show that PNs have a clustered distribution in all three experimental groups of cultures ( Figure 11E), and the degree of clustering (measured from the z-score values associated with the statistic) is the highest in single-cultured slices obtained from homozygous *Reeler reln* ^(-/-)^ mice and the lowest in single-cultured slices obtained from heterozygous *reln* ^(+/-)^ mice Figure 11F).

### Multi-Distance Spatial Cluster Analysis (Ripley’s K Function)

The graphs of the K-functions in the three experimental groups of cultures are shown in *Supplementary Material 6* ^46^ (Figures S6-1, S6-2 and S6-3). Observation of the K-functions shows that single-cultured slices obtained from *reln* ^(-/-)^ mice (Figure S6-1), show a statistically significant clustering of the PNs at distances from 20 to 115 μm (slice 2), 180 μm (slice 4), or 200 μm (remaining slices). In single-cultured slices obtained from *reln* ^(+/-)^ mice (Figure S6-2), clustering occurs in a shorter range of distance from 20 to 100-110 μm (slices 1-3) to a maximum of about 160 μm (slices 4, 6-8). Finally, in slices from *reln* ^(-/-)^ mice co-cultured with slices from *reln* ^(+/-)^ mice (Figure S6-2), clustering is more variable within a very short maximum distance (about 60-70 μm) in slices 4-5.

In summary, the results of spatial pattern analysis demonstrate that the PNs: 1. are clustered in all three experimental groups of cultures; 2. form high-number clusters in all experimental conditions with the degree of clustering being the highest in single-cultured slices from homozygous *reln* ^(-/-)^ mice, intermediate in co-cultures of slices from homozygous *reln* ^(-/-)^ and heterozygous *reln* ^(+/-)^ mice and the lowest in single-cultured slices from heterozygous *reln* ^(+/-)^ mice (Getis-Ord General G); 3. are clustered independently from distance up to 200 μm. Note that this distance value well fits with the radius of the standard distance circle as reported in the legend of Figure 10D- F.

This pattern is similarly observed in slices from *reln* ^(-/-)^ homozygous mice co-cultured with slices from *reln* ^(+/-)^ heterozygous mice. Remarkably, the standard deviational ellipses in the three experimental groups show diverse spatial trends in the distribution of the PNs regarding the main direction of their spreading (see X-Y axes in each panel of Figure 10D- F). Also, the differences in the standard distance circles demonstrate the highest degrees of spreading of the PNS in single-cultured slices from *reln* ^(+/-)^ heterozygous mice.

### Spatial pattern analysis (see also *Supplementary Material 5* ^40^ and *6* ^46^)

#### Average Nearest Neighbor Analysis

Table 1 in *Supplementary Material 6* ^46^ shows the statistical data of the Average Nearest Neighbor (ANN) analysis. The nearest neighbor (NN) ratio (R) is different between single cultures obtained from heterozygous *reln* ^(+/-)^ and homozygous *reln* ^(-/-)^ mice, but slices from homozygous *reln* ^(-/-)^ mice in co-cultures did not display statistically significant differences with the other two groups ( Figure 11A). The observed mean distance (OMD) among the PNs in slices from homozygous *reln* ^(-/-)^ mice in co-cultures is higher than in single cultures, irrespective of the genotype ( Figure 11B).

#### High/Low Clustering (Getis-Ord General G) Analysis

Tables 2 (tool applied without standardization of data) and 3 (tool applied with row standardization of data) in *Supplementary Material 6* ^46^ show the statistical data of the Getis-Ord General G analysis. Inspection of both Tables shows that all single-cultured *reln* ^(-/-)^ slices display high clusters, and only one single-cultured *reln* ^(+/-)^ slice, as well as two co-cultured *reln* ^(+/-)^ slices, display a random distribution of the PNs. Figure 11C shows that the mean values for the G index in the three experimental groups are very close to 0 which is indicative of clustering. Given that the areas of analysis (microscopic field/magnification) have been maintained unchanged for all slices, it is possible to apply inferential statistics to compare the z-scores among the three experimental groups of cultures. Thus, the degree of clustering, measured from the z-score values associated with the statistics, is higher in single-cultured slices obtained from homozygous *reln* ^(-/-)^ mice compared to the two other groups, whereas there is not a statistically significant difference between the z-scores observed in single-cultured *reln* ^(+/-)^ slices or *reln* ^(-/-)^ slices co-cultured with *reln* ^(+/-)^ slices ( Figure 11D).

### Cluster distribution analysis (see also *Supplementary Material 5* ^40^ and *6* ^46^)

As PNs had different degrees of clustering in the three experimental groups of slices, we have used a more specific set of tools to analyze the spatial distribution of the clusters aiming to obtain additional information about the pattern of migration of the cultured PNs and to disclose other differences among the conditions under study.

***Cluster and Outlier Analysis (Anselin Local Moran’s I) and Optimized Outliers Analysis***

**Figure 12.**
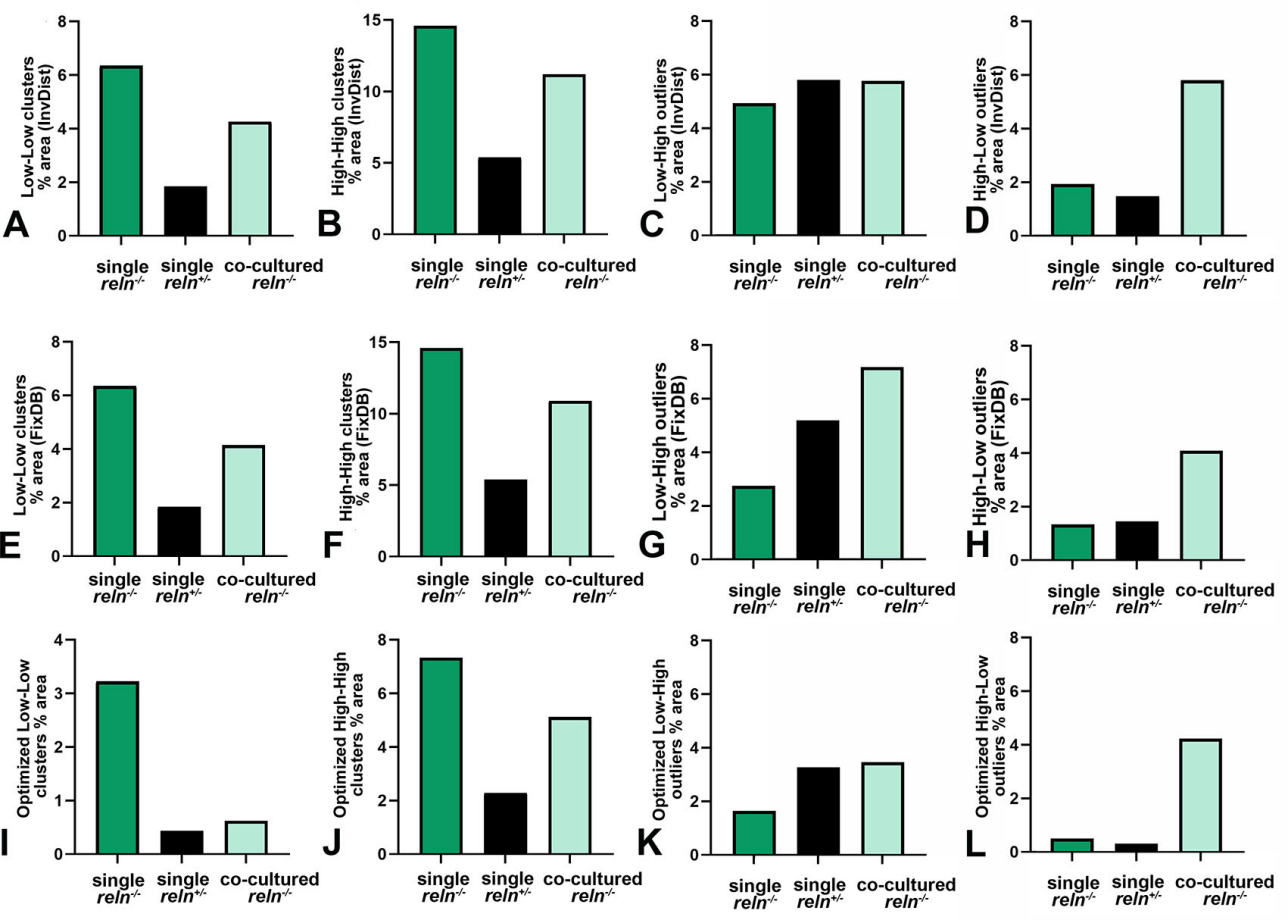
Cluster and Outlier Analysis (Anselin Local Moran's I) and Optimized Outliers Analysis. A-D: Cluster and Outlier Analysis using the inverse distance with row correction as a conceptualization of the spatial relationship between the PNs. E-H: Cluster and Outlier Analysis using the fixed distance band as a conceptualization of the spatial relationship between the PNs. I-L: Optimized Cluster and Outliers Analysis. See *Supplementary Material 6* ^46^ for further information on the principles of distance conceptualization. Percentages of the areas occupied by the clusters/outliers were calculated from the ratio of the number of hexagons color-coded as low-low clusters (light blue), high-high clusters (pink), low-high outliers (blue), and high-low outliers (red) (Figures S6-4 to S6-9) to the total number of hexagons of the tessellation.

These two tools identify spatial clusters of high or low numbers of PNs. The tools also identify spatial outliers. Computations are made using the Anselin Local Moran’s I statistic. ^47^ The difference between the two tools is that the optimized tool uses the parameters derived directly from input data to yield optimal results (see *Supplementary Material 5* ^40^ and *6* ^46^). Figure 10J- L shows some exemplificative images of the output of the Cluster and Outlier Analysis (Anselin Local Moran’s I) tool. From these images, it is evident that in single-cultured slices from homozygous *reln* ^(-/-)^ mice ( Figure 10J), there are two large areas of high-high clusters with a very high concentration of the PNs, whereas at the periphery there are areas where the PNs are highly dispersed (low-low clusters). The elaboration confirms statistically that the central mass of the PNs in this slice is split into two almost entirely separate parts ( Figure 10A). In single-cultured slices from heterozygous *reln* ^(+/-)^ mice ( Figure 10K), the PNs have for the most a random distribution (not significant) with a few areas where low and high numbers of PNs are spatially clustered (low-high outliers) and one area in which a high number of PNs is surrounded by other sparse PNs (high-low outlier). The output of the elaboration reflects the separation of the PNs into three discrete layers, each made by several sheets of neurons ( Figure 10B, E, and H) that are not yet organized to form the typical monolayer of the mature cerebellar cortex. Remarkably, the distribution of clusters in the slice from a homozygous *reln* ^(-/-)^ mouse co-cultured with slices from heterozygous *reln* ^(+/-)^ mice ( Figure 10L) does not display statistically significant local differences in the degree of PN clustering, reflecting the quite homogenous dispersion of the PNs that can be observed in Figure 10C, F, and I. This shows statistically that in *reln* ^(-/-)^ co-cultured slices, the central mass of the PNs spreads in a centrifugal direction quite uniformly along the axes of the directional ellipse of Figure 10I. We have also calculated the percentage of areas occupied by high-high clusters, low-low clusters, low-high outliers, and high-low outliers to compare their mean values among the three experimental groups ( Figure 12).

It is worth noting that, irrespective of the type of analysis, the percentages of low-low clusters and high-high clusters were the highest in single-cultured slices from homozygous *reln* ^-/-^ mice, the lowest in single-cultured slices from heterozygous *reln* ^+/-^ mice, with co-cultured *reln* ^-/-^ slices displaying intermediate values. The differences among experimental groups for the low-high outliers ( Figure 12C, G, and K) are of a less clear interpretation but it seems that the highest values are observed in co-cultured *reln* ^-/-^ slices ( Figure 12 G, and K). Similarly, this group of cultures displays the highest values in the percentage area occupied by high-low outliers ( Figure 12D, H, and L).

Hot Spot Analysis (Getis-Ord Gi*) and Optimized Hot Spot Analysis

**Figure 13.**
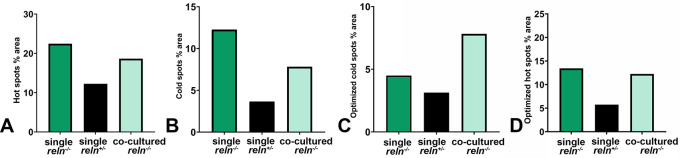
Hot Spot Analysis (Getis-Ord Gi*) and Optimized Hot Spot Analysis. Percentages of the section areas occupied by hot spots (A) and cold spots (B) after Hot Spot Analysis, and percentages of the section areas occupied by hot spots (C) and cold spots (D) after Optimized Hot Spot Analysis. E-H: Percentages were calculated from the ratio of the number of hexagons color-coded as hot or cold spot spots (Figures S6-10 to S6-15) to the total number of hexagons of the tessellation. All spots were considered independently of the differences in confidence intervals.

The Hot Spot Analysis tool and the Optimized Hot Spot Analysis tool calculate the Getis-Ord Gi* statistic ^41^ for PN localization. The resultant z-scores and p-values show where PNs in high or low numbers cluster spatially. These tools take into consideration each feature within the context of neighboring features. The difference between the two tools is that the optimized tool uses the parameters derived directly from input data to yield optimal results. Figure 10M- O shows some exemplificative images where it is clear that in single-cultured slices from homozygous *reln* ^(-/-)^ mice ( Figure 10M), there are two quite large hot spots (90-99% confidence) with a very high concentration of the PNs within the central mass. The localization of these two hot spots corresponds to that of the high-high clusters detected with Anselin’s local Moran statistic ( Figure 10J). Likewise, the 95% confidence cold spots ( Figure 10M) have a localization that overlaps with that of low-low clusters in Figure 10J. In single-cultured slices from heterozygous *reln* ^+/ -^ mice ( Figure 10N), the PNs form a few hot spots with only 90% confidence, demonstrating the dispersion of these neurons in their migration route to eventually form the typical PN layer of the mature cerebellar cortex. Remarkably, the distribution of the hot spots in the slice from a homozygous *reln* ^-/-^ mouse co-cultured with slices from heterozygous *reln* ^+/-^ mice is confined to the central area, similarly to the localization observed in the single-cultured slice from a *reln* ^+/-^ mouse ( Figure 10M), but there are fewer cold-spots at the periphery. This shows statistically that in co-cultures the central mass of the PNs spreads in a centrifugal direction mainly along the X-axis of the directional ellipse of Figure 10F. For analyses with both tools, the percentage ratios of the number of hexagons color-coded as hot spots or cold spots (Figures S6-10 to S6-15) to the total number of hexagons of the tessellation were calculated ( Figure 13). It is again notable that in all analyses co-cultured *reln* ^-/-^ slices displayed intermediate values to those of the two other groups of slices.

## Discussion

We have here developed a method to study *ex vivo* the effects of Reelin on cerebellar development that also permits a reduction in the number of animals necessary for the experiments and avoids the use of the severe procedures that are required for *in vivo* longitudinal studies. As we will discuss below, such an approach has also provided a proof of concept that GIS-based spatial statistics has a very high potential in biomedical research and proved to be a valuable tool for a better comprehension of the biological function of Reelin *in vivo.* Before doing that, we will consider the technical advantages/disadvantages of our method based on its theoretical background and effectiveness in coping with the 3Rs approach.

### Advantages and limitations of the organotypic culture approach to the study of (cerebellar) neurodevelopment

The use of brain and spinal cord organotypic cultures in neuroscience studies has several advantages, but also some intrinsic limitations. We have in the past thoroughly discussed these issues. ^17^^,^^32^ Therefore, we will briefly mention some of the points most relevant to the work presented here on the cerebellum.

Some traits seen in organotypic cultures significantly differ from those found in brains that have undergone *in vivo* physiological maturation. The cerebellum has a relatively simple architecture and the developmental history of its principal cell types is well-documented. ^48^ This widespread knowledge about cerebellar development and histology was the main reason that prompted us to use cerebellar preparations in this study. It was long ago discovered that in explants from P2 animals, PNs developed cytotypically in contrast to the organotypic evolution of P6 explants. ^49^ In other words, the cultures derived from older donors are more likely to maintain their cortical cytoarchitecture, and thus we cut slices from P5-7 mouse pups.

An issue of primary importance is that the preparation of cerebellar slices is inevitably linked to the cutting of the mossy and climbing fibers, *i.e.* the axons providing the afferent input to the cerebellar cortex - see *e.g.* Figure 2F in Ref. 17. The axons of the PNs are the only axons leaving the cerebellar cortex to make synapses onto the neurons of the cerebellar and vestibular nuclei. Therefore, they are for the most spared after slice cutting and this is why the cerebellum is an ideal organotypic preparation for neurodevelopmental studies. Remarkably, in the cultures, it was shown that in the absence of excitatory neurotransmission (following the interruption of the cerebellar afferents) and brain-derived neurotrophic factor (BDNF) signaling that occurs *in vivo*, the PN dendritic tree was normal. ^50^ These findings led the authors to hypothesize that signals from the afferent fibers are of minor importance for PN dendritic development during the stage of rapid dendritic arbor growth that occurs in the first postnatal week and that an intrinsic growth program can accomplish many aspects of PN dendritic formation. Subsequent studies have shown that the gross architecture of mouse organotypic cultures that have grown *in vitro* for 23 days is remarkably similar to that of the cerebellum *in vivo* and that developmental and reconstructive changes following experimental manipulation (destruction/reconstitution of the granule cells and glia) were not dependent on neuronal activity, except for inhibitory synaptogenesis. On these premises, we cultivated our slices for 21-30 days.

That the development of inhibitory synapses may be hampered in organotypic preparations undoubtedly represents a limitation of the approach, as the PNs and the cerebellar interneurons are inhibitory cells. Similarly, the possibility that an unphysiological response to growth factors occurs is another element of caution to the use of organotypic preparations for the dissection of the maturation of specific cerebellar circuits as, broadly speaking, BDNF has a role in cell survival, migration, and synaptogenesis, ^51^ and paracrine/autocrine BDNF release and TrkB endocytosis promote polarized migration of the granule cells to the internal granular layer of the forming cerebellar cortex along the BDNF gradient. However, while BDNF has a well-established involvement in the survival and differentiation of granule cells, ^52^^,^^53^ PN research on this issue has produced mixed results. For instance, a study revealed that the survival of these neurons during the first postnatal week was unaffected by BDNF expression levels in slice cultures ^54^ as well as *in vivo*, ^55^^,^^56^ but amelioration of cell survival was observed in dissociated cultures. ^57^

While organotypic cultures of a single area of the brain are widely employed, tissue slice co-cultures, despite having long ago been developed and since then used to analyze the functional links across two (or even more) brain regions, are much less in use, much likely because of higher technical complexity - see Ref. 17, 32, and 58. Cerebellar co-cultures were previously established by adding isolated neurons ( *e.g.* granule cells) or glia to an organotypically cultured cerebellar slice, ^59^ or by co-culturing a cerebellar slice together with another slice cut through the inferior olive, to study the connections between the climbing fibers and the PNs as well as their functional responses. ^60^^,^^61^ To our knowledge, there are no examples in the literature of the use of cerebellar organotypic co-cultures from animals of different genotypes for better dissecting neurodevelopmental mechanisms as we have described here. Rather, in the past, studies on these issues relied on the more complex use of neural chimeras. ^62^ Chimeric mice were produced by aggregating 8-cell stage embryos between *Reeler* homozygotes and normal embryos - a quite complex procedure - to study cerebellar histogenesis and led the authors to suggest that Reelin was an important extracellular environmental signal indirectly affecting the radial glial cells during the maturation of the cerebellar cortex. Besides its relative simplicity, our method has the additional advantage of permitting the direct visualization of the GFP-tagged PNs in the alive cultures and histological preparations.

Theoretically, our approach can be used for the study of the biological activities of brain-secreted proteins other than Reelin, particularly if mutant and/or transgenic animals are available in which the protein under investigation is absent, thus making it possible to prepare co-cultures of the donor (normally expressing) and the recipient (protein-lacking) slices.

There are, however, several limitations to its generalized use. First, the source of the protein under investigation and its target should be ideally localized in the same sliced area of the brain. This is the case of Reelin which, in the cerebellar cortex, is produced by the granule cells ^63^ and acts on the migration of the PNs. ^64^ In the cerebral cortex and the hippocampus, the mechanism of action of the protein on neuronal positioning is less understood and still under debate. ^65^^,^^66^ In these two forebrain regions, the cell mispositioning resulting from the lack of Reelin is much more complex to be analyzed than in the cerebellum, as an obvious consequence of the more composite neocortical structure. Initially, the *Reeler* mutants were described to simply display an inversion of the stereotypical cortical layering, ^67^ but, in these animals, the later-born neurons exhibit a broader and irregular distribution, which is far more than just an inversion of laminar fate. ^66^^,^^68^ The fact that the distribution of several types of cortical neurons is affected by the absence of Reelin makes it more difficult to analyze the effects of the protein with our co-culture approach unless one focuses on a single neuronal population, which is, of course, a quite difficult task in the absence of (a) specific marker(s) to label such a population.

Another limitation of our method is the requirement for appropriate mutant or transgenic animals. As mentioned above, during cerebellar development BDNF is produced by the granule cells and promotes the survival/migration of these neurons by paracrine/autocrine effects. ^52^ Thus one can envisage co-culturing slices from *bdnf* ^(+/+)^ and *bdnf* ^(-/-)^ mice to study the mechanisms underlying the role of the neurotrophin in granule cell maturation. However, most *bdnf* ^(-/-)^ mice only survive a couple of weeks after birth and the survival of the slices obtained from these mice may be problematic.

### Advantages and limitations of cellular sociology and GIS spatial statistic for the validation of the co-culture model

We have used two main approaches to validate the co-culture model herein described. The first, Voronoi’s tessellation, is one of the most widely employed methods to study cell dispersion/clustering in biological systems. In neurosciences, it has been utilized for a long time to study e.g. the distribution of cortical and retinal neurons (see references in *Supplementary Material 4* ^39^ and Ref. 69). The second approach, based on the use of spatial statistics, is far less common in use but is rapidly taking over for quantifying cell neighbor relationships and related biological processes (see Ref. 30 for a very recent review). The originally developed use of GIS in geography has been widened to analyze e.g. the topography of animal dentition and certain traits in human bone. ^70^ In neurosciences, GIS spatial statistics has been applied mainly to analyze the spread and epidemics of neurodegenerative diseases such as amyotrophic lateral sclerosis, ^71^ but we have been unable to find examples of its use for the study of neuronal migration.

At the core of both Voronoi’s tessellation and GIS-based procedures is the calculation of the center of mass of the cell nuclei to obtain their X-Y coordinates in the 2D Euclidean space. Therefore, images need to be manually processed or segmented with high precision to properly identify individual cells. Numerous algorithms have been developed for the segmentation of nuclear images in the last decades. ^72^ However, issues arise when segmentation needs to be applied to nuclei that are highly clustered or when imaging thick samples, such being the case here. In this situation, manual processing can boost performance greatly, as we have discussed in *Supplementary Material 2*. ^36^ Due to the intrinsic difference between Voronoi’s and GIS approaches, it is not surprising that the amount of information that we could obtain with the latter was much higher, yet the former is simpler to manage and more intuitively understood by GIS non-specialists. Regardless of this, both methods converged to demonstrate that, in co-cultures of the slices obtained from *reln* ^(-/-)^ mouse brains, the PNs tend to modify their spatial distribution to conform with that of the normal cerebellum.

#### Voronoi tessellation

The information that we could obtain from Voronoi’s tessellation, based on the measurement of polygon areas and circularity, was that the degree of clustering of the PNs in the slices from *reln* ^(-/-)^ mice co-cultured with slices from *reln* ^(+/-)^ mice was significantly reduced in comparison to that observed in single cultures of slices from *reln* ^(-/-)^ mice. The limits of Voronoi’s tessellation built from centers of mass of the cell nuclei as it was done here have been recently discussed by comparing its results with measures extracted from direct imaging of immunostained epithelial cell membranes. ^73^ It was thus found that Voronoi’s tessellation permitted a reasonably good estimation of many of the cellular morphological properties (area, perimeter, and elongation) with an error between 10 and 15%. In addition, the tessellation predicted the mean cell area with very high accuracy. However, significant cell-size-related effects and relatively large deviations for the sub-populations of small and large cells were observed with the smallest deviations in the area estimates occurring close to the peak of the area distributions, which is the foundation for the applicability of the method. The above study was performed on cultured MDCK II cells that had an area in the range of 50-270 μm ^2^. We have previously demonstrated that the areas of the PNs in wild type and *reln* ^(+/-)^ mice ranged in size from about 400 to 450 μm ^2^ ^7^ and it is well known that this type of neurons, among the largest CNS neurons, form a homogenous population throughout the cerebellum. Therefore, the pitfalls of the Voronoi tessellation related to cell size heterogeneity do not apply to our study.

#### GIS-based procedures

GIS-based procedures rely on the possibility to work with positional data – also referred to as spatial data. Spatial data are related to geographic space, which is defined as having positional data relative to the Earth’s surface. Although the anatomical and histological features of living organisms are positional data of a non-geographic nature, it is possible to arbitrarily georeference these data using the WGS1984 Greenwich projection ^70^^,^^74^ or the Universal Transverse Mercator (UTM) system (here) which is particularly precise and useful for small maps. Using the UTM system, the central meridian defines the origin of the X-coordinates and the central parallel that of the Y-coordinates, so that images are georeferenced in the Cartesian space as a set of pairs of real numbers (X-Y coordinates) – see Figure S4-1.

Differently from Voronoi’s tessellation, which is constructed from the positions of the cell centers of mass, the tessellation that we employed in GIS-based procedures is a hexagonal regular pattern made of tiles ^2^ of the same shape and size, and its field value attribute, i.e. the number of PNs, is associated with the entire area occupied by the tile. The set of regularly spaced (and contiguous) tiles with associated (field) values is defined as a raster. The associated values represent tile values and not point values, i.e. they are not the X-Y coordinates of individual PNs. This means that the value for a tile is assumed to be valid for all locations within the tile itself. The generation of a raster from the PNs X-Y coordinates is necessary as geospatial algorithms use spatial (and spatiotemporal) data in polygonal or raster form. ^75^ A discussion about spatial data mining is beyond the purpose of this paper but we believe it useful to remember here that spatial data mining presents computational and statistical difficulties. The complexity of spatiotemporal data types and relationships, including (1) the spatial relationships among the variables, i.e., that they are not independent and identically distributed; (2) the spatial structure of errors; and (3) nonlinear interactions in feature space, makes it more challenging to extract relevant and useful patterns from spatial datasets than from conventional numeric and categorical data. Tobler’s first law of geography states that “Everything is related to everything else, but near things are more related than distant things”. ^76^ The term “spatial autocorrelation effect” in spatial statistics refers to this type of geographical dependence. When evaluating data with spatial properties, ignoring autocorrelation and assuming an identical and independent distribution may result in assumptions or models that are incorrect or inconsistent with the data set. ^77^ One can refer to Ref. 75 for a more in-depth discussion.

Therefore, several additional pieces of information on the 2D distribution of the PNs were obtained by GIS due to its intrinsic difference from conventional inferential statisctics. Before discussing them, is worth briefly mentioning here the debate related to the appropriateness of comparing among groups the indexes (NN ratio R, general G, and Moran’s I) of the spatial pattern analysis (which are calculated by a spatial statistic algorithm) with the use of descriptive statistic ( Figure 11a, C, E) rather than inferential statistics. A good paper discusses this issue and can be found at ( https://mpra.ub.uni-muenchen.de/29780/1/Ballinger_Clint._Why_inferential_statistics_are_inappropriate_for_development_studies.pdf).

The first additional piece of information that we gathered by the use of geographic distribution tools, is the center, compactness, or orientation of the PN populations in the different experimental contexts of this study. Through this group of tools, we could demonstrate the differences in the positions of the central PN, mean, and median centers of the PN populations, as well as in the directional ellipses modeling their routes of migration ( Figure 10). These observations permitted statistically accurate modeling of the spreading of the PNs in their attempt to organize themselves into the monolayer of the mature cerebellar cortex.

Second, using a toolset to analyze the general pattern of the spatial distribution of the PNs (ANN, High/Low Clustering, Spatial Autocorrelation, and Multi-distance Spatial Cluster Analysis) we confirmed the existence of differences in the clustering of these neurons among the three experimental groups. Although ANN analysis can also be performed starting from Voronoi’s tessellation, we used GIS ( Figure 11A) because there are concerns that the number of neighbors suffers from systematic or size-dependent errors in the former. ^37^ In co-cultured slices from *reln* ^(-/-)^ mice, the NN index was in between that of single-cultured slices from the *reln* ^(-/-)^ mutants (>1) or from *reln* ^(+/-)^ heterozygous mice (<1). The biological significance of the NN index is difficult to interpret as values in the three groups of slices are all very close to 1 (perfect randomness). The ANN method is very sensitive to the area under study (small changes in the area parameter value can result in considerable changes in the z-score and p-value results). Therefore, identical feature distributions can result to be dispersed or clustered depending on the study area specified (see https://pro.arcgis.com/en/pro-app/latest/tool-reference/spatial-statistics/average-nearest-neighbor.htm). In our analysis we did not specify a value for the study area size and thus used the default value proposed by the tool, i.e. the minimum enclosing rectangle that would encompass all PNs in each analyzed image. An inconvenience of this is that the presence of isolated cells at the periphery of the microscopic field deeply influences the size of the study area. Rather than the NN index itself, the OMD was more informative ( Figure 11B): the high value of the OMD in co-cultured *reln* ^*(-/-*)^ slices reflects the attempt of the PNs to spread from the central mass towards the nascent PCL where they tend to get organized into a more or less regular monolayer. The High/Low Clustering (Getis-Ord General G) and the Spatial Autocorrelation (Global Moran’s I) tools measure different spatial patterns. With both tools, the p-values of most slices were statistically significant, and the z-scores were positive (see Tables 2-5 in *Supplementary Material 6*). Therefore, the High/Low Clustering analysis demonstrated that the spatial arrangement of the high clusters of PNs in the dataset exhibited a higher degree of spatial clustering than what would be anticipated under the assumption of complete spatial randomness in the underlying spatial processes. On the other hand, the Spatial Autocorrelation analysis showed that the dataset had a spatial distribution characterized by a higher degree of spatial clustering for both high and low values, which deviated from what would be anticipated if the underlying spatial processes were random. Using the High/Low Clustering tool, we have demonstrated that PN high-clustering was the most represented type of pattern in all three experimental groups of slices (see Tables 2 and 3 in *Supplementary Material 6*) with a grouping of values higher than average as the General G index was >0. Descriptive statistics of the mean values of the index in the three experimental groups of slices ( Figure 11C) showed that in the co-cultured slices from *reln* ^(-/-)^ mice, the mean value of the index was close to that observed in slices from *reln* ^(+/-)^ heterozygous mice. The z-scores calculated by the tool can be compared among areas if the study areas and parameters used for the analyses are the same as is the case of our microscopic fields (see https://pro.arcgis.com/en/pro-app/latest/tool-reference/spatial-statistics/h-how-high-low-clustering-getis-ord-general-g-spat.htm), thus we have used Ordinary one-way ANOVA with multiple comparisons to estimate the differences among the three experimental groups of slices ( Figure 11D) and, also with this type of analysis, observed that co-cultured slices from *reln* ^(-/-)^ mice assumed a spatial distribution close to that of the slices from *reln* ^(+/-)^ heterozygous mice. Similar conclusions could be drawn also from the results of Spatial Autocorrelation analysis ( Figure 11E-F). Finally, the Multi-Distance Spatial Cluster analysis has demonstrated that PNs were clustered independently from distances up to 200 μm (see Figures S61-3 in *Supplementary Material 6* ^46^).

A last series of information was obtained using Cluster and Outlier Analysis (Anselin Local Moran’s I) and Hot Spot Analysis (Getis-Ord Gi*). These tools give information about the local distribution of different types of PN clusters and both their manual and optimized configurations showed that in all three experimental groups of slices, it was possible to identify inhomogeneities in the forms of low-low clusters, high-high clusters, low-high outliers, and high-low outliers (Figures S6-4 to 9 in *Supplementary material 6* ^46^) as well as hot (clustering) and cold (dispersion) spots (Figures S6-10 to 15 in *Supplementary material 6* ^46^) in the distribution of the PNs. Remarkably, when we compared the relative areas occupied by the different types of PN aggregations among the slices of different experimental groups, we again observed, consistently with all previous types of analyses, that the co-cultured slices from *reln* ^(-/-)^ mice shifted toward the spatial configuration typical of the single cultured slices from *reln* ^(+/-)^ heterozygous mice ( Figures 12 and 13).

To our knowledge, little use of GIS has been done up to now in the field of neurobiology. An interesting example is the construction of an interactive developmental atlas for the study of the worm *C. elegans*, ^78^ but this study not related to the present discussion.

### Insights on Reelin function in neurodevelopment

The *reln* ^(-/-)^ mouse brain is atrophic upon gross morphological inspection with a volumetric reduction by roughly 19% in comparison to normal mice. ^79^ This size loss is most noticeable in the cerebellum, which additionally shows very little foliation. Rather than a change in cell fate determination or axonal guidance, the histological defects in *Reeler* mutants are primarily caused by an aberrant neuronal migration resulting in an ectopic position of diverse neuronal populations. Among other irregularities, the cerebral and cerebellar cortices as well as the hippocampus lose their layered structure, and multiple neuronal nuclei are lost or hardly discernible in numerous brain regions (see Table 2 in Ref. 11).

In this study, we decided to focus on the cerebellum because it is more severely affected by the mutation than other areas of the brain and thus we believed it was easier to prove or disprove the usefulness of our approach. The absence of alignment of the PNs to form a distinct intermediate layer in the cerebellar cortex is perhaps the most apparent histological trait in *Reeler* mutants. Thus, only 10% of the PNs in these animals are still inside the cortex but in the granular layer, the remaining 85% remain entrapped into an internal cellular mass mixed with the white matter, and only around 5% are in a normal position. ^4^^–^^6^

Although the structural effects of the *Reeler* mutation are well known, there are two interesting issues related to our present work that deserve further discussion. The first is the still-existing uncertainty on the mechanisms of migration of the PNs during development. Reelin has been proven to intervene in the correct layering of the cerebellar cortex beyond any reasonable doubt and the molecule also has a role in the development of the Bergmann glia that may also regulate the formation of the PN monolayer. ^80^ However, several other actors may play important parts in the process by which the PNs disperse from their clustered stage (normally between E18-P3 in mouse) to their final position in a monolayer and the main explanations that have been put forward to explain this event, i.e. the surface expansion of the cerebellum, the development of the mature granule cells, and the guidance by the Bergmann glia remain a question of debate. ^81^ Initially, the clustered PNs accumulate into the mantle zone to form the cerebellar plate (or Purkinje cell plate) but then the plate expands and forms several clusters of PNs that are well apparent around E17.5. ^82^ It appears that no migration is occurring at this time, only a slight displacement of PN groups as a result of the expanding cerebellum. After the creation of the clusters, the second wave of PN migration/displacement begins, between P3-P13 in mice: in parallel with the cerebellar enlargement, the clusters spread, form a single monolayer with uniform spacing, and simultaneously develop axons and dendrites. ^83^ Our present GIS-based results on the local distribution of the different types of PN clusters give spatial statistics confirmation of the above observational studies. In addition, they suggest that heterogeneity in cluster types reflects the existence of multiple mechanisms to govern PN alignment in the mature cerebellum. Coming back to the pivotal role of Reelin in cerebellar development, little work has been done on the possibility of modifying Reelin-mediated deficits in live cells. A study in *Reeler*-normal chimeric mice has previously demonstrated that the morphology and position of the cerebellar neurons and glial cells are controlled by extracellular environments but not genotypes. ^84^ Subsequent work has shown that the protein produced by cortical explants of *reln* ^(+/+)^ or *reln* ^(+/-)^ mice co-cultured with a *Reeler*-like ferret dysplastic cortex was capable of at least partly rescuing neuronal migration in the ferret explant. ^85^ Other studies, were carried out in the cerebral cortex and hippocampus and led to similar conclusions. It was thus shown that cortical layer development and orientation are modulated by relative contributions of Reelin-negative and -positive neurons in mouse chimeras ^86^ and that the possibility to restore a normal phenotype in *reln* ^(-/-)^ slices co-cultured with wild-type slices was mediated via the Reelin receptors apolipoprotein E receptor 2 (ApoER2) and very-low-density lipoprotein receptor (VLDLR). ^87^ Our present observations are in full accord with these studies. A point left open for discussion regards the Reelin dosage necessary for the rescue of a normal phenotype in culture studies. In their chimeric mouse study, Yoshiki and Kusakabe qualitatively observed that only a few granule cells derived from a normal mouse were capable of promoting the alignment and the proper dendritic tree development of the PNs derived from the mutant. From their observations, these authors suggested that Reelin secreted from a single granule cell can affect the morphology of PNs in a wide area. ^84^

Our results quantitatively demonstrate that the Reelin produced by the slices obtained from haplodeficient mice is sufficient to restore an almost normal cerebellar phenotype. Therefore, our findings reinforce the idea that it may be possible to modify Reelin-mediated deficits through the experimental administration of the protein. Experiments have demonstrated that the medium obtained from dissociated cerebellar cultures from P5-8 normal mice as well as from cell extracts of the cultured cerebellum from these mice contained full-length Reelin. ^88^ These observations are fully in line with the demonstration that the protein is produced by the postmitotic granule cells of the deep external granular layer before they migrate to their final destination, the internal granular layer, where they lose Reelin immunoreactivity. ^89^

We have focused our attention on the migration of the PNs for the reasons explained previously in this section. Yet our approach could be useful to investigate the function of Reelin in the formation of dendritic spines. ^10^ In the hippocampus, the Reelin signaling pathway was demonstrated to promote dendritic spine development, ^90^ as acute Reelin injection affects dendritic spine shape but not the spine density of live adult mice. ^91^ On the other hand, Reelin injection *in vivo* for a five-day interval similarly enhances spine density. ^92^ Together, these results indicate that subthreshold levels of Reelin are harmful because they impede the growth of excitatory synapses normally, but chronically high levels of Reelin may also be harmful because they change the shape and amount of spines. Considering the temporal evolution of the external granular layer in the postnatal cerebellum ^93^ we can safely infer that Reelin is produced by the cerebellar slices originating from *reln* ^(+/-)^ mice for the entire length of our experiments and from a merely qualitative inspection of our preparations that the PN dendritic arbor in *reln* ^(-/-)^ slices co-cultured with slices from heterozygous *reln* ^(+/-)^ mice has a normal morphology. Additional studies will be required to establish whether or not there is a full or partial functional recovery in parallel with the reconstitution of the cortical layering, as from P5 onward, ultrastructural observations on the *Reeler* mouse revealed a decrease in the density of connections between the PNs dendrites and the parallel and climbing fibers. ^94^ Irrespective of their ectopic or orthotopic position in the cerebellar cortex, *Reeler* PNs showed a 0–1 response to stimulation, indicating that, like in healthy mice, they were synaptically connected to a single climbing fiber. Instead, because many climbing fibers provided them with a convergent input, the PNs in the central cellular mass demonstrated intensity-graded responses to electrical stimulation, ^4^ likely because physiological pruning did not take place. ^95^ These studies would require co-culturing the cerebellar slices in the presence of inferior olivary neurons to maintain an intact afferent input from the climbing fibers.

It would be also possible to study other sets of cerebellar cells with our technique. We have previously demonstrated that the temporal expression of various extensively distributed neuronal and glial markers (NeuN, vimentin, calbindin, GFAP, Smi32, GAD67) during postnatal development showed no noticeable differences between normal mice and the mutants. ^93^ The panel of neuronal markers used in this study demonstrated that the granule cells, as well as the molecular layer ( *i.e.* the basket and stellate cells) and granule cell layer ( *i.e.* the Golgi and Lugaro cells) GABAergic interneurons are normally detected during the Reeler postnatal development although the mispositioning of this neurons was not studied in details and without the use of spatial statistics. On the other hand, the Bergmann glia was misplaced in *Reeler* ^96^ and displayed an oblique orientation rather than being perpendicular to the pial surface of the cerebellar laminae. ^93^ Therefore, our method can in the future be used to better understand the effect of the *Reeler* mutation on these populations of neurons and the Bergmann glia, although it will be necessary to use specific markers of these cells to fully exploit our approach and the search for cell-specific markers for cerebellar neurons is still an ongoing process. ^97^

Additional information on the neurodevelopmental mechanisms regulated by Reelin would also be valuable for translational medicine. ^11^ A series of human pathologies are modeled in the *Reeler* mouse. These include the monogenic conditions provoked in humans by the RELN gene, *i.e.*, lissencephaly 2 (LIS2) and autosomal-dominant lateral temporal epilepsy (ADLTE), and a series of pathologies related to genes encoding for the proteins of the Reln intracellular cascade or only tentatively linked to RELN. ^11^

### Organotypic cultures in the 3Rs context

Our 3Rs approach could be primarily applied to neuroscience, although it is possible to envisage the preparation of slice cultures from organs such as the muscle, heart, liver, and solid tumors.

We have discussed in a previous publication the barriers for other potential end-users in the adoption of rodent *ex vivo* platforms in the field of neuroscience. ^17^ In short, the main disadvantage of organotypic cultures lies in the disconnection of the explants from other areas of the brain with interruption of afferent and/or efferent pathways. Potential solutions to address/overcome these problems should be mainly sought in the reconstruction *ex vivo* of these connections by co-cultivating areas that are physiologically connected *in vivo*. ^98^^,^^99^

We believe it is important that this or similar approaches are adopted by others, as they can be used in medium throughput screening experiments preliminary to (if necessary) true experimentation *in vivo.* They have several scientific benefits, ^32^ such as the possibility to precisely control the experimental environment, pharmacologically manipulating the system with ease, the relative facility to perform longitudinal studies, and the possibility to use several complementary techniques (genetic engineering, electrophysiology, immunocytochemistry) for biological characterization.

As partly discussed elsewhere previously, ^17^ the potential of our approach in terms of animal reduction is remarkable as one can theoretically envisage reducing the number of experimental animals to at least one-fifth when aiming to characterize a single bioactive molecule.

## Conclusions

This 3Rs approach is useful to study the effect of cellular interactions in a system modeling the *in vivo* condition but with remarkable benefits for animal reduction and refinement, avoiding the use of heavy surgery that is often necessary for the molecule(s) to reach the brain.

## Data Availability

Figshare: Voronoi analysis - Cultured Reln haplodeficient heterozygous mouse cerebellar slices, https://doi.org/10.6084/m9.figshare.21063616.v1. ^44^ This project contains the following underlying data:
• Original images and images elaborated for Voronoi analysis of cerebellar slices from postnatal day 5-7 *Reln* haplodeficient heterozygous hybrid mice (L7-GFP *reln* ^+/-^F1/) cultured for 21 days in vitro. • Area, Perimeter, and Circularity of individual Voronoi polygons. Area Disorder (AD), Roundness Factor Homogeneity (RFH), and Mean Roundness Factor (RFav) of the Voronoi polygon population. Figshare: Voronoi analysis - Cultured Reln deficient homozygous mouse cerebellar slices, https://doi.org/10.6084/m9.figshare.21063517.v1. ^43^ This project contains the following underlying data:
• Original images and images elaborated for Voronoi analysis of cerebellar slices from postnatal day 5-7 *Reln* deficient homozygous hybrid mice (L7-GFP *reln* ^-/-^F1/) cultured for 21 days in vitro. • Area, Perimeter, and Circularity of individual Voronoi polygons. Area Disorder (AD), Roundness Factor Homogeneity (RFH), and Mean Roundness Factor (RFav) of the Voronoi polygon population. Figshare: Voronoi analysis - Reln deficient homozygous mouse cerebellar slices co-cultured with Reln haplodeficient heterozygous mouse cerebellar slices, https://doi.org/10.6084/m9.figshare.21063280.v1. ^45^ This project contains the following underlying data:
• Original images and images elaborated for Voronoi analysis of cerebellar slices from postnatal day 5-7 Reln deficient homozygous hybrid mice (L7-GFP *reln* ^-/-^F1/) co-cultured for 21 days in vitro in the presence of slices from Reln haplodeficient homozygous hybrid mice (L7-GFP *reln* ^+/-^F1/). • Area, Perimeter, and Circularity of individual Voronoi polygons. Area Disorder (AD), Roundness Factor Homogeneity (RFH), and Mean Roundness Factor (RFav) of the Voronoi polygon population. Data are available under the terms of the Creative Commons Zero “No rights reserved” data waiver (CC0 1.0 Public domain dedication). Figshare: Co-cultures of cerebellar slices from mice with different reelin genetic backgrounds as a model to study cortical lamination - Supplementary Material 1, https://doi.org/10.6084/m9.figshare.24087834.v1. ^23^ This project contains the following extended data:
• Workflow of the procedure to prepare organotypic single cultures and co-cultures from mice of different *reln* backgrounds. • Images of the immunocytochemical characterization of cultures. Data are available under the terms of the Creative Commons Attribution 4.0 International (CC BY 4.0 – Attribution 4.0 International). Figshare: Co-cultures of cerebellar slices from mice with different reelin genetic backgrounds as a model to study cortical lamination - Supplementary Material 2, https://doi.org/10.6084/m9.figshare.24086940.v2. ^36^ This project contains the following extended data:
• Description of the effect of section thickness and Purkinje neurons stacking on the definition of the centers of mass of cell nuclei in Voronoi analysis (sites) and GIS-based procedures. Data are available under the terms of the Creative Commons Attribution 4.0 International (CC BY 4.0 – Attribution 4.0 International). Figshare: Co-cultures of cerebellar slices from mice with different reelin genetic backgrounds as a model to study cortical lamination - Supplementary Material 3, https://doi.org/10.6084/m9.figshare.24087861.v1. ^37^ This project contains the following extended data:
• Description of the principles at the basis of the exclusion of polygons in Voronoi analysis. Data are available under the terms of the Creative Commons Attribution 4.0 International (CC BY 4.0 – Attribution 4.0 International). Figshare: Co-cultures of cerebellar slices from mice with different reelin genetic backgrounds as a model to study cortical lamination - Supplementary Material 4, https://doi.org/10.6084/m9.figshare.24087870.v1. ^39^ This project contains the following extended data:
• Analysis of the comparative features of some Voronoi generators that can be used for the study of cellular sociology. Data are available under the terms of the Creative Commons Attribution 4.0 International (CC BY 4.0 – Attribution 4.0 International). Figshare: Co-cultures of cerebellar slices from mice with different reelin genetic backgrounds as a model to study cortical lamination - Supplementary Material 5, https://doi.org/10.6084/m9.figshare.24087891.v1. ^40^ This project contains the following extended data:
• Brief introduction to spatial statistics method for use in neuroscience research. Data are available under the terms of the Creative Commons Attribution 4.0 International (CC BY 4.0 – Attribution 4.0 International). Figshare: Co-cultures of cerebellar slices from mice with different reelin genetic backgrounds as a model to study cortical lamination - Supplementary Material 6, https://doi.org/10.6084/m9.figshare.24087915.v1. ^46^ This project contains the following extended data:
• Data on the spatial pattern distribution of the Purkinje neurons in cerebellar slices explanted from mice with different reelin genetic backgrounds. Data are available under the terms of the Creative Commons Attribution 4.0 International (CC BY 4.0 – Attribution 4.0 International). Figshare: ARRIVE checklist for ‘Co-cultures of cerebellar slices from mice with different *reelin* genetic backgrounds as a model to study cortical lamination’, https://doi.org/10.6084/m9.figshare.21299211.v1. ^24^ Data are available under the terms of the Creative Commons Zero “No rights reserved” data waiver (CC0 1.0 Public domain dedication).

## Footnotes

^1^
For the sake of clarity we will avoid using the term cell to refer to Voronoi polygons.

^2^
According to the general principles of GIS, tiles can be also referred to as cells. We avoided the use of this latter term for clarity.
