# Supplementary material for: Co-cultures of cerebellar slices from mice with different reelin genetic backgrounds as a model to study cortical lamination
Source: F1000Res. 2023 Oct 2;11:1183. Originally published 2022 Oct 17. [Version 2] doi: 10.12688/f1000research.126787.2 (PMC10594056; doi:10.12688/f1000research.126787.2)
Supplement: Peer review history

# Reviewer report: Reviewer response for version 2

**DOI:** 10.5256/f1000research.155550.r211352

**Published:** 2023-10-23

**Authors:** Pascale Chavis, Thomas Boudier

**Affiliations:** 1 INMED, INSERM, Aix-Marseille University, Marseille, France; 2 CENTURI, Aix-Marseille University, Marseille, France

The authors have made appropriate revisions and have provided comprehensive responses to all of our concerns.

Is the rationale for developing the new method (or application) clearly explained?

Yes

Is the description of the method technically sound?

Yes

Are the conclusions about the method and its performance adequately supported by the findings presented in the article?

Yes

If any results are presented, are all the source data underlying the results available to ensure full reproducibility?

Yes

Are sufficient details provided to allow replication of the method development and its use by others?

Yes

Reviewer Expertise:

Pascale Chavis: Brain plasticity, Reelin neurobiology, Neuropsychiatric diseases. Thomas Boudier: Image processing, Image analysis.

We confirm that we have read this submission and believe that we have an appropriate level of expertise to confirm that it is of an acceptable scientific standard.

# Reviewer report: Reviewer response for version 2

**DOI:** 10.5256/f1000research.155550.r211353

**Published:** 2023-10-17

**Authors:** Hector J. Caruncho, Brady Reive

**Affiliations:** 1 Division of Medical Sciences, University of Victoria, Victoria, BC, Canada; 2 University of Victoria, Victoria, BC, Canada

I consider the revisions made by the authors as appropriate and recommend the article be considered as 'Approved'.

Is the rationale for developing the new method (or application) clearly explained?

Yes

Is the description of the method technically sound?

Yes

Are the conclusions about the method and its performance adequately supported by the findings presented in the article?

Yes

If any results are presented, are all the source data underlying the results available to ensure full reproducibility?

Yes

Are sufficient details provided to allow replication of the method development and its use by others?

Yes

Reviewer Expertise:

NA

We confirm that we have read this submission and believe that we have an appropriate level of expertise to confirm that it is of an acceptable scientific standard.

# Reviewer report: Reviewer response for version 1

**DOI:** 10.5256/f1000research.139230.r153857

**Published:** 2022-11-18

**Authors:** Pascale Chavis, Thomas Boudier

**Affiliations:** 1 INMED, INSERM, Aix-Marseille University, Marseille, France; 2 CENTURI, Aix-Marseille University, Marseille, France

**Summary:**

 The study reports the implementation of a co-culture method to analyze the effects of reelin and reduce animal use within the 3R framework. Hybrid mice were generated by crossing L7-GFP with heterozygous reeler to obtain GFP-tagged Purkinje neurons in different reelin backgrounds. Co-cultures of cerebellar slices from homozygous and heterozygous hybrids resulted in dispersion and morphology changes of GFP-tagged Purkinje neurons derived from slices bearing a homozygous background.

**General:**

 The main concern is about the general scope of the paper and whether it is about ethical use of animals in biological experiments and animal welfare or about reelin neurobiology in the 3R framework. Although the two former issues are not to be overlooked, they should be developed in the discussion rather than in the introduction, highlights and results.

 The authors also state that this approach will allow the study of reelin effects on cortical lamination and that the method will also be applicable to investigate the function of other brain secreted proteins. They should show that this is indeed the case either in co-cultures of other cortices from reelin hybrids or in cerebellar co-cultures from mutants of different proteins.

**Strengths:**

 The authors developed an elegant technique highly suited for longitudinal and dynamic studies, implemented in the 3Rs framework. They provide a very detailed and exhaustive description of the method to allow replication in other labs.

**Major points:**

**Methods:**

*Voronoi analysis:* The authors perform Voronoi analysis to analyze spatial organization of cells. Although they give references to support their work, the theoretical paper is dated from 1992.  Since then, framework and tools to study spatial organization, based on robust spatial statistics such as F, G, H and K functions, have been developed (see Lagache *et al.*, 2013 ^1^ for example).

 The overall method is also extremely manual, involving at least 4 different softwares, and the initial cell detection is purely manual, along with the Voronoi adjustment. The removal of Voronoi zones of cells touching edges is justified, but no explanation is given on why the authors chose to favor convex area hence removing some additional cells in their study.

*Quantitative analysis of PN migration:* since slices expand with time (Figure 1), how is the center point precisely localized through different time points?

**Data interpretation:**

 In co-cultures, changes in the histology of *reln-/-* slices are interpreted as resulting from the effect of Reelin produced by *reln+/-* slices. However, there is no clear demonstration of this, as this could be due to another secreted factor. To answer this point, reelin blockade (function or binding, etc...) should be achieved. This experiment would also have the advantage to validate the statement made in the highlight that co-cultures are amenable to pharmacological intervention or other type of manipulation/treatment.

 In the same line of thought, one can wonder whether *reln-/-* slices phenotypical improvement would be better achieved if the donor mice were *reln+/+* instead of *reln+/-?* This would validate the method further and also give an indication about time and/or dose effect.

**Statistics:**

 In Figure 7, what is the variable: does each point represent one culture? How many polygons are included in each point, this could actually be a bias in condition where GFP-PN survival decrease with time such as in the *reln-/-* group.

 Please, indicate in legend samples size i.e. number of cultures/dishes used.

 In 7B, the distribution in the single *reln+/-*, clearly shows 2 groups in Voronoi polygons areas. Could you comment? Moreover, this kind of distribution undermines the use of means as presented in 7C.

**Figures:**

 Figures 1 and 5 are somehow overlapping. The paper would gain clarity by showing in different figures temporal modifications and histological characterization of single cultures and co-cultures (similar to Figure 2).

**Minor points:**

 What is the age of the mice used for slice preparation? And why are mice euthanized using sodium pentobarbital prior brain slicing (protocols.io.6qpvr67bbvmk/v1)?

 What is the survival time of the single and co-cultures?

Is the rationale for developing the new method (or application) clearly explained?

Yes

Is the description of the method technically sound?

Yes

Are the conclusions about the method and its performance adequately supported by the findings presented in the article?

Yes

If any results are presented, are all the source data underlying the results available to ensure full reproducibility?

Yes

Are sufficient details provided to allow replication of the method development and its use by others?

Yes

Reviewer Expertise:

Pascale Chavis : Brain plasticity, Reelin neurobiology, Neuropsychiatric diseases. Thomas Boudier : Image processing, Image analysis.

We confirm that we have read this submission and believe that we have an appropriate level of expertise to confirm that it is of an acceptable scientific standard, however we have significant reservations, as outlined above.

## References

[1] : Analysis of the spatial organization of molecules with robust statistics. *PLoS One* .2013;8(12) : 10.1371/journal.pone.0080914 e80914 10.1371/journal.pone.0080914 24349021PMC3857798

# Response

**Published:** 2023-09-04

**Authors:** Adalberto Merighi

**Affiliations:** University of Turin, Italy

**Before responding point-to-point to the reviewers’ observations that as a whole were very useful and supportive to ameliorate our work, we allow ourselves to put beforehand here a brief history of the paper submission and its main purposes. We initially wrote this paper as a Brief Report in response to the NC3Rs center's call for papers written by the NC3Rs grantees to be published in their Gateway of F1000 Research. According to the call rules, papers were subjected to the Center’s check for their suitability for submission according to the mission of the organization. After this check, not only the paper was approved to be eligible for APC funding, but we were asked to expand it in the form of a full Method Paper, as it was then submitted to the journal.**

**We believe this is the reason why it ended up displaying some mix between its focus on the 3Rs principles and the neurobiology issues that could be raised according to its results. We are grateful to the reviewers who spotted this primary fault of the original manuscript and we have done our best to correct it.**

**Having said so, we allow ourselves to stress that some of the requests made by the referees (use of cultures from wild-type mice, extension of the study to other areas of the brain, block of Reelin ex vivo) would have indeed required to produce a significant additional amount of work that could be hardly considered within the NC3Rs framework. This is because the Reeler mouse is a suffering phenotype (at least according to Italian animal welfare regulations) and we were not in the position to ask for additional simply confirmative experiments using these mice. Namely, as we have discussed in the section of the Discussion of the revised paper entitled “Insights on Reelin function in Neurodevelopment” several other papers have been published in which the block of the protein function in vitro resulted in at least a partial rescue of the normal cerebellar phenotype, as we have shown here. Moreover, the study of other areas of the brain affected by the Reeler mutation was beyond our original purpose, and the use of the approach here proposed (as discussed in response to this specific comment below) would have required the generation of a specific mouse strain with some sort of useful fluorescent tagging of the cell population of putative interest.**

**As all the above comments were surely aimed at strengthening the soundness of the method that we have proposed, we decided to pursue a different approach to validate the original Voronoi analysis and employed a series of spatial statistic tools that, up to now, are rarely used in neurobiology to analyze the dispersions of the Purkinje neurons in our cultures. Not only did these tools confirm the results of the Voronoi analysis, but gave interesting additional information on the mechanisms of migration of the Purkinje neurons during postnatal cerebellar development.**

**Thus, we are confident that the reviewers will share our point of view and support the publication of the paper in its thoroughly revised form.**

 Summary:

 The study reports the implementation of a co-culture method to analyze the effects of reelin and reduce animal use within the 3R framework. Hybrid mice were generated by crossing L7-GFP with heterozygous reeler to obtain GFP-tagged Purkinje neurons in different reelin backgrounds. Co-cultures of cerebellar slices from homozygous and heterozygous hybrids resulted in dispersion and morphology changes of GFP-tagged Purkinje neurons derived from slices bearing a homozygous background.

 General:

 The main concern is about the general scope of the paper and whether it is about ethical use of animals in biological experiments and animal welfare or about reelin neurobiology in the 3R framework. Although the two former issues are not to be overlooked, they should be developed in the discussion rather than in the introduction, highlights and results.

**We fully understand this concern. A similar concern was also raised by Drs. Caruncho and Reive. The reason why the issues of the ethical use of animals in biological experiments and animal welfare have been developed in different parts of the manuscript other than the Discussion is to cope with the guidelines issued by the NC3Rs for Method papers. In response, we have fully revised the paper according to these observations as explained above in the response to the comments of the other two reviewers and in the preamble to our responses.**

 The authors also state that this approach will allow the study of reelin effects on cortical lamination and that the method will also be applicable to investigate the function of other brain secreted proteins. They should show that this is indeed the case either in co-cultures of other cortices from reelin hybrids or in cerebellar co-cultures from mutants of different proteins.

**We agree with the reviewers that the experiments suggested would be indeed useful to confirm that our approach will permit studying 1. The effects of reelin in the other laminated areas of the brain or 2. The function of other secreted proteins. Yet, as regards the first issue, we have now thoroughly discussed previous experiments with approaches different from the one used here demonstrating the effects of Reelin on the lamination of the cerebral cortex. As regards the second issue, we have now softened our claims on the possible applications of our method to the study of other secreted brain proteins in another specific section of the discussion (Advantages and limitations of the organotypic culture approach to the study of neurodevelopment). We have also considered the issues raised here in a more thorough discussion of the strengths and weaknesses of our approach, as such a discussion was also suggested by Drs. Drs. Caruncho and Reive.**

**We are confident that the reviewers would agree with us that the experiments suggested would require a large amount of work that could the the subject of another paper and well beyond the purpose of this Method Paper (that as mentioned in our prologue was sponsored by the NC3Rs and was prepared according to the guidelines for the NC3Rs gateway). Apart from this, there are several theoretical and practical limitations to the possibility of performing the experiments above (we have addressed this point in response to the first comment under “Data Interpretation”).**

**That reelin produced by the cerebellar granule cells in slices from heterozygous or homozygous Reeler mice can diffuse in the culture medium was previously demonstrated after studies on chimeric mice and in Reeler cortical cultures. We have addressed this issue in the Discussion of the revised paper as follows:**

*Although the structural effects of the Reeler mutation are well known, little work has been done on the possibility of modifying Reelin-mediated deficits in live cells. A study in the Reeler-normal chimeric mice has previously demonstrated that the morphology and position of the cerebellar neurons and glial cells are controlled by extracellular environments but not by their genotype* *[11]* *.  Subsequent work has shown that the protein produced by cortical explants of reln ^+/+^ or reln ^+/-^ mice co-cultured with a Reeler-like ferret dysplastic cortex was capable of at least partly rescuing neuronal migration in the ferret explant* *[12]* *. Other studies, were carried out in the cerebral cortex and hippocampus and led to similar conclusions. It was thus shown that cortical layer development and orientation are modulated by relative contributions of Reelin-negative and -positive neurons in mouse chimeras* *[13]* *and that the possibility to restore a normal phenotype in reln ^-/- ^slices co-cultured with wild-type slices was mediated via the Reelin receptors apolipoprotein E receptor 2 (ApoER2) and very-low-density lipoprotein receptor (VLDLR)* *[14]* *. Our present observations are in full accord with these studies. A point left open for discussion regards the Reelin dosage necessary for the rescue of a normal phenotype in culture studies. In their chimeric mouse study, Yoshiki and Kusakabe qualitatively observed that only a few granule cells derived from a normal mouse were capable of promoting the alignment and the proper dendritic tree development of the PNs derived from the mutant.  From their observations, these authors suggested that Reelin secreted from a single granule cell can affect the morphology of PNs in a wide area* *[11]* *.*

*Our results quantitatively demonstrate that the Reelin produced by the slices obtained from haplodeficient mice is sufficient to restore the normal cerebellar phenotype. Therefore, our findings reinforce the idea that it may be possible to modify Reelin-mediated deficits through the experimental administration of the protein.  Experiments have demonstrated that the medium obtained from cerebellar dissociated cultures from P5-8 normal mice as well as from cell extracts of the cultured cerebellum from these mice contained full-length Reelin* *[15]* *. These observations are fully in line with the demonstration that the protein is produced by the postmitotic granule cells of the deep external granular layer before they migrate to their final destination, the internal granular layer, where they lose Reelin immunoreactivity* *[16]* *.*

 Strengths:

 The authors developed an elegant technique highly suited for longitudinal and dynamic studies, implemented in the 3Rs framework. They provide a very detailed and exhaustive description of the method to allow replication in other labs.

 Major points:

 Methods:

 Voronoi analysis: The authors perform Voronoi analysis to analyze spatial organization of cells. Although they give references to support their work, the theoretical paper is dated from 1992.  Since then, framework and tools to study spatial organization, based on robust spatial statistics such as F, G, H and K functions, have been developed (see Lagache et al., 20131 for example).

**Frankly, we do not see why Voronoi's analysis should be hampered by its relative age. Voronoi tesselation has been and still is widely employed in the study of the spatial organization of many diverse types of neuronal (and other) cell populations** **[17]** **.**

**The paper quoted by the reviewers uses a modified Ripley’s K function to define the clustered, dispersed, or uniform distribution of points (i.e. clathrin endocytotic sites) in the 2D space** **[18]** **. Ripley's K-, H-, and L-functions are used primarily to identify the clustering of proteins in membrane microdomains (as is indeed the case for Lagache’s paper). Using this approach, aggregation (or clustering) is identified if the average number of proteins within a distance r of another protein is statistically greater than that expected for a random distribution** **[19]** **. Kiskowski et al. emphasized that “it is not entirely clear how the function may be used to quantitatively determine the size of domains in which clustering occurs” and have widely discussed the limitations and potential of Ripley's K in real-life scenarios”** **[19]** . In the case of this study, where indeed the main issue under investigation is the clustering of the PNs, which is very high in the reln ^-/- ^mice, one can raise reasonable doubts about the usefulness of applying Ripley's K in our conditions. Despite the above observations on the limitations of Ripley’s functions to modeling spatial clustering we have added a large bunch of new analyses based on the novel use of spatial statistics with ArcMap [geographical distribution tools (Central Feature, Mean Center, Median Center, Directional Distribution, and Standard Distance), pattern distribution tools (Average Nearest Neighbor, Getis-Ord General G, Ripley’s K function, and Global Moran’s I), and mapping clusters tools (Anselin Local Moran’s I, and Getis-Ord G*)] to compare their results with the conclusions that could be drawn solely from Voronoi analysis. As thoroughly discussed in the revised paper the two approaches yielded comparable results in support of our initial claims on the soundness of the method.

 The overall method is also extremely manual, involving at least 4 different softwares, and the initial cell detection is purely manual, along with the Voronoi adjustment.

**We have already at least partly addressed this comment in response to Drs. Caruncho and Reive observations (see Supplementary Material 4). In addition, we would like to stress here that the possibility of using an automated procedure is severely hampered by the extremely high compactness of the central mass of the PNs lying inside the cerebellar white matter. In other words, it seems to be very difficult to practically separate the individual PNs with a thresholding procedure based on color or gray histograms (see Fig. S4-2). In addition, the aforementioned paper by Lagache and collaborators uses a similar manual procedure for the initial detection of the regions of interest (ROIs) as reported in their Materials and Methods: “We first delimited cells’ contours by drawing polygonal Region of Interest (ROIs) with the Icy software [13] (http://icy.bioimageanalysis.org). We then used a wavelet-based detection method [28], implemented as a plugin Spot detector in Icy to extract the two-dimensional positions of putative endocytic spots at the cellular membrane…”.**

 The removal of Voronoi zones of cells touching edges is justified, but no explanation is given on why the authors chose to favor convex area hence removing some additional cells in their study.

**We have addressed this comment also in response to similar observations made by Drs. Caruncho and Reive (see main text and Supplementary Material 3 and 4).**

 Quantitative analysis of PN migration: since slices expand with time (Figure 1), how is the center point precisely localized through different time points?

**We believe that this comment applies to Figure 2 and thus refers to qualitative analysis. The center point was recorded using the memory system of the microscope stage at 4 DIV. Subsequent images were recorded using the memorized X-Y coordinates.**

 Data interpretation:

 In co-cultures, changes in the histology of reln-/- slices are interpreted as resulting from the effect of Reelin produced by reln+/- slices. However, there is no clear demonstration of this, as this could be due to another secreted factor. To answer this point, reelin blockade (function or binding, etc...) should be achieved. This experiment would also have the advantage to validate the statement made in the highlight that co-cultures are amenable to pharmacological intervention or other type of manipulation/treatment.

**We agree that, theoretically, the changes in the histology of co-cultured reln ^-/- ^slice could be dependent on a different secreted factor. However, several published papers support our line of thought as we have discussed in the revised version of the paper (reported above). To our knowledge, no other molecules except Reelin and its downstream receptors and adaptor molecules have been demonstrated to be primarily responsible for the correct layered organization of the cerebellar cortex during postnatal development of altricial mammals, although the true mechanisms of PNs layering in the mature cerebellar cortex remain a matter of debate** **[20]** **. As authoritatively reviewed by Gabriella D’Arcangelo** **[21]** **, the discoverer of Reelin and certainly one of the leading scientists in the field “In the cerebellum, Reelin is produced by granule cells in the external granule layer and is essential for the radial migration of the Purkinje neurons, which are born in the cerebellar ventricular zone and form at first a plate and then a single-cell layer. This assessment derives from a widespread analysis of cerebellar development in Reeler mice** **[2, 22-24]** **, from Reelin gene and protein expression data** **[16, 25]** **, and functional studies in organotypic cultures** **[26]** **”.**

**We likewise agree that the blockade of Reelin would have the advantage of validating our statement that co-cultures are amenable to pharmacological or other types of manipulation. However, this would require a huge amount of additional work that is prone to several worries about its practicability (e.g. the effectiveness of Reelin-blocking antibodies -when available -to penetrate the entire thickness of a slice). In addition, much of the results that may be stemming from these efforts would be mainly confirmatory of previous observations. We have now discussed in our revision the previous literature on the block of the Reelin function in cultured cerebellar slices that demonstrates a long-distance effect of the molecule in vitro.**

 In the same line of thought, one can wonder whether reln-/- slices phenotypical improvement would be better achieved if the donor mice were reln+/+ instead of reln+/-? This would validate the method further and also give an indication about time and/or dose effect.

**We agree with this observation in theory, but previously published observations on mouse chimeras** **[11]** **led to the conclusion that very little Reelin production could be sufficient to rescue the Reeler phenotype. In addition, our main goal was to validate the co-culture method that will be surely of good use to answer these questions in future work.**

 Statistics:

 In Figure 7, what is the variable: does each point represent one culture? How many polygons are included in each point, this could actually be a bias in condition where GFP-PN survival decrease with time such as in the reln-/- group.

**The variable in the figure (now Figure 9) is a randomly chosen culture slice. The number of polygons included in each point is indicated in FigShare uploaded Excel files (.xlsx or .csv). Inspection of these files shows that the number of polygons remains very high in single cultured reln ^-/-^ slices despite survival decrease thus excluding a bias.**

 Please, indicate in legend samples size i.e. number of cultures/dishes used.

**Done**

 In 7B, the distribution in the single reln+/-, clearly shows 2 groups in Voronoi polygons areas. Could you comment? Moreover, this kind of distribution undermines the use of means as presented in 7C.

**The existence of two groups of Voronoi polygon areas is explained by the layering of the cortex in the slices from heterozygous mice. We respectfully disagree with the observation that this kind of distribution undermines the use of the means in the now Figure 7C as 1. Analysis was supported by inferential statistics and 2. Results from ArcMap GIS analysis where the tessellations of images only included the hexagons with PNs inside (see Figures 6 and 10 in main text and Figs. S64-15 in Supplementary Material 6) led to fully comparable results as shown and discussed in the revised version of the paper.**

 Figures:

 Figures 1 and 5 are somehow overlapping. The paper would gain clarity by showing in different figures temporal modifications and histological characterization of single cultures and co-cultures (similar to Figure 2).

**In response, we have deleted panels A and B in the original Figure 1. In addition, we have shown other images of the cultures to better demonstrate their histological appearance (Fig. 10A-C) and Figure S2.**

 Minor points:

 What is the age of the mice used for slice preparation? And why are mice euthanized using sodium pentobarbital prior brain slicing (protocols.io.6qpvr67bbvmk/v1)?

**The mice were 5 days old. We have added this information to the revised paper. They were euthanized with sodium pentobarbital (SP) as routinely done in our laboratory. Injectable barbiturates are acceptable for use in mouse fetuses and neonates according to the AVMA GUIDELINES FOR THE EUTHANASIA OF ANIMALS: 2020 EDITION, whereas decapitation and cervical dislocation are acceptable with conditions according to the guidelines. In addition, a recent publication** **[27]** **has reviewed the effects of SP (and other euthanasia methods) on cells and tissues as well as on different analytes without finding adverse effects of SP versus decapitation and/or cervical dislocation in the brain. Therefore we prefer to use SP also following the recommendations of our Department’s Ethical Committee.**

 What is the survival time of the single and co-cultures?

**The survival time was 30 days at maximum. We have added this information to the revised paper.**

 References (for both responses to reviewers)

 1             Jossin, Y.: ‘Reelin Functions, Mechanisms of Action and Signaling Pathways During Brain Development and Maturation’, Biomolecules, 2020, 10, (6)

 2             Mariani, J., Crepel, F., Mikoshiba, K., Changeux, J.P., and Sotelo, C.: ‘Anatomical, Physiological and Biochemical Studies of the Cerebellum from Reeler Mutant Mouse’, Philosophical Transactions of the Royal Society of London B: Biological Sciences, 1977, 281, (978), pp. 1-28

 3             Magliaro, C., Cocito, C., Bagatella, S., Merighi, A., Ahluwalia, A., and Lossi, L.: ‘The number of Purkinje neurons and their topology in the cerebellar vermis of normal and reln haplodeficient mouse’, Ann Anat, 2016, 207, pp. 68-75

 4             Ruiz i Altaba, A., Palma, V., and Dahmane, N.: ‘Hedgehog–GLI signaling and the growth of the brain’, Nature Reviews Neuroscience, 2002, 3, (1), pp. 24-33

 5             Cocito, C., Merighi, A., Giacobini, M., and Lossi, L.: ‘Alterations of Cell Proliferation and Apoptosis in the Hypoplastic Reeler Cerebellum’, Frontiers in Cellular Neuroscience, 2016, 10, pp. 141

 6             Oberdick, J., Levinthal, F., and Levinthal, C.: ‘A Purkinje cell differentiation marker shows a partial DNA sequence homology to the cellular sis/PDGF2 gene’, Neuron., 1988, 1, (5), pp. 367-376

 7             Nordquist, D., Kozak, C., and Orr, H.: ‘cDNA cloning and characterization of three genes uniquely expressed in cerebellum by Purkinje neurons’, The Journal of Neuroscience, 1988, 8, (12), pp. 4780-4789

 8             Sługocka, A., Wiaderkiewicz, J., and Barski, J.J.: ‘Genetic Targeting in Cerebellar Purkinje Cells: an Update’, Cerebellum, 2017, 16, (1), pp. 191-202

 9             Maskos, U., Kissa, K., St Cloment, C., and Brûlet, P.: ‘Retrograde trans-synaptic transfer of green fluorescent protein allows the genetic mapping of neuronal circuits in transgenic mice’, Proc Natl Acad Sci U S A, 2002, 99, (15), pp. 10120-10125

 10           Preparata, F.P., and Shamos, M.: ‘Computational Geometry: An Introduction’ (Springer New York, 1993. 1993)

 11           Yoshiki, A., and Kusakabe, M.: ‘Cerebellar histogenesis as seen in identified cells of normal-reeler mouse chimeras’, Int.J Dev Biol., 1998, 42, (5), pp. 695-700

 12           Schaefer, A., Poluch, S., and Juliano, S.: ‘Reelin is essential for neuronal migration but not for radial glial elongation in neonatal ferret cortex’, Dev Neurobiol, 2008, 68, (5), pp. 590-604

 13           Hammond, V.E., So, E., Cate, H.S., Britto, J.M., Gunnersen, J.M., and Tan, S.S.: ‘Cortical layer development and orientation is modulated by relative contributions of reelin-negative and -positive neurons in mouse chimeras’, Cereb Cortex., 2010, 20, (9), pp. 2017-2026

 14           Zhao, S., Chai, X., Bock, H.H., Brunne, B., Förster, E., and Frotscher, M.: ‘Rescue of the reeler phenotype in the dentate gyrus by wild-type coculture is mediated by lipoprotein receptors for Reelin and Disabled 1’, J Comp Neurol, 2006, 495, (1), pp. 1-9

 15           D’Arcangelo, G., Nakajima, K., Miyata, T., Ogawa, M., Mikoshiba, K., and Curran, T.: ‘Reelin Is a Secreted Glycoprotein Recognized by the CR-50 Monoclonal Antibody’, The Journal of Neuroscience, 1997, 17, (1), pp. 23-31

 16           Miyata, T., Nakajima, K., Aruga, J., Takahashi, S., Ikenaka, K., Mikoshiba, K., and Ogawa, M.: ‘Distribution of a reeler gene-related antigen in the developing cerebellum: An immunohistochemical study with an allogeneic antibody CR-50 on normal and reeler mice’, Journal of Comparative Neurology, 1996, 372, (2), pp. 215-228

 17           Galli-Resta, L., Novelli, E., Kryger, Z., Jacobs, G.H., and Reese, B.E.: ‘Modelling the mosaic organization of rod and cone photoreceptors with a minimal-spacing rule’, European Journal of Neuroscience, 1999, 11, (4), pp. 1461-1469

 18           Lagache, T., Lang, G., Sauvonnet, N., and Olivo-Marin, J.C.: ‘Analysis of the spatial organization of molecules with robust statistics’, PLoS One, 2013, 8, (12), pp. e80914

 19           Kiskowski, M.A., Hancock, J.F., and Kenworthy, A.K.: ‘On the use of Ripley's K-function and its derivatives to analyze domain size’, Biophys J, 2009, 97, (4), pp. 1095-1103

 20           Rahimi-Balaei, M., Bergen, H., Kong, J., and Marzban, H.: ‘Neuronal Migration During Development of the Cerebellum’, Frontiers in Cellular Neuroscience, 2018, 12

 21           D'Arcangelo, G.: ‘Reelin in the Years: Controlling Neuronal Migration and Maturation in the Mammalian Brain’, Advances in Neuroscience, 2014, vol. 2014, Article ID 597395, 19 pages. doi:10.1155/2014/597395

 22           Goffinet, A.M.: ‘The embryonic development of the cerebellum in normal and reeler mutant mice’, Anat Embryol (Berl), 1983, 168, (1), pp. 73-86

 23           Mikoshiba, K., Nagaike, K., Kohsaka, S., Takamatsu, K., Aoki, E., and Tsukada, Y.: ‘Developmental studies on the cerebellum from reeler mutant mouse in vivo and in vitro’, Developmental Biology, 1980, 79, (1), pp. 64-80

 24           Inoue, Y., Maeda, N., Kokubun, T., Takayama, C., Inoue, K., Terashima, T., and Mikoshiba, K.: ‘Architecture of Purkinje cells of the reeler mutant mouse observed by immunohistochemistry for the inositol 1,4,5-trisphosphate receptor protein P400’, Neuroscience Research, 1990, 8, (3), pp. 189-201

 25           D'Arcangelo, G., Miao, G.G., Chen, S.C., Soares, H.D., Morgan, J.I., and Curran, T.: ‘A protein related to extracellular matrix proteins deleted in the mouse mutant reeler’, Nature, 1995, 374, (6524), pp. 719-723

 26           Miyata, T., Nakajima, K., Mikoshiba, K., and Ogawa, M.: ‘Regulation of Purkinje Cell Alignment by Reelin as Revealed with CR-50 Antibody’, The Journal of Neuroscience, 1997, 17, (10), pp. 3599-3609

 27           Shomer, N.H., Allen-Worthington, K.H., Hickman, D.L., Jonnalagadda, M., Newsome, J.T., Slate, A.R., Valentine, H., Williams, A.M., and Wilkinson, M.: ‘Review of Rodent Euthanasia Methods’, Journal of the American Association for Laboratory Animal Science, 2020, 59, (3), pp. 242-253

# Reviewer report: Reviewer response for version 1

**DOI:** 10.5256/f1000research.139230.r153860

**Published:** 2022-11-03

**Authors:** Hector J. Caruncho, Brady Reive

**Affiliations:** 1 Division of Medical Sciences, University of Victoria, Victoria, BC, Canada; 2 University of Victoria, Victoria, BC, Canada

**Summary of study:**

 Hybrid mice were generated by crossing male heterozygous Reelin mutants with L7-GFP females, resulting in both homozygous and heterozygous Reeler hybrids with GFP-tagged Purkinje neurons. Cerebellum slices from both groups were cultured and co-cultured together. The morphology and clustering of cultured cells were compared and found differences to cell morphology and organization between homozygous Reeler mutant cultures versus co-cultures also including heterozygous Reeler tissue.

**Strengths and weaknesses:**

**Strengths: **Interesting original work. Low animal numbers needed. Relatively simple yet elegant combination of techniques. Thorough descriptions of methods to help readers apply to their own purposes/replication.

**Weaknesses:**

**General: **A bit strange that the conclusion is drawn pertaining to potential applications of cell cultures in general (I say that because although they’re not intended, it seems to speak to most forms of cell culture rather than specific to this technique). If this is a ground-breaking technique, it might be more valuable to show that it can be applied to more than one protein. Many proteins relate to cell survival, regulation of signaling, etc. as opposed to cell migration, so it’s a bit difficult to understand how this technique would benefit the study of other aspects of cell function. I suppose part of the issue for me is the absence of discussion of limitations to the model, so as I am imagining all sorts of limitations without the authors discussion or recognition of them, I’m left not understanding if/how the limitations would be explained. Some other limitations include: can you conclude that application of Reelin would resolve the layering deficits in homozygous Reelers through this model? I’m not understanding how the authors conclude cells originating from the homozygous slice show improved organization in co-cultures, as opposed to showing that amongst the co-culture, the cells originating from the heterozygous Reeler are the only cells showing altered organization because you a) can’t verify which slice they originate (both reported with the same protein, i.e. GFP, which can also be exchanged between cells); b) there’s no change from heterozygous and co-cultures, supporting the “Occam’s razor” hypothesis that it’s really a comparison of heterozygous cells against other heterozygous cells. Maybe it wasn’t clear enough how co-cultures are grown together to allow for differentiation.

**General comments on:**

**Methods: **Good overall. My only concerns: Polygon selection and removal of the top polygon, which corresponds to the culmination of data (or all accounted for polygons). I’m not understanding why that information wouldn’t be valuable for comparing sets of polygons (I understand it’s included de facto in the sum of all selected polygons) but it would be good to show the amalgamations are similar across cultures (i.e. I would expect less stacking/layering of cells occurs in smaller montages of polygons compared to bigger ones corresponding to larger numbers of cells, which would impact nearest neighbour analyses etc.). Verification that the initial selection of cells is accurate is not done, and in fact is explained as being negligible. “ Using the mouse, click above the center of each cell to generate the Voronoi polygons. Due to the thickness of the slice, it may be possible that two very close cells in the Z axis are not easily distinguished. This introduces an error that can be neglected considering that all slices are cut at the same initial thickness.”

 It isn’t clear why initial thickness of section would dictate how one would treat stacking of grown cells. The stacking isn’t uniform just because the initial slice was uniform (at least it very much appears there exists more stacking in the central mass relative to the periphery. Also, it matters entirely because those clicks define the cell sizes, which is one of the outputs for statistical comparisons.

 The inability to verify which slice cells originate should be described/explained. Is it possible to use a secondary reporter to compare and contrast within co-cultures? What would it mean that you can’t verify if so? 

 Is it true the imaging is done at the central mass? I had a difficult time understanding because there is also a section that states the coordinates images could be taken at. I’m very curious if there’s an impact of image location/bias involved. Is the origin selected by the researcher, are there methods for ensuring similarly sized clusters of cells are being imaged? Is it randomized? How many cells are removed in each?

 I found this comment particularly interesting: “ **It is possible to use several other Voronoi generators that can be found online as freeware or in dedicated programs**.” They acknowledge the existence of other modeling systems, would be valuable to know if the results are similar., i.e. the impact of the modeling choice. Even a visual demonstration to compare the resulting polygons would be a powerful addition (to answer the obvious question about validity of Voronoi method, how important is accurate cell selection, is there an impact of user experience, etc.).

**Data interpretation: **I think my biggest concern is lack of description of limitations/how perceivable limitations might be overcome, and secondary, that the discussion and conclusion only includes the word Reelin once, in the first sentence…otherwise the paper is about animal welfare? If the focus is going to be about how to reduce animal sizes, more information should be provided about litter sizes, culling, survival of mutants, etc. and how litter survival might be a limitation for studying other proteins using this method (or not). More information would be required about how many cultures can be derived from a single animal (i.e. size of slice versus size of structure at age of collection, etc.) Either it’s about Reelin, which I think it should be, and I think is interesting, or it’s a methods paper, in which case I think it would be much more valuable to show positive results in at least 2 proteins of interest in a way that allows for comparing the cultures to really show the applications of this technique. I think it might be limited to studying proteins with a very specific subset of functions, so maybe choosing another protein with a vastly different function of Reelin would be a good choice to really highlight the value of this technique. Instead, I think re-writing the discussion and conclusion to focus on results about Reelin would be more appropriate. What can the authors infer about the possibility of Reelin application to modify Reelin-mediated deficits? What can the authors infer about Reelin’s function in regulating cell morphology, beyond simple layering/migration results? Are these results consistent with the in vivo known functions of Reelin? What does this mean? Would you expect there would be differences in non-Purkinje cells? What other sets of cells you can study with this technique? Are there basket cells, granular cells etc. in the culture that we can’t visualize? Why the cerebellum? Is there interaction between the various cells in culture (both PN to PN and PN to any other non-GFP cells)? How might you evaluate cell interactions using this model in the future? What other directions would you go? Just so much to talk about and instead we were told we can save an undetermined, unspecified number of animals…but to learn what? Instead of providing 2 highly specific proteins that could be studied with this method, why not provide a sweeping generalized set of proteins you might have interest in studying with this technique? Usually, the issue is people over-exclaim their success, but in this case, I think not enough effort is given to really dive into what this study indicates about Reelin or what else can be done with the method. If this method is so great, then there should be no shortage of things to tell the reader that you learned about Reelin in applying the technique, instead of how many fewer animals might be needed in future hypothetical studies.

**Language: **Good language and writing. No considerable issues noted (grammar, spelling, awkward word choice, etc.).

**Figures and Tables: **Very thorough and good quality figures and figure descriptions. No issues.

**Statistics: **Only issue with stats described earlier: pertaining to non-randomized imaging location (coordinates in the dish essentially) leads to selection bias. Alternative suggestions would include: a) create a grid and take an average of all polygons in multiple grid boxes to get a variety of polygon origins within the analysis. Additionally, it would be valuable to demonstrate whether or not peripheral polygons are truly similar to the central mass related polygons, in other words, a within culture analysis would also be valuable (is there more or less homogeneity of cultures from Reelin homo- or heterozygous mice?). Combining a perceived selection bias with unconfirmed cell selection, the reader might reasonably be concerned the results aren’t the most meaningful or generalizable.

**Ethics: **No concerns.

Is the rationale for developing the new method (or application) clearly explained?

Yes

Is the description of the method technically sound?

Yes

Are the conclusions about the method and its performance adequately supported by the findings presented in the article?

Yes

If any results are presented, are all the source data underlying the results available to ensure full reproducibility?

Yes

Are sufficient details provided to allow replication of the method development and its use by others?

Yes

Reviewer Expertise:

Neuropharmacology; Reelin neurobiology; Animal models; Mood and Psychotic disorders

We confirm that we have read this submission and believe that we have an appropriate level of expertise to confirm that it is of an acceptable scientific standard, however we have significant reservations, as outlined above.

# Response

**Published:** 2023-09-04

**Authors:** Adalberto Merighi

**Affiliations:** University of Turin, Italy

**Before responding point-to-point to the reviewers’ observations that as a whole were very useful and supportive to ameliorate our work, we allow ourselves to put beforehand here a brief history of the paper submission and its main purposes. We initially wrote this paper as a Brief Report in response to the NC3Rs center's call for papers written by the NC3Rs grantees to be published in their Gateway of F1000 Research. According to the call rules, papers were subjected to the Center’s check for their suitability for submission according to the mission of the organization. After this check, not only the paper was approved to be eligible for APC funding, but we were asked to expand it in the form of a full Method Paper, as it was then submitted to the journal.**

**We believe this is the reason why it ended up displaying some mix between its focus on the 3Rs principles and the neurobiology issues that could be raised according to its results. We are grateful to the reviewers who spotted this primary fault of the original manuscript and we have done our best to correct it.**

**Having said so, we allow ourselves to stress that some of the requests made by the referees (use of cultures from wild-type mice, extension of the study to other areas of the brain, block of Reelin ex vivo) would have indeed required to produce a significant additional amount of work that could be hardly considered within the NC3Rs framework. This is because the Reeler mouse is a suffering phenotype (at least according to Italian animal welfare regulations) and we were not in the position to ask for additional simply confirmative experiments using these mice. Namely, as we have discussed in the section of the Discussion of the revised paper entitled “Insights on Reelin function in Neurodevelopment” several other papers have been published in which the block of the protein function in vitro resulted in at least a partial rescue of the normal cerebellar phenotype, as we have shown here. Moreover, the study of other areas of the brain affected by the Reeler mutation was beyond our original purpose, and the use of the approach here proposed (as discussed in response to this specific comment below) would have required the generation of a specific mouse strain with some sort of useful fluorescent tagging of the cell population of putative interest.**

**As all the above comments were surely aimed at strengthening the soundness of the method that we have proposed, we decided to pursue a different approach to validate the original Voronoi analysis and employed a series of spatial statistic tools that, up to now, are rarely used in neurobiology to analyze the dispersions of the Purkinje neurons in our cultures. Not only did these tools confirm the results of the Voronoi analysis, but gave interesting additional information on the mechanisms of migration of the Purkinje neurons during postnatal cerebellar development.**

**Thus, we are confident that the reviewers will share our point of view and support the publication of the paper in its thoroughly revised form.**

 Weaknesses:

 General: A bit strange that the conclusion is drawn pertaining to potential applications of cell cultures in general (I say that because although they’re not intended, it seems to speak to most forms of cell culture rather than specific to this technique). If this is a ground-breaking technique, it might be more valuable to show that it can be applied to more than one protein. Many proteins relate to cell survival, regulation of signaling, etc. as opposed to cell migration, so it’s a bit difficult to understand how this technique would benefit the study of other aspects of cell function. I suppose part of the issue for me is the absence of discussion of limitations to the model, so as I am imagining all sorts of limitations without the authors discussion or recognition of them, I’m left not understanding if/how the limitations would be explained.

**We thank the reviewers for these very useful observations. We have revised the paper by adding a thorough discussion of the limitations of the technique and trying to avoid a too strong generalization of its applicability.**

 Some other limitations include: Can you conclude that application of Reelin would resolve the layering deficits in homozygous Reelers through this model? I’m not understanding how the authors conclude cells originating from the homozygous slice show improved organization in co-cultures, as opposed to showing that amongst the co-culture, the cells originating from the heterozygous Reeler are the only cells showing altered organization because you a) can’t verify which slice they originate (both reported with the same protein, i.e. GFP, which can also be exchanged between cells); b) there’s no change from heterozygous and co-cultures, supporting the “Occam’s razor” hypothesis that it’s really a comparison of heterozygous cells against other heterozygous cells. Maybe it wasn’t clear enough how co-cultures are grown together to allow for differentiation.

**We are sorry that we have not been able to clarify these issues in the original version of the paper. We believe that, from our observations, we can indeed conclude that the reelin secreted from the cerebellar granule cells in the slices from heterozygous mice (reelin haplodeficient) has a positive effect on the organization of the cerebellar cortex in the slices obtained from homozygous mice (lacking reelin).**

**Our belief is based on the following:**

1. **Faulty Reelin signaling in the cerebellum causes Purkinje neurons (PN) migration to be disrupted and, as a result, granule cell precursor (GCPs) proliferation to be diminished – for a recent review see** **[1]** **. Morphological observations in vivo have long ago demonstrated that the Reeler mouse, which is devoid of Reelin, displays an altered stratification of its cerebellar and cerebral cortices, as well as of other layered areas of the brain. Specifically in the cerebellum, these alterations affect the migration and correct positioning of the PNs that, in the homozygous reln ^-/-^ mouse, remain clustered together in a deep cellular mass embedded into the medullary body of the white matter** **[2]** **. Conversely, in reln ^+/- ^and reln ^+/+^ mice the PNs migrate in an outward direction to eventually form a monolayer of cells between the molecular and granule cell layer of the mature cerebellar cortex, i.e. both genotypes display a normal cerebellar architecture, although we have recently demonstrated that there are very subtle differences in the position of the PNs between  reln ^+/- ^and reln ^+/+^** **mice** **[3]** **. The effects of the PNs on the GCP proliferation are mediated by Sonic Hedgehog (reviewed in** **[4]** **) and we have subsequently demonstrated that the hypoplasia in the Reeler mouse cerebellum is consequent to a reduction of cortical size and cellularity, the latter being linked to quantitative differences in the extent of GCP proliferation and apoptosis between normal and Reeler mice** **[5]** **. The interactions between the PNs and the GCPs are thus quite complex: from one side the PNs stimulate the proliferation of GCPs and, from the other, the CGPs produce and release Reelin in the intercellular space, and the glycoprotein is essential for the correct positioning of the PNs.**
2. **If we understood well the reviewers’ thoughts, they suggest that we cannot claim that “**the cells originating from the homozygous slice show improved organization in co-cultures” **as it is equally possible** **“**that amongst the co-culture, the cells originating from the heterozygous Reeler are the only cells showing an altered organization” **but this in total conflict with the above-reported literature.**
3. **In addition, the reviewers believe that we are overinterpreting our findings because**:
  1. **We could not** “verify which slice they originate (both reported with the same protein, i.e. GFP, which can also be exchanged between cells” **. However, we never suggested that there was a migration of PNs from one slice to the other, and there are no pieces of evidence from previous studies that GFP is exchanged between cells in the L7 mouse. The PN-specific gene coding L7 or pcp2 (Purkinje cells specific protein-2) was first described in 1988 by two independent groups** **[6, 7]** **, and since then it has been of paramount importance for genetic targeting of these neurons** **[8]** **. Anyway, even if one assumes that the GFP can be transferred between cerebellar neurons, the protein should preferentially reach the neurons of the cerebellar nuclei which are in contact with the PN axons, as the retrograde transsynaptic transfer occurs only when a fusion protein with a non-toxic fragment of tetanus toxin is used for genetic engineering/transfection, see e.g.** **[9]** **.**
  2. “there’s no change from heterozygous and co-cultures, supporting the “Occam’s razor” hypothesis that it’s really a comparison of heterozygous cells against other heterozygous cells”. **Although Occam’s razor is based on the idea that the simplest explanation is often the best one, we don’t see how the reviewers may conclude that we are simply comparing heterozygous (PN) cells between each other, since they derive from genotyped animals and no exchange of PNs occurs among slices. On the contrary, the simplest explanation of our findings is indeed the one put forward in the original paper.**

**Yet, we fully agree with the referees that** “it wasn’t clear enough how co-cultures are grown together to allow for differentiation” **and we have amended the manuscript by better explaining how co-cultures were established and how they could influence each other by adding some supplementary information to the paper (Supplementary Material 1).**

 General comments on:

 Methods: Good overall. My only concerns: Polygon selection and removal of the top polygon, which corresponds to the culmination of data (or all accounted for polygons). I’m not understanding why that information wouldn’t be valuable for comparing sets of polygons (I understand it’s included de facto in the sum of all selected polygons) but it would be good to show the amalgamations are similar across cultures (i.e. I would expect less stacking/layering of cells occurs in smaller montages of polygons compared to bigger ones corresponding to larger numbers of cells, which would impact nearest neighbour analyses etc.).

**The principle at the basis of Voronoi** **tessellation** **is that a plane can be divided into regions close to each of a given set of objects, in our case the centers of the GFP-tagged PNs. Cell centers are mathematically referred to as sites (or seeds or generators). For each site, there is a corresponding region, called a Voronoi cell (polygon), consisting of all points of the plane closer to that seed than to any other. In our case, PNs lay on a Euclidean plane (2D) and their centers form a discrete set of points. The marginal polygons must be excluded from analysis because one or two (when in the corners of the microscope image) of their sides are indeed extending to the infinite (i.e. they are OPEN polygons) as they are defined by the perimeter of the image and NOT by the existence of another site outside the microscopic field (See Fig. S3-1 in Supplementary Material 3).**

**In other words, the software designs these polygons only because the image is a finite portion of the space and the marginal polygons do not derive from the algorithm at the basis of the** **tessellation.** **Similarly, one can explain the need to eliminate the polygons that are intersected by the convex hull (asterisks in Fig. S3-1).** **The convex hull of a finite set PP of points in the plane can be defined as the unique convex polygon whose vertices are points from PP and which contains all points of PP. It can be demonstrated that a Voronoi polygon is unbounded if and only if one of its points is on the convex hull (indicated by asterisks in the figure). As a corollary, the convex hull can be computed from the Voronoi diagram in linear time** **[10]** **. Being unbounded, the polygons intersecting the convex hull do not have a finite area and thus cannot the used in the analysis.** **We have tried to better explain these concepts in the revised paper and Supplementary Material 3. Additionally, in Supplementary Material 3 we have used a different Voronoi generator that automatically computes the convex hull (Fig. S3-2) and demonstrated that there are no statistically significant differences with our original procedure to determine the hull (Fig. S3-3).**

 Verification that the initial selection of cells is accurate is not done, and in fact is explained as being negligible. “Using the mouse, click above the center of each cell to generate the Voronoi polygons. Due to the thickness of the slice, it may be possible that two very close cells in the Z axis are not easily distinguished. This introduces an error that can be neglected considering that all slices are cut at the same initial thickness.”

 It isn’t clear why initial thickness of section would dictate how one would treat stacking of grown cells. The stacking isn’t uniform just because the initial slice was uniform (at least it very much appears there exists more stacking in the central mass relative to the periphery. Also, it matters entirely because those clicks define the cell sizes, which is one of the outputs for statistical comparisons.

**Unfortunately, we have not been clear in explaining our point regarding the position of the GFP-tagged cells along the Z-axis of the section. We agree with the referees that stacking is not uniform, but what we signified in saying that selection errors were negligible was that if two or more cells are perfectly stacked one on top of the others we could visualize and take into consideration only the cells in the plane of focus as we used a 2D (and not a 3D) Voronoi analysis. To better clarify this point we have amended the text of the revised manuscript and added explanatory information in Supplementary Material 2.**

 The inability to verify which slice cells originate should be described/explained. Is it possible to use a secondary reporter to compare and contrast within co-cultures? What would it mean that you can’t verify if so? 

**If we understand well this comment signifies (also according to the comment above about the impossibility of verifying the origin of the cells as a consequence of a theoretical exchange of GFP from cell to cell) that we could not identify with certainty the phenotype of the GFP-tagged PNs in slices. We have substantially rebutted this possibility in responding to the previous comment. In addition, we don't see the need for a secondary reporter to compare and contrast within co-cultures. In the original paper, although not strictly necessary, we used calbindin 28k (a well-established marker of the PNs) to unambiguously prove the nature of the GFP-tagged neurons and we obtained a 100% coexistence of both tags (see e.g. Fig. 1 where the fluorescence of the positive neurons is yellow/orange for the sum of the green fluorescence of GFP and the red fluorescence of calbindin-immunostaining). This said, we believe is 100% positive that we could verify the origin of the cells in individual slices. To make this clearer, we have better explained how we have seeded the slices in the culture dish to allow for their unequivocal identification in the revised manuscript and Supplementary Material 1.**

 Is it true the imaging is done at the central mass? I had a difficult time understanding because there is also a section that states the coordinates images could be taken at. I’m very curious if there’s an impact of image location/bias involved. Is the origin selected by the researcher, are there methods for ensuring similarly sized clusters of cells are being imaged? Is it randomized? How many cells are removed in each?

**Unfortunately, we have been unclear about these issues. We have added all the info about the above points in Supplementary Material 1 and Supplementary Material 4 (also in response to the comment below).**

 I found this comment particularly interesting: “It is possible to use several other Voronoi generators that can be found online as freeware or in dedicated programs.” They acknowledge the existence of other modeling systems, would be valuable to know if the results are similar., i.e. the impact of the modeling choice. Even a visual demonstration to compare the resulting polygons would be a powerful addition (to answer the obvious question about validity of Voronoi method, how important is accurate cell selection, is there an impact of user experience, etc.).

**We found this comment particularly useful. We have briefly addressed this issue in the revised text and added a more detailed explanation in Supplementary Material 4.**

 Data interpretation: I think my biggest concern is lack of description of limitations/how perceivable limitations might be overcome

**We have now described the limitations of the techniques in the main text and Supplementary Materials.**

  and secondary, that the discussion and conclusion only includes the word Reelin once, in the first sentence…otherwise the paper is about animal welfare? If the focus is going to be about how to reduce animal sizes, more information should be provided about litter sizes, culling, survival of mutants, etc. and how litter survival might be a limitation for studying other proteins using this method (or not). More information would be required about how many cultures can be derived from a single animal (i.e. size of slice versus size of structure at age of collection, etc.) Either it’s about Reelin, which I think it should be, and I think is interesting, or it’s a methods paper, in which case I think it would be much more valuable to show positive results in at least 2 proteins of interest in a way that allows for comparing the cultures to really show the applications of this technique. I think it might be limited to studying proteins with a very specific subset of functions, so maybe choosing another protein with a vastly different function of Reelin would be a good choice to really highlight the value of this technique. Instead, I think re-writing the discussion and conclusion to focus on results about Reelin would be more appropriate. What can the authors infer about the possibility of Reelin application to modify Reelin-mediated deficits? What can the authors infer about Reelin’s function in regulating cell morphology, beyond simple layering/migration results? Are these results consistent with the in vivo known functions of Reelin? What does this mean? Would you expect there would be differences in non-Purkinje cells? What other sets of cells you can study with this technique? Are there basket cells, granular cells etc. in the culture that we can’t visualize? Why the cerebellum? Is there interaction between the various cells in culture (both PN to PN and PN to any other non-GFP cells)? How might you evaluate cell interactions using this model in the future? What other directions would you go? Just so much to talk about and instead we were told we can save an undetermined, unspecified number of animals…but to learn what? Instead of providing 2 highly specific proteins that could be studied with this method, why not provide a sweeping generalized set of proteins you might have interest in studying with this technique? Usually, the issue is people over-exclaim their success, but in this case, I think not enough effort is given to really dive into what this study indicates about Reelin or what else can be done with the method. If this method is so great, then there should be no shortage of things to tell the reader that you learned about Reelin in applying the technique, instead of how many fewer animals might be needed in future hypothetical studies.

**According to the comments above we have substantially rewritten the discussion that has now a section in which we take into consideration the significance of this study in terms of Reelin biology.**

 Statistics: Only issue with stats described earlier: pertaining to non-randomized imaging location (coordinates in the dish essentially) leads to selection bias. Alternative suggestions would include: a) create a grid and take an average of all polygons in multiple grid boxes to get a variety of polygon origins within the analysis. Additionally, it would be valuable to demonstrate whether or not peripheral polygons are truly similar to the central mass-related polygons, in other words, a within-culture analysis would also be valuable (is there more or less homogeneity of cultures from Reelin homo- or heterozygous mice?). Combining a perceived selection bias with unconfirmed cell selection, the reader might reasonably be concerned the results aren’t the most meaningful or generalizable.

**We have clarified these points in response to some of the previous comments in particular in Supplementary Materials 1-4. We have also added a within-culture analysis by the use of several GIS tools.**

 References (for both responses to reviewers)

 1             Jossin, Y.: ‘Reelin Functions, Mechanisms of Action and Signaling Pathways During Brain Development and Maturation’, Biomolecules, 2020, 10, (6)

 2             Mariani, J., Crepel, F., Mikoshiba, K., Changeux, J.P., and Sotelo, C.: ‘Anatomical, Physiological and Biochemical Studies of the Cerebellum from Reeler Mutant Mouse’, Philosophical Transactions of the Royal Society of London B: Biological Sciences, 1977, 281, (978), pp. 1-28

 3             Magliaro, C., Cocito, C., Bagatella, S., Merighi, A., Ahluwalia, A., and Lossi, L.: ‘The number of Purkinje neurons and their topology in the cerebellar vermis of normal and reln haplodeficient mouse’, Ann Anat, 2016, 207, pp. 68-75

 4             Ruiz i Altaba, A., Palma, V., and Dahmane, N.: ‘Hedgehog–GLI signaling and the growth of the brain’, Nature Reviews Neuroscience, 2002, 3, (1), pp. 24-33

 5             Cocito, C., Merighi, A., Giacobini, M., and Lossi, L.: ‘Alterations of Cell Proliferation and Apoptosis in the Hypoplastic Reeler Cerebellum’, Frontiers in Cellular Neuroscience, 2016, 10, pp. 141

 6             Oberdick, J., Levinthal, F., and Levinthal, C.: ‘A Purkinje cell differentiation marker shows a partial DNA sequence homology to the cellular sis/PDGF2 gene’, Neuron., 1988, 1, (5), pp. 367-376

 7             Nordquist, D., Kozak, C., and Orr, H.: ‘cDNA cloning and characterization of three genes uniquely expressed in cerebellum by Purkinje neurons’, The Journal of Neuroscience, 1988, 8, (12), pp. 4780-4789

 8             Sługocka, A., Wiaderkiewicz, J., and Barski, J.J.: ‘Genetic Targeting in Cerebellar Purkinje Cells: an Update’, Cerebellum, 2017, 16, (1), pp. 191-202

 9             Maskos, U., Kissa, K., St Cloment, C., and Brûlet, P.: ‘Retrograde trans-synaptic transfer of green fluorescent protein allows the genetic mapping of neuronal circuits in transgenic mice’, Proc Natl Acad Sci U S A, 2002, 99, (15), pp. 10120-10125

 10           Preparata, F.P., and Shamos, M.: ‘Computational Geometry: An Introduction’ (Springer New York, 1993. 1993)

 11           Yoshiki, A., and Kusakabe, M.: ‘Cerebellar histogenesis as seen in identified cells of normal-reeler mouse chimeras’, Int.J Dev Biol., 1998, 42, (5), pp. 695-700

 12           Schaefer, A., Poluch, S., and Juliano, S.: ‘Reelin is essential for neuronal migration but not for radial glial elongation in neonatal ferret cortex’, Dev Neurobiol, 2008, 68, (5), pp. 590-604

 13           Hammond, V.E., So, E., Cate, H.S., Britto, J.M., Gunnersen, J.M., and Tan, S.S.: ‘Cortical layer development and orientation is modulated by relative contributions of reelin-negative and -positive neurons in mouse chimeras’, Cereb Cortex., 2010, 20, (9), pp. 2017-2026

 14           Zhao, S., Chai, X., Bock, H.H., Brunne, B., Förster, E., and Frotscher, M.: ‘Rescue of the reeler phenotype in the dentate gyrus by wild-type coculture is mediated by lipoprotein receptors for Reelin and Disabled 1’, J Comp Neurol, 2006, 495, (1), pp. 1-9

 15           D’Arcangelo, G., Nakajima, K., Miyata, T., Ogawa, M., Mikoshiba, K., and Curran, T.: ‘Reelin Is a Secreted Glycoprotein Recognized by the CR-50 Monoclonal Antibody’, The Journal of Neuroscience, 1997, 17, (1), pp. 23-31

 16           Miyata, T., Nakajima, K., Aruga, J., Takahashi, S., Ikenaka, K., Mikoshiba, K., and Ogawa, M.: ‘Distribution of a reeler gene-related antigen in the developing cerebellum: An immunohistochemical study with an allogeneic antibody CR-50 on normal and reeler mice’, Journal of Comparative Neurology, 1996, 372, (2), pp. 215-228

 17           Galli-Resta, L., Novelli, E., Kryger, Z., Jacobs, G.H., and Reese, B.E.: ‘Modelling the mosaic organization of rod and cone photoreceptors with a minimal-spacing rule’, European Journal of Neuroscience, 1999, 11, (4), pp. 1461-1469

 18           Lagache, T., Lang, G., Sauvonnet, N., and Olivo-Marin, J.C.: ‘Analysis of the spatial organization of molecules with robust statistics’, PLoS One, 2013, 8, (12), pp. e80914

 19           Kiskowski, M.A., Hancock, J.F., and Kenworthy, A.K.: ‘On the use of Ripley's K-function and its derivatives to analyze domain size’, Biophys J, 2009, 97, (4), pp. 1095-1103

 20           Rahimi-Balaei, M., Bergen, H., Kong, J., and Marzban, H.: ‘Neuronal Migration During Development of the Cerebellum’, Frontiers in Cellular Neuroscience, 2018, 12

 21           D'Arcangelo, G.: ‘Reelin in the Years: Controlling Neuronal Migration and Maturation in the Mammalian Brain’, Advances in Neuroscience, 2014, vol. 2014, Article ID 597395, 19 pages. doi:10.1155/2014/597395

 22           Goffinet, A.M.: ‘The embryonic development of the cerebellum in normal and reeler mutant mice’, Anat Embryol (Berl), 1983, 168, (1), pp. 73-86

 23           Mikoshiba, K., Nagaike, K., Kohsaka, S., Takamatsu, K., Aoki, E., and Tsukada, Y.: ‘Developmental studies on the cerebellum from reeler mutant mouse in vivo and in vitro’, Developmental Biology, 1980, 79, (1), pp. 64-80

 24           Inoue, Y., Maeda, N., Kokubun, T., Takayama, C., Inoue, K., Terashima, T., and Mikoshiba, K.: ‘Architecture of Purkinje cells of the reeler mutant mouse observed by immunohistochemistry for the inositol 1,4,5-trisphosphate receptor protein P400’, Neuroscience Research, 1990, 8, (3), pp. 189-201

 25           D'Arcangelo, G., Miao, G.G., Chen, S.C., Soares, H.D., Morgan, J.I., and Curran, T.: ‘A protein related to extracellular matrix proteins deleted in the mouse mutant reeler’, Nature, 1995, 374, (6524), pp. 719-723

 26           Miyata, T., Nakajima, K., Mikoshiba, K., and Ogawa, M.: ‘Regulation of Purkinje Cell Alignment by Reelin as Revealed with CR-50 Antibody’, The Journal of Neuroscience, 1997, 17, (10), pp. 3599-3609

 27           Shomer, N.H., Allen-Worthington, K.H., Hickman, D.L., Jonnalagadda, M., Newsome, J.T., Slate, A.R., Valentine, H., Williams, A.M., and Wilkinson, M.: ‘Review of Rodent Euthanasia Methods’, Journal of the American Association for Laboratory Animal Science, 2020, 59, (3), pp. 242-253
